# Evaluation of the collaboration between intrinsic activity and diffusion: a descriptor for alkene epoxidation catalyzed by TS-1†

**DOI:** 10.1039/d5sc00987a

**Published:** 2025-03-01

**Authors:** Di Pan, Jiayu Yu, Ke Du, Kexin Yan, Ling Ding, Yahong Zhang, Yi Tang

**Affiliations:** a Department of Chemistry, Shanghai Key Laboratory of Molecular Catalysis and Innovative Materials, Laboratory of Advanced Materials, Collaborative Innovation Centre of Chemistry for Energy Materials (iChEM), Fudan University Shanghai 200433 P. R. China zhangyh@fudan.edu.cn yitang@fudan.edu.cn

## Abstract

The systemic evaluation of the collaboration between intrinsic activity of active sites and the substrate/product diffusion rate is a key challenge for rational optimization of reaction performance in alkene epoxidation. Herein, we constructed a series of TS-1 catalysts with adjustable local microenvironments around active sites to explore their performance in alkene epoxidation. This was achieved by controlling the contributions of classical and non-classical routines during the crystallization process. Based on the characterization of active sites, product diffusion and their alkene epoxidation activity, we proposed a descriptor, *R*_a/d_, for quantitative evaluation of the collaboration between intrinsic activity and product diffusion in TS-1 catalysts. This descriptor successfully explains the catalytic performance of various TS-1 catalysts including commercial TS-1 and guides further improvement of TS-1 in different alkene epoxidations. The optimal *R*_a/d_ is found in various epoxidations, corresponding to peak epoxidation performance across different substrates due to the well-balanced intrinsic activity and diffusion rate.

## Abstract 2

The systemic evaluation of the collaboration between intrinsic activity of active sites and the substrate/product diffusion rate is a key challenge for rational optimization of reaction performance in alkene epoxidation.

## Graphical abstract

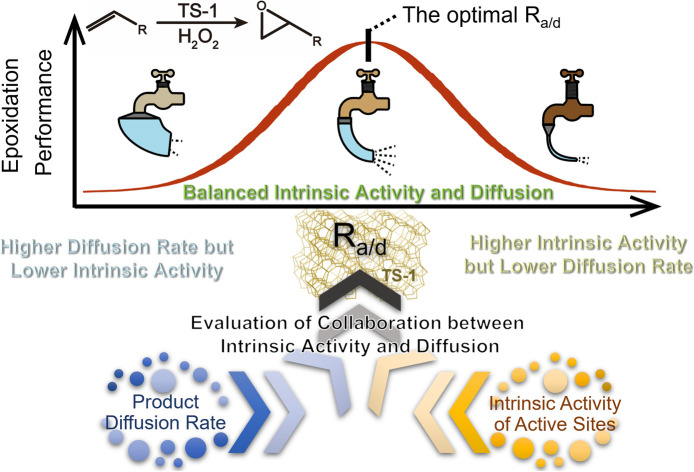

## 1. Introduction

Epoxides are in great demand as important intermediates and products in polymer, adhesive and medicine industries. Alkene epoxidation is widely recognized as a primary route for the production of epoxides. Among various epoxidation strategies, heterogeneous epoxidation catalyzed by titanosilicate using hydrogen peroxide as the oxidate has recently attracted much attention and been industrially applied in propylene epoxidation due to its mild reaction conditions, environmental friendliness, and easy recovery of catalysts.^1–5^

Optimization of titanosilicate properties has taken center stage owing to its high industrial value and potential.^6–11^ TS-1, a zeotype titanosilicate with MFI topology, is particularly notable for its industrial application in propylene epoxidation, prompting ongoing efforts to enhance its epoxidation performance.^12,13^ Considering the upper limits for directly increasing the effective content of catalyst active components, a large number of methods have been developed to enhance reaction performances by promoting intrinsic activity and improving diffusion.^14–17^ To enhance intrinsic activity, researchers have focused on modulating the microstructure of Ti active sites and internal surface hydrophilicity for better hydroperoxide activation.^18–21^ For example, hexa-coordinated Ti(OH)_4_(OSi)_2_ species, constructed by mother liquid post treatment, exhibit a catalytic activity about 8.7 times greater than that of the known tetra-coordinated framework Ti(OSi)_4_ sites.^22^ Additionally, controlling batch fluoride ion concentration results in hydrophilic (*i.e.*, (SiOH)_*x*_ defect-rich) Ti-MFI-OH catalysts, which display epoxidation turnover rates over an order of magnitude higher than those of hydrophobic TS-1 catalysts.^23^ To improve diffusion in catalysts, strategies such as morphology control and mesopore construction are employed.^24,25^ Hollow TS-1, made by post-synthesis with a solvent-free method utilizing NH_4_HCO_3_ and tetrapropylammounium bromide as selective etching agents, achieves nearly double conversion in 1-hexene epoxidation.^26^ Hierarchical TS-1 crystals, synthesized by a carbon material templated dry gel crystallization strategy, demonstrate superior catalytic performance in allyl chloride (ACH) epoxidation.^27^

Nevertheless, our previous work indicates that enhancing the intrinsic activity of TS-1 can promote ACH oxidation but suppresses 1-octene (1-OCT) oxidation.^28^ This suggests that both intrinsic activity and diffusion influence epoxidation results, and mismatches between either single intrinsic activity or diffusion and apparent epoxidation performance can occur. Thus, the isolated optimization of either intrinsic activity or diffusion is insufficient for understanding and modulating complex catalytic systems. Therefore, a systemic quantitative analysis of intrinsic activity and diffusion is required for rational reaction optimization rather than viewing intrinsic activity or diffusion in isolation. Considering the difficulty to summarize reliable roles of catalysts with large structure and catalytic property diversities, it is necessary to construct a series of characteristic TS-1 catalysts. Elaborating the desired catalyst by switching the crystallization mechanism involving classical and non-classical routines has brought renewed attention in virtue of recent systemic investigation into crystallization mechanisms.^29–31^ Active site structures of catalysts and diffusion properties are modulated *via* adjusting crystallization routines,^32,33^ and hence a catalyst platform which is suitable for comprehensive evaluation could be established.

In this study, we develop a strategy for fine regulation between classical and non-classical routines in a TS-1 crystallization system based on application of TS-1 zeolite subcrystals (SCs). Through controlling the contributions of classical and non-classical routines during the crystallization process, we construct a series of TS-1 catalysts with precisely adjustable local microenvironments around active sites, including the structure of Ti sites, space-confined structure and hydrophilicity. Based on the obtained series of TS-1 catalysts, we propose a descriptor, *R*_a/d_, for quantitatively evaluating the collaboration between intrinsic activity of active sites and product diffusion in alkene epoxidation catalyzed by these TS-1 catalysts. *R*_a/d_ is defined as the natural logarithm of the ratio of reaction kinetics with the mere consideration of activation energy and the diffusion rate. By calculating *R*_a/d_ of TS-1 catalysts in various alkene epoxidation, turnover numbers (TONs) *versus R*_a/d_ plots are found to be volcano type curves and optimal *R*_a/d_ values in each epoxidation are revealed, which enables the analysis and further optimization of epoxidation performance of various catalysts including commercial TS-1 based on the deviation of the *R*_a/d_ of a catalyst from the optimal value.

## 2. Experimental section

### 2.1. Chemical and materials

All of the following reagents are directly used without further purification. Sillica sol (SiO_2_, 40 wt% in water, HS-40) was purchased from Ludox. Titanic sulfate (Ti(SO_4_)_2_, 98%), 1-hexene (C_6_H_12_, 99%), allyl chloride (C_3_H_5_Cl, 98%), 1-octene (C_8_H_16_, 98%), 1,2-epoxyhexane (C_6_H_12_O, 96%), epichlorohydrin (C_3_H_5_ClO, 99.7%), 1,2-epoxyoctane (C_8_H_16_O, 95%), 1,3,5-trimethylbenzene (C_9_H_12_, 97%), ethylamine (C_2_H_7_N, 99%) and commercial TS-1 called TS-1-MKL (Si/Ti = 40) were purchased from Macklin. Tetrapropylammonium hydroxide (TPAOH, 25 wt%), and ceric sulfate (Ce(SO_4_)_2_, 98%) were purchased from OKA. Hydroperoxide (H_2_O_2_, 30 wt%), methanol (CH_3_OH, 99.8%), sodium hydroxide (NaOH, 99%) and sulfuric acid (H_2_SO_4_, 98 wt%) were purchased from Hushi. Commercial TS-1 called TS-1-NK (Si/Ti = 34) was purchased from NKC. Commercial TS-1 called TS-1-RD (Si/Ti = 47) was purchased from Raodong (Liaoning) New Material Co. , Ltd. Commercial TS-1 called TS-1-YK (Si/Ti = 33) was purchased from Qingdao Yuanke Catalyst Co., Ltd. Deionized (DI) water (H_2_O) was purchased from Watsons. A dialysis tube (Spectra Por 3) was purchased from Spectrum Laboratories.

### 2.2. Synthesis of TS-1 samples with different non-classical crystallization contributions

The TS-1 samples were hydrothermally synthesized from mixtures with different ratios of TS-1 initial synthetic sol and TS-1 SC sol (Table S1†). The synthesis of TS-1 initial synthetic sol and TS-1-SC sol is shown in the ESI.† The mixture previously stirred for 30 min in a 30 mL explosion-proof glass tube was treated at 433 K for 360 min in an Anton Paar monowave 300 microwave synthesizer. Afterward, the product was recovered by centrifugation, washed with distilled water, dried at 353 K overnight, and finally calcined at 823 K for 6 h. The obtained TS-1 samples were named TS-1-43, TS-1-65, TS-1-71, TS-1-93 and TS-1-100 according to the non-classical crystallization contributions calculated from TS-1 SC content in their synthesis batches listed in the ESI.†

### 2.3. Synthesis of TS-1-NK-Na and TS-1-MKL-Na

TS-1-NK or TS-1-MKL was suspended in an aqueous solution that contained NaOH in a PP tube; a typical aqueous solution has the following molar composition: 1.0SiO_2_ : 0.15NaOH : 333H_2_O. Then the mixture was stirred for two hours at 353 K. Then the product was recovered by filtration and drying. After ammonium exchange in 1 M NH_4_NO_3_ solution, the product was recovered by filtration, drying, and calcination at 823 K. The calcined sample was denoted as TS-1-NK-Na or TS-1-MKL-Na. The Si/Ti ratio in TS-1-NK-Na or TS-1-MKL-Na was 32 and 40, respectively.

### 2.4. Synthesis of TS-1-43-EA

TS-1-43 was suspended in an aqueous solution that contained ethylamine (EA) in a PTFE liner; a typical aqueous solution has the following molar composition: 1.0SiO_2_ : 0.07EA : 15H_2_O. Then the mixture was stirred for half an hour. The PTFE liner was placed in a stainless steel autoclave and crystallized at 433 K for 48 h. Then the product was recovered by filtration, drying, and calcination at 823 K. The calcined sample was denoted as TS-1-43-EA. The Si/Ti ratio in TS-1-43-EA was 75.

### 2.5. Characterization

Dynamic light scattering (DLS) measurements were employed to monitor the particle size change during TS-1 zeolite synthesis and aggregation crystallization of TS-1 subcrystals on a Malvern Zetasizer Nano-ZS90 instrument. Before testing, the liquid samples were diluted and dispersed, and tested 10 times to get accurate data. The scanning electron microscope (SEM) photos were taken on an FE-SEM-4800-1, which were used to visualize the morphologies and structure features of the samples. Hard X-ray diffraction (HAXRD) data were collected at the BL14B1 beamline (*λ* = 0.6887 Å) at Shanghai Synchrotron Radiation Facility. Data refinements were performed using the FullProf software to determine the cell parameters and Ti location of the products. The adsorption–desorption isotherms were acquired on a Quantachrome iQ-2 instrument by Ar-sorption experiments at 87 K after outgassing at 573 K for 7 h, and textural parameters were calculated by the Brunauer–Emmett–Teller (BET) method and *t*-plot method. The pore size distributions were also calculated by the NLDFT method. Simulations of argon adsorption isotherms were calculated by the RASPA software with the Monte Carlo method. Thermogravimetric analysis (TG) and differential scanning calorimetry (DSC) plots were collected on an SDT 650 from 30 to 800 °C at a heating rate of 10 °C min^−1^ under a flow (100 mL min^−1^) of high purity air. The sorption isotherms and corresponding dynamics data of water, methanol, 1-hexene, 1,2-epoxyhexane, allyl chloride and epichlorohydrin were collected by using an IGA100B. The Fourier transform infrared spectroscopy (FT-IR) spectra were recorded as follows: a self-supported wafer (7 mg and a radius of 3.5 mm) was set in an *in situ* cell with CaF_2_ windows connected with a vacuum system. After the sample was evacuated at 723 K for 2 h, the vacuum FT-IR spectra were recorded by using a Bruker Invenio S. ^1^H solid-state magic angle spinning nuclear magnetic resonance (SS MAS NMR) spectra were collected on a Bruker 400WB AVANCE III after activating the samples in a vacuum at 573 K for 7 h in advance. Inductively coupled plasma atomic emission spectroscopy (ICP-AES) was performed on a PE-8000 atomic emission spectrometer for evaluating the bulk element content of the product. X-ray photoelectron spectroscopy (XPS) experiments were conducted on a Thermo Fisher Scientific K-Alpha (Al-Kα) to analyze the element content conditions at the surface of products. The diffuse reflection ultraviolet-visible (DR UV-vis) spectra were obtained on a Lambda 650S. X-ray absorption fine structure (XAFS) data including extended X-ray absorption fine structure (EXAFS) were collected at the 4B7A Beamline at the Beijing Synchrotron Radiation Facility. Data processing was performed using the Athena, Artemis and Matlab software.

### 2.6. Catalytic reactions

The epoxidation of alkene with H_2_O_2_ was carried out in a 25 mL eggplant-shaped flask sealed by a rubber stopper under vigorous stirring. The substrate included allyl chloride and 1-hexene. In a typical run, 10 mL of methanol, 10 mmol of alkene, 10 mmol of H_2_O_2_ (30 wt%), 50 mg of catalyst and 0.5 g of 1,3,5-trimethylbenzene were mixed in a bottle, and the reaction was run under magnetic stirring at 333 K for 2 h for allyl chloride and 6 h for 1-hexene and 1-octene. For the reaction catalyzed by catalysts pretreated with 1,2-epoxyhexane and methanol, 50 mg of fresh catalysts were placed in 1 mL of 10 wt% 1,2-epoxyhexane in 1,3,5-trimethylbenzene solution and placed at room temperature for a certain time before recovery by centrifugation, and washed with methanol three times. After that, the reaction was carried out as usual. For reaction kinetics measurements, the temperatures were changed from 303 to 333 K at 10 K intervals while other reaction parameters were kept the same. After the completion of the reaction, the liquid products were collected by filtration and analyzed with a gas chromatograph (GC-2010 Plus), equipped with a 30 m capillary column (DB-WAX) and an FID detector by using 1,3,5-trimethylbenzene as the internal standard. The unconverted H_2_O_2_ was calculated by titration with 0.05 M Ce(SO_4_)_2_ in 10 wt% H_2_SO_4_. The turnover number (TON), turnover frequency (TOF), H_2_O_2_ efficiency and *R*_a/d_ were calculated based on the following formulae. The TON and TOF were calculated based on alkene conversion and Ti content. H_2_O_2_ efficiency was calculated based on alkene conversion and the remaining H_2_O_2_ amount. *R*_a/d_ was calculated based on the activation energy and diffusion rate for each Ti site.
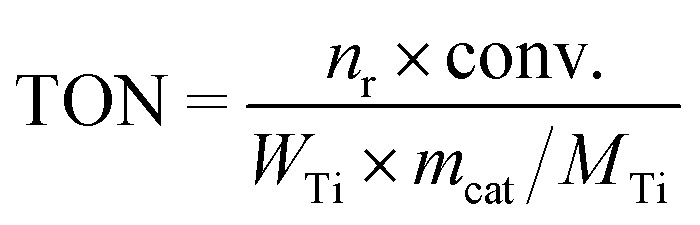

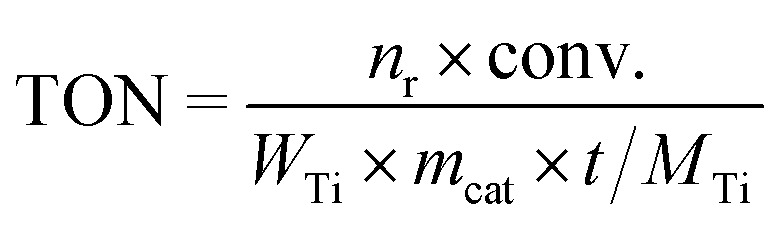
in which *n*_r_ is the amount of the reactant alkene. Conv. is the conversion of the reactant alkene. *W*_Ti_ is the Ti weight percentage of the catalyst. *m*_cat_ is the mass of the catalyst. *t* is the reaction time. *M*_Ti_ is the relative atomic weight of Ti.
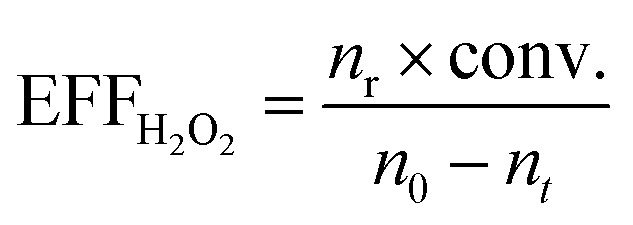
where *n*_r_ is the amount of the reactant alkene. Conv. is the conversion of the reactant alkene. *n*_0_ is the initial amount of H_2_O_2_. *n*_*t*_ is the amount of unconverted H_2_O_2_.
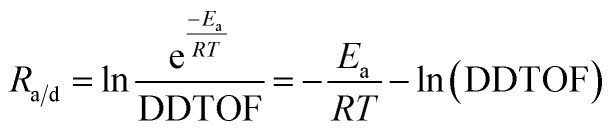
where *E*_a_ is the activation energy. *R* is the molar gas constant. *T* is the temperature, and here it is 333 K. DDTOF is the theoretical desorption diffusion turnover frequency here in the gas phase.

## 3. Results and discussion

### 3.1. Preparation and characterization of a series of TS-1 catalysts

Subcrystals (SCs) are considered indispensable intermediates during the crystallization process in MFI clear sol synthesis.^34^ As uniform nanoparticles, SCs are the nuclei with high short-range order, but lack long-range order, enabling oriented attachment without the aid of extra structure-directing agents (SDA) and soluble silica. In other words, they can crystallize through a solely non-classical routine.^28,34,35^ When previously prepared and extracted TS-1 SCs shown in Fig. S1† are introduced into the batch composition, the contribution of the non-classical crystallization routine, *i.e.*, the oriented attachment of particles, increases in the system. Herein, the contribution of non-classical crystallization is adjusted from 43%, 71%, and 93% to 100% by changing the addition proportion of TS-1 SC particles in the initial synthetic sol as shown in Table S1†. This results in the formation of four distinct TS-1 samples, namely TS-1-43, TS-1-71, TS-1-93 and TS-1-100, respectively. These samples provide a series of candidates for the exploration of the collaboration between intrinsic activity of active sites and the diffusion limitation of the substrate/product in alkene epoxidation.

**Fig. 1:**
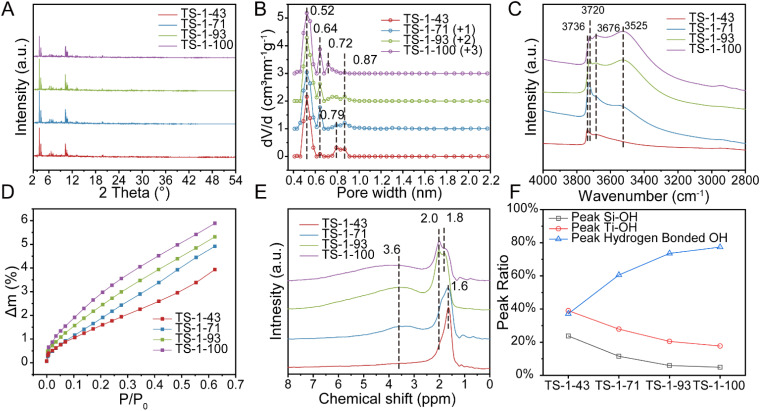
XRD patterns (A) and pore size distribution plots (B) of TS-1 samples. The wavelength of the X-ray is 0.6887 Å. Pore size distribution plots are calculated from Ar adsorption–desorption isotherms by the DFT model. Vacuum IR spectra (C), adsorption isotherms of water (D), ^1^H SS MAS NMR spectra (E) and hydroxyl group ratio of Si–OH, Ti–OH and hydrogen bonded OH groups (F) of TS-1 samples.

X-ray diffraction (XRD) patterns shown in Fig. 1A confirm that all the samples show a typical MFI-type structure with similar crystallinity. Further structural refinements^36^ reveal the minor difference in the cell parameters between TS-1 samples and the random Ti location in all the samples as illustrated in Fig. S2–S5 and Table S2†. According to argon adsorption–desorption isotherms and the calculated texture properties as shown in Fig. S6† and Table 1, there is a minor difference in micropore volume and micropore surface area despite the changed morphology with the nearly constant crystal size displayed in Fig. S7.† The calculated pore size distributions shown in Fig. 1B show micropores with a width of 0.52 nm in all the samples, confirming their MFI structures.^37–39^ From TS-1-43 to TS-1-71, some of the restricted cross orifices with a width of 0.79 nm contract to 0.64 nm without a change in the rest of the cross orifices with a width of 0.87 nm.^40,41^ The width of the remaining cross orifices with a width of 0.87 nm gradually moves to 0.72 nm from TS-1-71 to TS-1-100. This contraction is further proved by the higher pyrolysis temperature of SDA from TS-1-43 to TS-1-100 detected by thermogravimetric analysis (TG) and differential scanning calorimetry (DSC) tests illustrated in Fig. S8.† Argon adsorption isotherm simulations of microstructures with various space clogs are provided in Fig. S9–S11,† which indicate that the contracted space-confined structure originates from the partial blockage at the outer side of the sinusoidal tunnel of the cross orifice. Fourier transform infrared spectroscopy (FT-IR) spectra, collected after activation in a vacuum, clarify the presence of the hydroxyl (OH) defects as shown in Fig. 1C. Bands at 3676 cm^−1^, observed in all the samples,^42,43^ account for Ti–OH groups. The band at 3736 cm^−1^ shifts to 3720 cm^−1^ from TS-1-43 to TS-1-100, demonstrating fewer external OH groups and more internal OH groups.^44–47^ The intensities of broad bands at 3525 cm^−1^, representing hydrogen-bonded OH groups, increase from TS-1-43 to TS-1-100,^48,49^ indicating the emergence of more OH-rich structures. The shifts in OH types and the increase in OH-rich structures imply hydrophilicity order of the samples,^50,51^*i.e.*, TS-1-43 < TS-1-71 < TS-1-93 < TS-1-100, as evidenced by water and methanol adsorption isotherms displayed in Fig. 1D and S12,† respectively. As shown in Fig. 1E, ^1^H solid-state magic angle spinning nuclear magnetic resonance (^1^H SS MAS NMR) spectra show two fixed bands in all the samples at 2.0 and 3.6 ppm, referring to Ti–OH and hydrogen-bonded OH, respectively.^28,42,47,52^ Slight downfield shifts of bands corresponding to Si–OH groups, from 1.6 ppm to 1.8 ppm, are observed due to the dominant OH groups changing from external ones to internal ones from TS-1-43 to TS-1-100. Further quantitative analysis points out that the higher non-classical crystallization contribution corresponds to a larger ratio of hydrogen-bonded OH groups among all the OH groups as illustrated in Fig. 1F and S13†, matching with the conclusion from FT-IR in Fig. 1C. The results of synergic analyses of inductively coupled plasma atomic emission spectroscopy (ICP-AES) and X-ray photoelectron spectroscopy (XPS) listed in Table 1 show that all the TS-1 samples exhibit lower Si/Ti ratios compared with the feeding Si/Ti ratio.^53,54^ In addition, slight surface Si enrichment is observed in TS-1-43, TS-1-71 and TS-1-93, which may be a result of Oswald ripening triggered by classical crystallization.^55^

**Table 1:** The summary of the growth process and final products at different H_2_O/SiO_2_ ratios for MFI zeolite

Sample	*S*_micro_ a (m^2^ g^−1^)	*S*_exter_ b (m^2^ g^−1^)	*V*_micro_ b (cm^3^ g^−1^)	*V*_meso_ c (cm^3^ g^−1^)	Si/Ti_bulk_^d^	Si/Ti_surface_^e^
TS-1-43	362	81	0.156	0.109	75	80
TS-1-71	386	103	0.141	0.094	65	77
TS-1-93	408	97	0.146	0.098	50	67
TS-1-100	396	91	0.141	0.091	40	42

a *S*_micro_ (micropore area) was calculated using the BET method.

b *S*_exter_ (external surface area) and *V*_micro_ (micropore volume) were calculated using the *t*-plot method.

c *V*_meso_ (mesopore volume) was calculated from the total pore volume.

d Given by ICP-AES.

e Given by XPS.

**Fig. 2:**
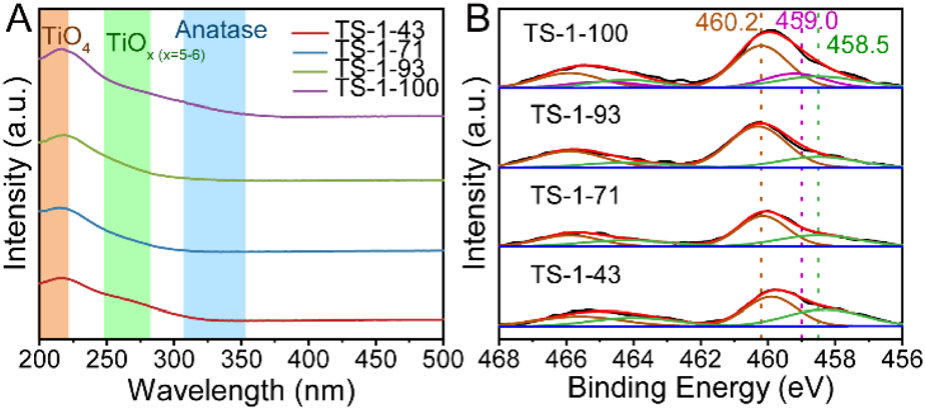
UV-vis spectra (A) and XPS spectra (B) of TS-1 samples.

As shown in Fig. 2A, in diffuse reflection ultraviolet-visible (DR UV-vis) spectra, two main bands at 205 and 265 nm are observed for all samples, which are assigned to tetrahedral Ti sites [TiO_4_] and high-coordinated Ti sites [TiO_*x*_] (*x* = 5–6) in the framework, respectively.^22,26,56^ The absence of a band at around 330 nm confirms the absence of anatase TiO_2_ in all TS-1 samples.^57,58^ The relative ratio of Ti sites is further calculated based on the Gaussian fitting method listed in Fig. S14 and Table S3.† The integration of Ti species into the framework is facilitated from TS-1-43 to TS-1-93, as shown by the slightly increasing proportion of [TiO_4_] sites. However, in TS-1-100, the content of [TiO_*x*_] (*x* = 5–6) increases, implying the formation of framework pentahedral Ti sites [TiO_5_].^28^ This is confirmed by the appearance of an extra peak at 459.0 eV in the XPS spectra displayed in Fig. 2B, which is only present in TS-1-100. The [TiO_4_] and octahedral Ti sites [TiO_6_] in the framework are also detected in all the samples corresponding to bands at 460.2 and 458.5 eV respectively.^9,28,59^ The distributions of Ti sites with different coordination environments calculated from DR UV-vis and XPS spectra match well with each other as shown in Table S3†. Specifically, from TS-1-43 to TS-1-93, more Ti is present as [TiO_4_] and less as [TiO_6_], while [TiO_5_] springs up only in TS-1-100, which is further verified by the wavelet-transform extended X-ray absorption fine structure (WT-EXAFS) spectrum^28,60^ illustrated in Fig. S15.†

Overall, the systemic characterization studies above have clarified that the physicochemical properties of TS-1 samples are tailored meticulously by simply controlling the contribution of classical and non-classical crystallization. Together with the enhanced non-classical crystallization contribution from TS-1-43 to TS-1-100, the promoted space-confined structure contraction and hydrophilicity of the microenvironment are obtained. Moreover, Ti species tends to exist more as framework [TiO_4_] from TS-1-43 to TS-1-93, despite the amplified presence of [TiO_5_] in TS-1-100.

### 3.2. Evaluation of epoxidation performance based on a descriptor, *R*_a/d_

Given that the above structural factors in TS-1 samples, which are gradually modulated, are believed to influence either intrinsic activity and diffusion, or both,^22,61,62^ and consequently affect epoxidation performance,^49,63,64^ a general descriptor is expected to be proposed for systemically evaluating the collaboration between intrinsic activity and diffusion. This descriptor, proposed based on the established catalyst platform, aims to facilitate the analysis and estimation of reaction results of different TS-1 catalysts with various alkene substrates.

**Fig. 3:**
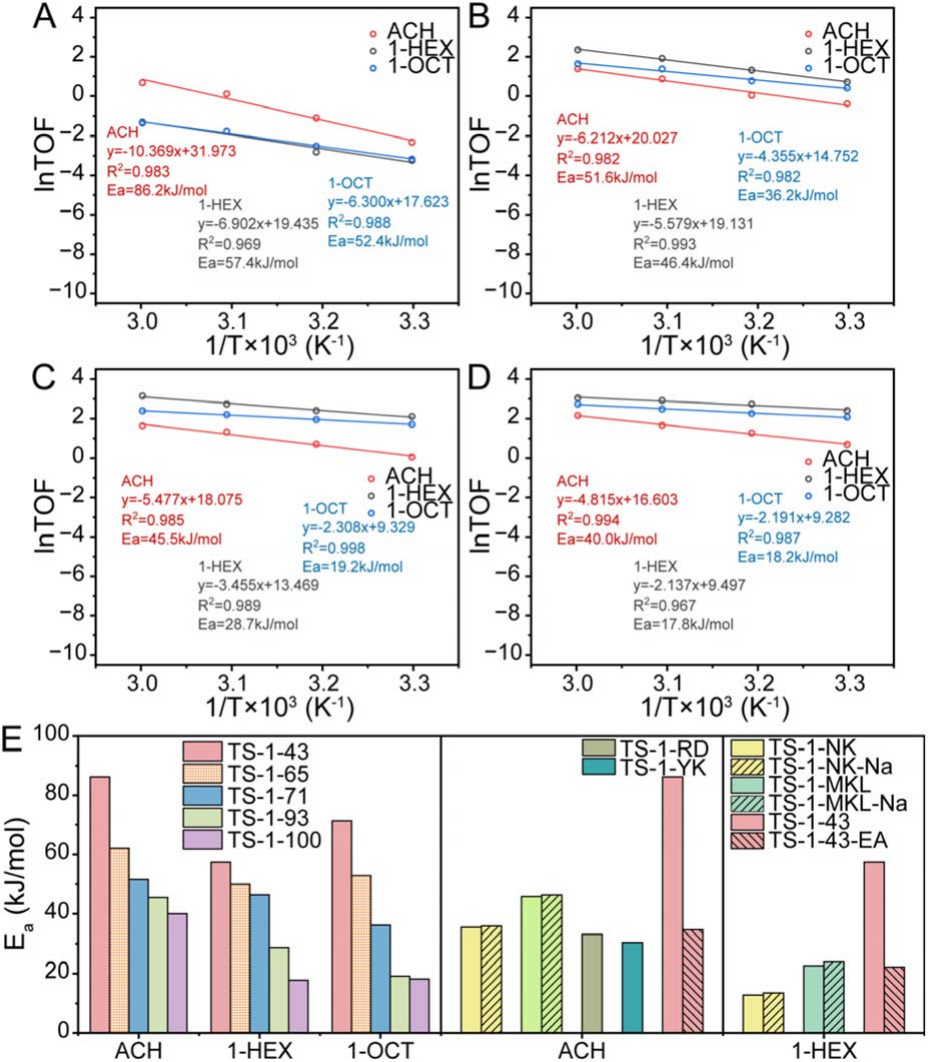
Arrhenius plots of ACH, 1-HEX and 1-OCT epoxidation in TS-1-43 (A), TS-1-71 (B), TS-1-93 (C) and TS-1-100 (D) and activation energy of ACH, 1-HEX and 1-OCT epoxidation (E). TS-1-43 is mentioned again next to TS-1-43-EA for reflecting effects of post treatments. Reaction conditions: catalyst 50 mg, alkene 10 mmol, H_2_O_2_ 10 mmol, methanol 10 mL. The temperatures change from 303 to 333 K at 10 K intervals.

The intrinsic activities of the four TS-1 samples are evaluated from apparent activation energy through monitoring ACH, 1-HEX and 1-OCT epoxidation at different temperatures as shown in Fig. 3 and S16–S19.† The activation energy of most alkene epoxidations decreases from TS-1-43 to TS-1-100. However, as shown in Table S3,† from TS-1-43 to TS-1-93, more Ti sites exist as [TiO_4_] with lower activity rather than high-performance [TiO_6_], and the intrinsic activity still increases accordingly. Such a reversal to the expected result is attributed to the enhanced hydrophilicity from TS-1-43 to TS-1-93, which facilitates epoxidation and overshadows the negative effect from the changed Ti site composition.^23,61–63,65^ From TS-1-93 to TS-1-100, the introduction of highly active [TiO_5_] greatly promotes the intrinsic activity, although the evolution of the hydrophilic microenvironment is not as significant as that from TS-1-43 to TS-1-71 and then to TS-1-93. The tendency of activation energy changes with different TS-1 microenvironments remaining consistent regardless of the alkene length. However, longer alkene molecules generally exhibit a lower reaction barrier due to the greater chemical stability of short chain alkenes, which is also illustrated in Fig. 3.

**Fig. 4:**
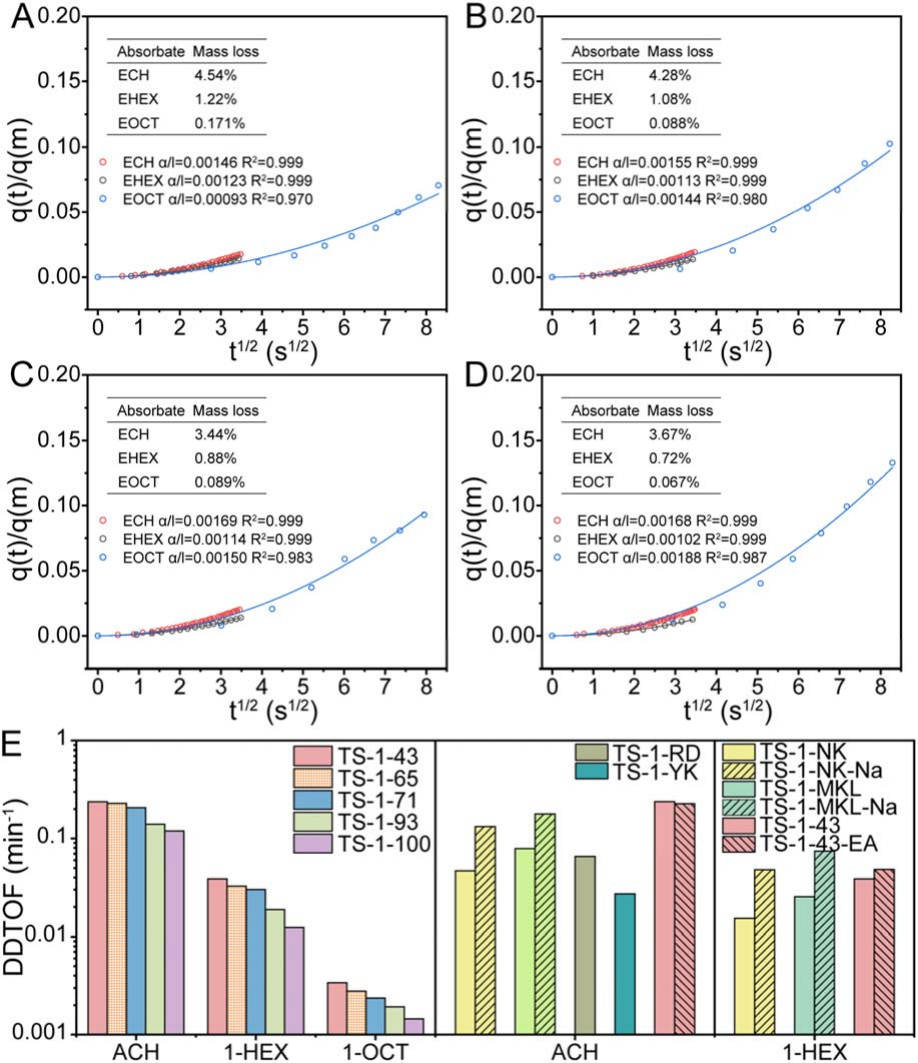
Desorption kinetic uptake curves and kinetic fitting profiles with a quadratic function of ECH, EHEX and EOCT in a short time domain in TS-1-43 (A), TS-1-71 (B), TS-1-93 (C) and TS-1-100 (D) and DDTOF of epoxides of TS-1 catalysts (E). DDTOF = *α*/l × (Δ*m* × *M*_Ti_)/(*M*_adsorbate_ × *W*_Ti_). Δ*m* is the percentage of mass loss during desorption. *M*_Ti_ is the relative atomic mass of Ti. *M*_adsorbate_ is the relative molecular mass of the adsorbate. *W*_Ti_ is the Ti weight percentage of the adsorbent. The pressure range of desorption kinetic uptake curves of ECH and EHEX is from 0.26 mbar to 0. The pressure range of desorption kinetic uptake curves of EOCT from 2.27 mbar to 0. TS-1-43 is mentioned again next to TS-1-43-EA for reflecting effects of post treatments.

The diffusion behaviors of reactants and products in the four TS-1 samples are analyzed from adsorption–desorption isotherms and their corresponding adsorption or desorption dynamics displayed in Fig. S20–S27.† The desorption rates were consistently slower than the adsorption rates for all adsorbates as listed in Table S4†. Considering that the epoxidation active centers are Ti sites, it is rational to calculate the diffusion rate based on Ti sites while the structures of Ti sites play a less critical role owing to their scarce content in contrast to the tunnel structure of the framework in diffusion. Therefore, a theoretical desorption diffusion turnover frequency (DDTOF) is calculated by assuming that diffusion is initiated from every single Ti site. This calculation reflects the theoretical maximum desorption diffusion rate in samples regardless of the epoxidation process and other molecules such as solvent molecules. The significantly slower desorption rate of epoxides shown in Fig. 4 than that of corresponding alkenes shown in Fig. S28† indicates that the desorption dynamics of epoxides is the rate-determining step among various diffusion processes. Moreover, the results of calculation show that the DDTOFs of all the epoxides decrease monotonously from TS-1-43 to TS-1-100. This decline reveals increasing difficulty in removing epoxides from TS-1 samples with higher hydrophilicity and narrower space-confined structures due to the stronger interaction between strong polar epoxides and the pore walls as well as harder diffusion in contracted tunnels when portioning the diffusion potential of the bulk TS-1 to each Ti site. The DDTOF values are found to be higher for epichlorohydrin (ECH) than for 1,2-epoxyhexane (EHEX), and higher for EHEX than for 1,2-epoxyoctane (EOCT), corresponding to the increasing molecule length from ECH to EOCT. Therefore, given that the epoxide desorption diffusion is the rate-determining diffusion, less diffusion resistance is expected in ACH epoxidation compared to 1-HEX epoxidation, while the most intense diffusion limitation is observed in 1-OCT epoxidation.

The liquid phase breakthrough curves of 1-HEX are collected to simulate desorption in practical epoxidation, accounting for the difference between gas phase diffusion measurement and practical diffusion in the liquid phase. As shown in Fig. S29–S33,† calculated DDTOFs indicate that though the 1-HEX desorption diffusion rates change under different desorption conditions, the change trends of DDTOFs among all the catalysts remains the same as those in gas phase diffusion. This consistency suggests that comparing practical diffusion behaviors of TS-1 catalysts in epoxidation could be effectively achieved by measuring the diffusion dynamics of pure adsorbates in the gas phase.

For quantitative evaluation of collaboration of intrinsic activity and product diffusion in different epoxidation reactions, a descriptor, *R*_a/d_, is proposed, which is defined as the sum of −*E*_a_/*RT* and −ln(DDTOF). The *R*_a/d_ value stands for the extent of collaboration between intrinsic activity and diffusion, where intrinsic activity is represented by −*E*_a_/*RT*, the reaction rate constants regardless of preexponential factors, and diffusion is represented by −ln(DDTOF) of epoxides calculated from desorption dynamics in the gas phase. From the definition of *R*_a/d_, a low *R*_a/d_ shows the failure of intrinsic activity in keeping up with diffusion potential and a high *R*_a/d_ indicates the diffusion restriction on intrinsic activity. As for all the epoxidation reactions displayed in Fig. S34,†*R*_a/d_ keeps increasing from TS-1-43 to TS-1-100 owing to the enhanced intrinsic activity and suppressed diffusion. However, the absolute *R*_a/d_ values of catalysts vary across different alkene epoxidations, revealing the specific demand in different epoxidation systems. Low *R*_a/d_ values are found in ACH epoxidation, followed by 1-HEX epoxidation, and the highest *R*_a/d_ values in 1-OCT epoxidation, which reveal a gradual increase in diffusion limitation due to the increasing molecular size. An exception is found in TS-1-43, where the *R*_a/d_ in 1-HEX epoxidation is higher than that in 1-OCT epoxidation, attributed to the increased activation energy shown above in Fig. 3. Moreover, an optimal *R*_a/d_ value is expected for each specific reaction, which refers to the best collaboration between intrinsic activity and diffusion. The closer the *R*_a/d_ value of a catalyst is to this optimal *R*_a/d_, the better its reaction performance should be.

**Fig. 5:**
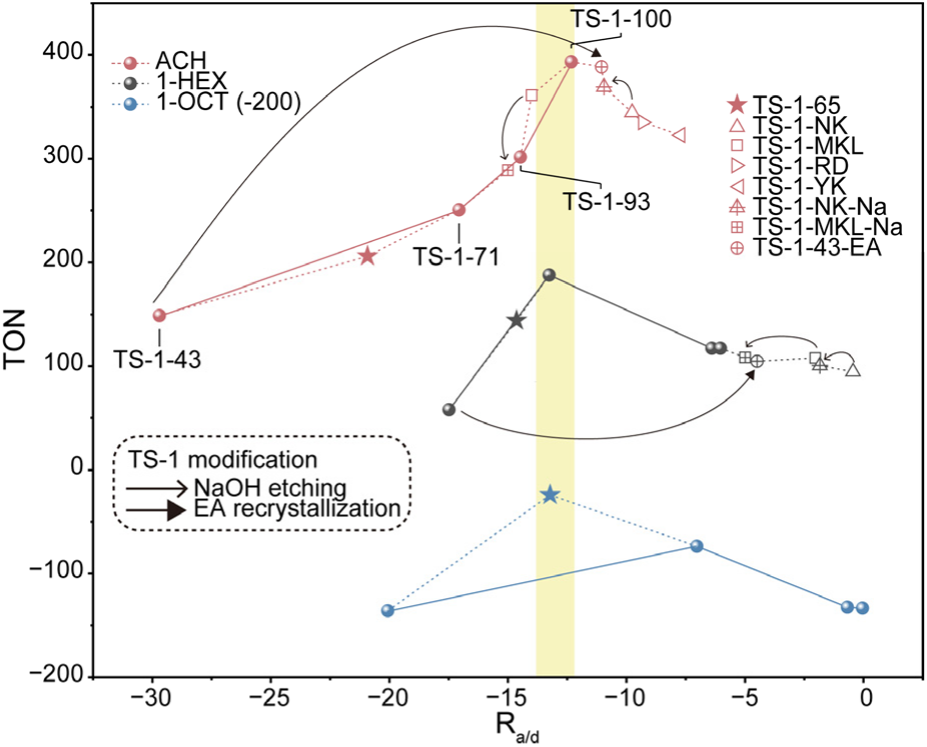
TON in alkene epoxidation *versus R*_a/d_ of TS-1 catalysts. *R*_a/d_ = −*E*_a_/*RT* − ln(DDTOF). The yellow region shows the optimal *R*_a/d_ value approximately. The star, hollow square and hollow triangle refer to TS-1-65, TS-1-MKL, TS-1-NK, TS-1-RD and TS-1-YK in alkene epoxidation. The hollow triangle, hollow square and hollow circle with a cross refer to TS-1-NK-Na, TS-1-MKL-Na and TS-1-43-EA in alkene epoxidation. Line arrows and triangular arrows indicate the change in *R*_a/d_ values and epoxidation performance of TS-1 after NaOH etching and ethylamine recrystallization, respectively. Reaction conditions: catalyst 50 mg, alkene 10 mmol, H_2_O_2_ 10 mmol, methanol 10 mL, temperature 333 K. The reaction lasts for 2 h for ACH epoxidation and 6 h for 1-HEX and 1-OCT epoxidation.

The catalytic performances of various TS-1 catalysts in ACH, 1-HEX and 1-OCT epoxidation are summarized with the corresponding *R*_a/d_ values in Fig. 5. In 1-HEX epoxidation, TS-1-71 exhibits the highest TON (about 200) among the TS-1 samples, which is about four and two times higher than that of TS-1-43 and TS-1-100, respectively, with similar EHEX selectivity exceeding 80%, as shown in Fig. S35.† The TON values of TS-1 samples represent a volcano-shaped curve with increasing *R*_a/d_, indicating the existence of an optimal *R*_a/d_ in 1-HEX epoxidation. In the case of ACH epoxidation, a volcano-shaped curve of TON *versus R*_a/d_ is not observed, as the TON increases with increasing *R*_a/d_. However, the *R*_a/d_ of TS-1-100 with the highest catalytic activity, is close to the optimal *R*_a/d_ determined for 1-HEX epoxidation. To further verify the existence of an optimal *R*_a/d_ in ACH epoxidation, four highly active commercial TS-1 catalysts, TS-1-MKL (Si/Ti = 40), TS-1-NK (Si/Ti = 34), TS-1-RD (Si/Ti = 47) and TS-1-YK (Si/Ti = 33), are applied in alkene epoxidation. The *R*_a/d_ values of these commercial catalysts are calculated from the activation energy and epoxide DDTOF in the ACH epoxidation reaction as shown in Fig. S36–S43†. In the case of ACH epoxidation, as depicted in Fig. 5, the *R*_a/d_ values of all commercial TS-1, −14.0, −9.8, −9.2 and −7.3, just fall on either side of that of the optimal TS-1-100 sample. These values correspond to suboptimal epoxidation performance in ACH, thereby providing strong support for the existence of an optimal *R*_a/d_ value. Additionally, it is worth noting that for 1-HEX epoxidation, the *R*_a/d_ values of TS-1-MKL and TS-1-NK (−2.0 and −0.4, respectively) correlate with unsatisfactory catalytic performances, aligning well with the TON *versus R*_a/d_ trend observed across various TS-1 catalysts in Fig. 5.

However, in 1-OCT epoxidation, among the four TS-1 samples, the highest TON does not seem to fall within the above optimal *R*_a/d_ range. This suggests that a sample with an optimal *R*_a/d_ is not yet achieved in 1-OCT epoxidation. The prediction according to the optimal *R*_a/d_ obtained in 1-HEX epoxidation indicates that in 1-OCT epoxidation, the optimal sample should be located between TS-1-71 and TS-1-43. To confirm this, an extra TS-1 catalyst, TS-1-65, is prepared and applied in alkene epoxidations. TS-1-65 is synthesized by controlling the non-classical contribution to 65% with a Si/Ti ratio of 64. As expected, the *R*_a/d_ values of TS-1-65 in all three epoxidation reactions (ACH, 1-HEX, and 1-OCT) turn out to be between those of TS-1-43 and TS-1-71 after measuring activation energy and epoxide DDOTOF which is shown in Fig. S44 and S45.† Notably, the *R*_a/d_ of TS-1-65 in 1-OCT epoxidation is located at −13.2, closely matching the optimal *R*_a/d_ values observed in ACH and 1-HEX epoxidations. Meanwhile, the TON of TS-1-65 displays the highest TON (over 170) among the five catalysts, which is about three times as much as that of TS-1-43 and TS-1-100. *R*_a/d_ values of TS-1-65 in ACH (−20.9) and 1-HEX (−14.6) epoxidations are further from the optimal *R*_a/d_ compared to *R*_a/d_ of TS-1-71, yet closer to the optimal *R*_a/d_ than that of TS-1-43. Consequently, the TON of TS-1-65 in ACH and 1-HEX epoxidation was observed to fall between that of TS-1-71 and TS-1-43, as illustrated in Fig. 5. These results confirm the predictive utility of *R*_a/d_ in assessing and optimizing catalytic performance.

Across the three alkene epoxidations mentioned above, the TON *versus R*_a/d_ plots all show volcano-shaped curves, indicating that the catalytic performance is unfavorable at either lower and higher *R*_a/d_, and the best performances are only reached at a moderate *R*_a/d_. So far, we can confirm that the *R*_a/d_ proposed by us can serve as a descriptor to analyze and predict epoxidation performance of a specific catalyst by comparing its *R*_a/d_ value to the optimal value. Moreover, all the values of the optimal *R*_a/d_ in the ACH, 1-HEX and 1-OCT epoxidation are around −13, likely reflecting the mechanistic consistency of epoxidation and the compositional similarity of the reactant.

Besides, the optimal *R*_a/d_ in each reaction can serve as a guide for the modification of the catalysts to enhance their performance. For example, in ACH epoxidation shown in Fig. 5, TS-1-NK presents a high *R*_a/d_ value at −9.8, indicating a deficient diffusion relative to its high intrinsic activity. To address this, NaOH etching is applied as a post-treatment to improve the diffusion of the catalyst. This treatment increases the DDTOF of the obtained catalyst TS-1-NK-Na to about 3 times that of TS-1-NK while the activation energy remains unchanged as displayed in Fig. 3, 4, S46 and S47.† Hence, the enhanced epoxidation is achieved given that the TON increases from about 330 to about 370 as the *R*_a/d_ of TS-1-NK-Na decreases as shown in Fig. 5. A similar modification is performed on TS-1-MKL as shown in Fig. 3, 4, S48 and S49†, resulting in a two-fold increase in DDTOF with the retained activation energy. However, the epoxidation performance of TS-1-MKL-Na in ACH turns out to be worse due to the fact that the *R*_a/d_ of TS-1-MKL is already lower than the optimal *R*_a/d_, so reducing it further by enhanced diffusion only leads to a detrimental effect on the epoxidation performance. In contrast, for catalysts with low *R*_a/d_, such as TS-1-43, increasing its *R*_a/d_ value would benefit its epoxidation performance. Accordingly, hydrothermal recrystallization with ethylamine is employed to refine the Ti site structure in TS-1-43. The activation energy of the modified TS-1-43-EA is greatly reduced with little change in DDTOF as listed in Fig. 3, 4, S50 and S51.† In ACH epoxidation, the *R*_a/d_ value of TS-1-43-EA increases to about −11, and the epoxidation performance is significantly enhanced with a TON value up to around 400.

In 1-HEX epoxidation, however, the *R*_a/d_ value of TS-1-43-EA increases excessively to about −5, resulting in a minor increase in epoxidation performance. For TS-1-NK-Na and TS-1-MKL-Na in 1-HEX peroxidation, the *R*_a/d_ values remain too high despite the decrease in *R*_a/d_ due to the severe diffusion limitation, leading to an insignificant change in epoxidation performance. These results not only indicate that the epoxidation performance can be efficiently optimized by adopting appropriate modification guided by *R*_a/d_, but also reveal the reason behind the efficacy of mesopore construction and active site refinement in previous studies. Among proposed methods improving epoxidation performance, diffusion enhancement^15,66^ and active site structure optimization^21,43^ are emphasized respectively. The former leads to a lower *R*_a/d_ and the latter results in a higher *R*_a/d_ owing to higher intrinsic activity. The complementary nature of these strategies is now clear: improving diffusion is beneficial for catalysts with *R*_a/d_ higher than the optimal one, while enhancing intrinsic activity is advantageous for catalysts with lower *R*_a/d_. In summary, for highly active catalytic systems, further increasing *R*_a/d_ by boosting intrinsic activity is counterproductive, while improving diffusion is helpful. Conversely, for catalysts with low *R*_a/d_, enhancing the intrinsic activity is the key to improving performance. This insight highlights the importance of matching the modification strategy to the catalyst's current *R*_a/d_ in order to optimize its performance for specific reactions.

## 4. Conclusion

In summary, a strategy is proposed for precise modulation of the local microenvironment around active sites in TS-1 by adjusting the contributions of classical and non-classical crystallization. Based on the series of TS-1 catalysts with explicit evolvement of the space-confined structure, hydrophilicity and active Ti site structure, *R*_a/d_ is introduced as a descriptor for evaluation of the collaboration between intrinsic activity and diffusion in alkene epoxidation by measuring catalyst intrinsic activity and epoxide diffusion in advance. The performance of catalysts, including various TS-1 samples and commercial catalysts, in ACH, 1-HEX and 1-OCT epoxidation exhibits volcano-shaped curves when plotted against *R*_a/d_. Optimal *R*_a/d_ values are found to be close to about −13, where the best epoxidation performance across all alkene substrates is observed. The factors hindering epoxidation performance can be identified under the guidance of *R*_a/d_, enabling efficient catalyst optimization. As the optimal *R*_a/d_ refers to the balanced intrinsic activity and diffusion, a higher *R*_a/d_ indicates the exorbitant intrinsic activity compared to diffusion leading to rapid deactivation and a lower *R*_a/d_ demonstrates the insufficient intrinsic activity, both resulting in unideal epoxidation performance. The study proposes a universal descriptor and a corresponding catalyst platform construction strategy for quantitative analysis of TS-1 catalysts including both intrinsic activity and diffusion, which allows the forecast and explanation of the catalyst reaction performance and further sheds light on systemic analysis of the structure–activity relationship of catalysts in heterogeneous catalysis and rational design of zeolite catalysts.

## Data availability

The data supporting this article have been included as part of the ESI.†

## Author contributions

Conceptualization: Di Pan; methodology: Di Pan; investigation: Di Pan; writing – original draft: Di Pan; writing – review & editing: Di Pan, Jiayu Yu, Ke Du, Kexin Yan, Ling Ding, Yahong Zhang and Yi Tang; funding acquisition: Yahong Zhang and Yi Tang; resources: Yahong Zhang and Yi Tang; supervision: Yahong Zhang and Yi Tang.

## Conflicts of interest

There are no conflicts to declare.

## Supplementary Material

SC-016-D5SC00987A-s001
