# Supplementary material for: Evaluation of the collaboration between intrinsic activity and diffusion: a descriptor for alkene epoxidation catalyzed by TS-1
Source: Chem Sci. 2025 Mar 1;16(14):5931–41. doi: 10.1039/d5sc00987a (PMC11884413; doi:10.1039/d5sc00987a)
Supplement: SC-016-D5SC00987A-s001 [file SC-016-D5SC00987A-s001.pdf]

Supplementary Information (SI) for Chemical Science.
This journal is © The Royal Society of Chemistry 2025

Supporting Information (SI)

Evaluation of Collaboration between Intrinsic Activity and Diffusion: a Descriptor for Alkene Epoxidation Catalyzed by TS-1

Di Pan, Jiayu Yu, Ke Du, Kexin Yan, Ling Ding, Yahong Zhang* and Yi Tang*

* Department of Chemistry, Shanghai Key Laboratory of Molecular Catalysis and Innovative Materials, Laboratory of Advanced Materi-als, Collaborative Innovation Centre of Chemistry for Energy Materials (iChEM)

Fudan University

Shanghai 200433, P. R. China

Yahong Zhang E-mail: zhangyh@fudan.edu.cn

Yi Tang E-mail: yitang@fudan.edu.cn

Synthesis of TS-1 initial synthetic sol 5

Synthesis of TS-1 SCs sol 5

Measurement of liquid phase breakthrough curves 5-6

Diffusion calculation of dynamics in sorption isotherms 7

Figure S1. DLS size-time crystallization plot of TS-1 subcrystal synthesis 8

Figure S2. HAXRD profiles of TS-1-43 with Ti located at different T sites 9-10

Figure S3. HAXRD profiles of TS-1-71 with Ti located at different T sites 11-12

Figure S4. HAXRD profiles of TS-1-93 with Ti located at different T sites 13-14

Figure S5. HAXRD profiles of TS-1-100 with Ti located at different T sites 15-16

Figure S6. Ar adsorption isotherms of TS-1 samples 17

Figure S7. SEM images of of TS-1 samples 18

Figure S8. TG and DSC plots of TS-1 samples 19

Figure S9. Experimental and simulated Ar adsorption isotherms and assumed strictures 20-21

Figure S10. Detailed Ar adsorption isotherms simulation based on Z5-C4 22-23

Figure S11. Simulated Ar adsorption isotherms of Z5-C4 structure with different cell parameters 24

Figure S12. Adsorption isotherms of methanol of TS-1 samples 25

Figure S13. Peak fittings of ^{1}H MAS SSNMR spectra 26

Figure S14. Peak fittings of UV-vis spectra 27

Figure S15. XAFS and WT-EXAFS plots of TS-1-93 28

Figure S16. Reaction process of alkene epoxidation in TS-1-43 29

Figure S17. Reaction process of alkene epoxidation in TS-1-71 30

Figure S18. Reaction process of alkene epoxidation in TS-1-93 31

Figure S19. Reaction process of alkene epoxidation in TS-1-100 32

Figure S20. Isotherms and kinetic uptake curves of alkene in TS-1-43 33-34

Figure S21. Isotherms and kinetic uptake curves of alkene in TS-1-71 35-36

Figure S22. Isotherms and kinetic uptake curves of alkene in TS-1-93 37-38

Figure S23. Isotherms and kinetic uptake curves of alkene in TS-1-100 39-40

Figure S24. Isotherms and kinetic uptake curves of epoxide in TS-1-43 41-42

Figure S25. Isotherms and kinetic uptake curves of epoxide in TS-1-71 43-44

Figure S26. Isotherms and kinetic uptake curves of epoxide in TS-1-93 45-46

Figure S27. Isotherms and kinetic uptake curves of epoxide in TS-1-100 47-49

Figure S28. DDTOF of alkene in gas phase 50

Figure S29. The breakthrough curve and fitting profile in empty tube 51

Figure S30. The breakthrough curves with trimethylbenzene eluent 52

Figure S31. The breakthrough curves with 0.2 wt.% methanol trimethylbenzene eluent..53

Figure S32. The breakthrough curves with 2 wt.% methanol trimethylbenzene eluent 54

Figure S33. DDTOF of alkene in liquid phase 55

Figure S34. $R_{a/d}$ values of TS-1 samples...56

Figure S35. Epoxide selectivity in alkene epoxidation..57

Figure S36. Reaction process of alkene epoxidation in TS-1-NK.............................58

Figure S37. Isotherms and kinetic uptake curves of epoxide in TS-1-NK 59-60

Figure S38. Reaction process of alkene epoxidation in TS-1-MKL..........................61

Figure S39. Isotherms and kinetic uptake curves of epoxide in TS-1-MKL 62-63

Figure S40. Reaction process of alkene epoxidation in TS-1-RD 64

Figure S41. Isotherms and kinetic uptake curves of epoxide in TS-1-RD 65

Figure S42. Reaction process of alkene epoxidation in TS-1-YK..............................66

Figure S43. Isotherms and kinetic uptake curves of epoxide in TS-1-YK 67

Figure S44. Reaction process of alkene epoxidation in TS-1-65..........................68-69

Figure S45. Isotherms and kinetic uptake curves of epoxide in TS-1-65 70-71

Figure S46. Reaction process of alkene epoxidation in TS-1-NK-Na........................72

Figure S47. Isotherms and kinetic uptake curves of epoxide in TS-1-NK-Na 73-74

Figure S48. Reaction process of alkene epoxidation in TS-1-MKL-Na.....................75

Figure S49. Isotherms and kinetic uptake curves of epoxide in TS-1-MKL-Na ...76-77

Figure S50. Reaction process of alkene epoxidation in TS-1-43-EA 78

Figure S51. Isotherms and kinetic uptake curves of epoxide in TS-1-43-EA 79-80

Table S1. The initial non-classical contribution in sample synthesis 81

Table S2. Calculated cell parameters of TS-1 samples 82

Table S3. The estimated Ti site proportion in TS-1 samples 83

Table S4. $\frac{\alpha}{l}$ values for different adsorbates in TS-1 samples 84

References 85

Synthesis of TS-1 initial synthetic sol

The TS-1 initial synthetic sol was hydrothermally synthesized by using sillica sol as the silicon source, titanic sulfate as the titanium source, and tetrapropylammonium hydroxide (TPAOH) as the structure-directing agent in an Anton Paar monowave 300 microwave synthesizer. The synthetic sol composition corresponded to the formula 40 SiO_2: 1.0 TiO_2: 15.17 TPAOH: 720 H_2O. The TS-1 initial synthetic sol was collected just at the inflection point of the size-time crystallization curve (Figure S1). After ageing at room temperature for 18 h, the clear sol was transferred into 30 mL explosion-proof glass tube and pretreated at 90 °C for 90 min under microwave irradiation. Immediately, the obtained clear liquid was heated at 140 °C for 130 min, followed by centrifugation. After removing the minor sediment, the supernatant was collected and named as TS-1 initial synthetic sol.

Synthesis of TS-1 SCs sol

The TS-1 SC sol was separated and purified from TS-1 initial synthetic sol by two-step dialysis procedure. First the synthesis liquor was performed dialysis once while 10 mmol/L TPAOH solution acted as outer liquid, and then TPAOH solution was replaced by deionized (DI) water for further dialysis in the following three times. Each dialysis lasted for 24 hours to ensure complete material balance. After dialysis, the pH value of obtained transparent sol was around 9, and then the solid content of TS-1 precursor particles sol was adjusted to 50 mg/g sol and named as TS-1 SC sol.

Measurement of liquid phase breakthrough curves

The liquid phase breakthrough curves were recorded at 303K by a handmade instrument as the following scheme shown. For a typical run, 40 mg sample was loaded into the stainless steels tube with cotton wool loaded in advance. After making samples stocky, another piece of cotton wool was loaded on the sample into the tube. Under 303 K water bath, the sample was first flushed by 0.2 wt.% 1-hexene 1,3,5-trimethylbenzene solution at 0.2 mL/min for 1 h for saturation with 1-hexene. Then the eluent was shifted

by switching the input fluid. The eluents include pure 1,3,5-trimethylbenzene, 0.2 wt.% methanol 1,3,5-trimethylbenzene solution and 2 wt.% methanol 1,3,5-trimethylbenzene solution. The effluent was collected over time and the concentration of 1-hexene was then measured on a gas chromatograph (GC-2010 Plus), equipped with a 30 m capillary column (DB-WAX) and an FID detector by external standard. The background curve was recorded with empty tube and was fitted by sigma function. The theorical diffusion turnover frequencies (DDTOFs) of 1-hexene during desorption obtained from liquid phase breakthrough curves were calculated by following formulars.

$$\boldsymbol{DDTOF} = \frac{\boldsymbol{Q} \times \boldsymbol{C_{adsorbate}} \times \boldsymbol{M_{Ti}}}{\boldsymbol{m_{adsorbent}} \times \boldsymbol{W_{Ti}}}$$

in which Q is the flow rate of eluents. $C_{adsorbate}$ is the concentration of adsorbate, here referring 1-hexene. M_{Ti} is the relative atomic weight of Ti. $m_{adsorbent}$ is the mass of loaded adsorbent. W_{Ti} is the Ti weight percentage of adsorbent.

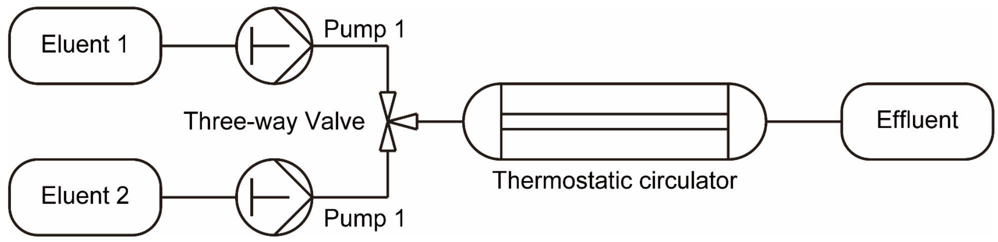

Scheme 1. The instrument measuring liquid phase breakthrough curves. When recording breakthrough curves of 1-hexene, the eluent 1 is 0.2 wt.% 1-hexene 1,3,5-trimethylbenzene solution, and the eluent 2 is include pure 1,3,5-trimethylbenzene, 0.2 wt.% methanol 1,3,5-trimethylbenzene solution and 2 wt.% methanol 1,3,5-trimethylbenzene solution. The samples were loaded in the tube inside the thermostatic circulator which is kept at 303 K. The flowing rate was 0.2 mL/min in both pumps. The effluent was collected by drops and then analyzed.

Diffusion calculation of dynamics in sorption isotherms

The sorption isotherms and corresponding dynamics data of water, methanol, 1-hexene, 1,2-epoxyhexane, allyl chloride, epichlorohydrin, 1-octene and 1,2-epoxyoctane were collected by an IGA100B. The fitting of uptake curves at the initial stage by surface barrier model (SBM) and the theorical diffusion turnover frequency (DDTOF) during desorption obtained from desorption isotherms were based on following formulars respectively.

$$\frac{\boldsymbol{q(t)}}{\boldsymbol{q(m)_{ini}}} = \frac{\boldsymbol{\alpha}}{\boldsymbol{l}} \times (\sqrt{\boldsymbol{t}})^2 + \boldsymbol{O}(\sqrt{\boldsymbol{t}})^3$$

$$\boldsymbol{DDTOF} = \frac{\boldsymbol{\alpha}}{\boldsymbol{l}} \times \frac{\Delta \boldsymbol{m} \times \boldsymbol{M_{Ti}}}{\boldsymbol{M_{adsorbate}} \times \boldsymbol{W_{Ti}}}$$

in which t is the sorption time. α is the surface permeability. l is the diffusion path length. Δm is the percentage of mass change during adsorption or desorption. M_{Ti} is the relative atomic mass of Ti. $M_{adsorbate}$ is the relative mass of adsorbate. W_{Ti} is the Ti weight percentage of adsorbent. The $\boldsymbol{O}(\sqrt{\boldsymbol{t}})^3$ is the ignorable high order term at the early stage of sorption following SBM.

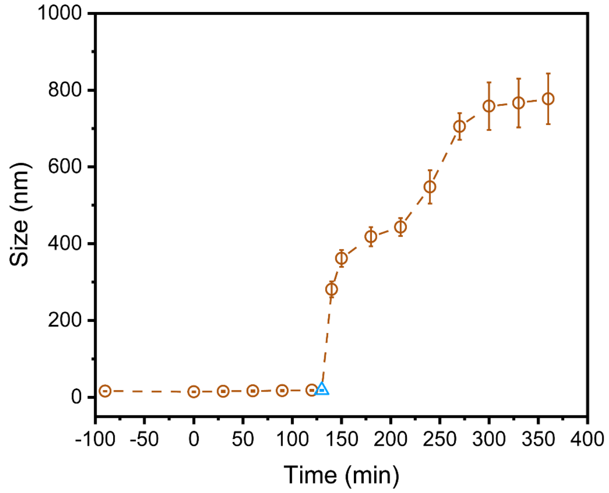

Figure S1. DLS size-time crystallization plot of TS-1 subcrystal synthesis at 140 °C. The negative values on the time axis refer to the low-temperature treatment at 90 °C before hydrothermal treatments at 140 °C. The triangle sign indicates the time point when the subcrystals are extracted.

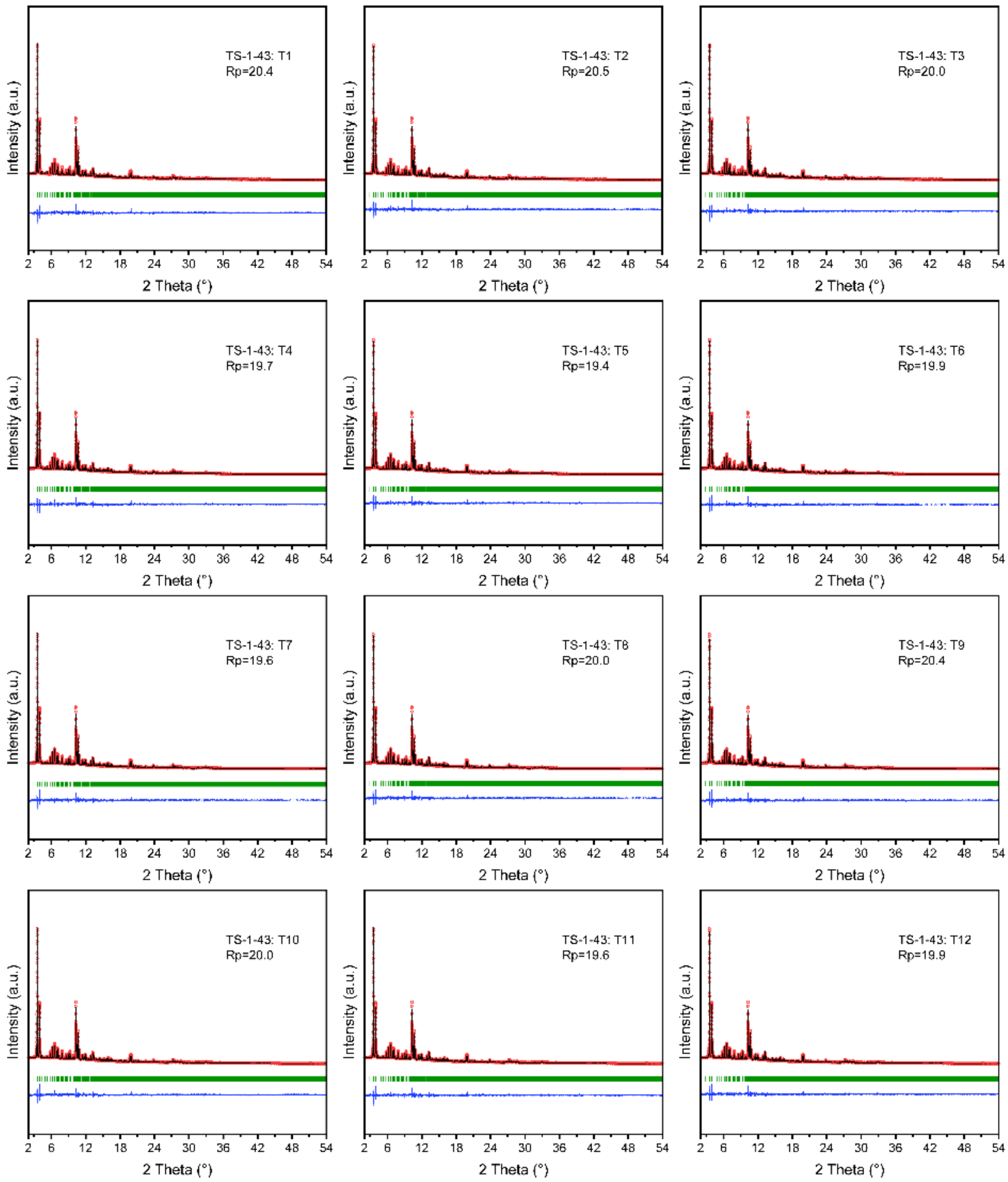

TS-1-43: T1
Rp=20.4
TS-1-43: T2
Rp=20.5
TS-1-43: T3
Rp=20.0
TS-1-43: T4
Rp=19.7
TS-1-43: T5
Rp=19.4
TS-1-43: T6
Rp=19.9
TS-1-43: T7
Rp=19.6
TS-1-43: T8
Rp=20.0
TS-1-43: T9
Rp=20.4
TS-1-43: T10
Rp=20.0
TS-1-43: T11
Rp=19.6
TS-1-43: T12
Rp=19.9
Intensity (a.u.)
2 Theta (°)
2 6 12 18 24 30 36 42 48 54

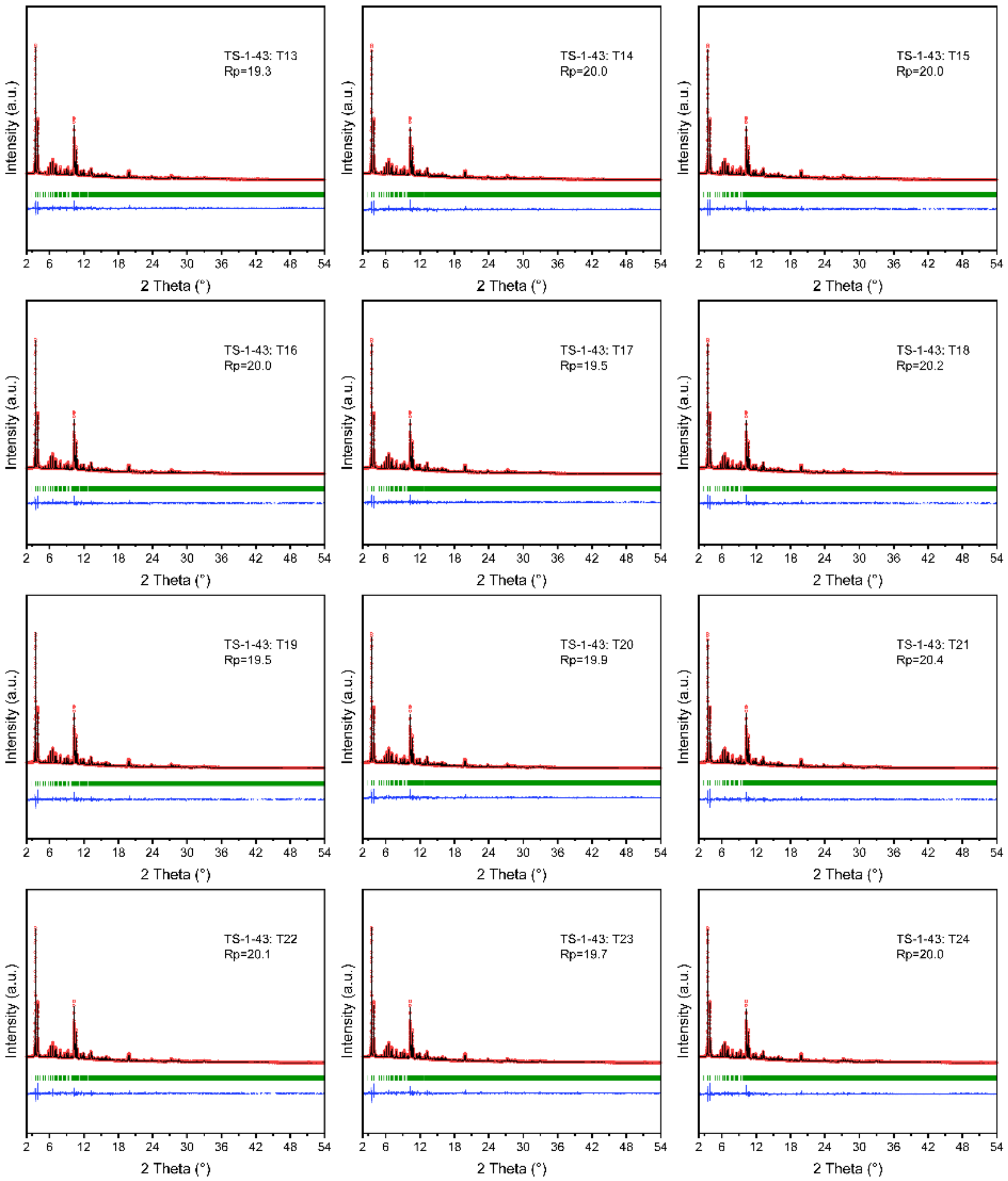

Figure S2. XRD profiles of TS-1-43 with Ti located at different T sites. The solid line and circles are the Rietveld refinements for the lattice and experimental data, respectively. The vertical bars represent the peak positions, and the solid lines at the bottom indicate the residual.

Refinements about Ti location demonstrate a random Ti location in TS-1-43.

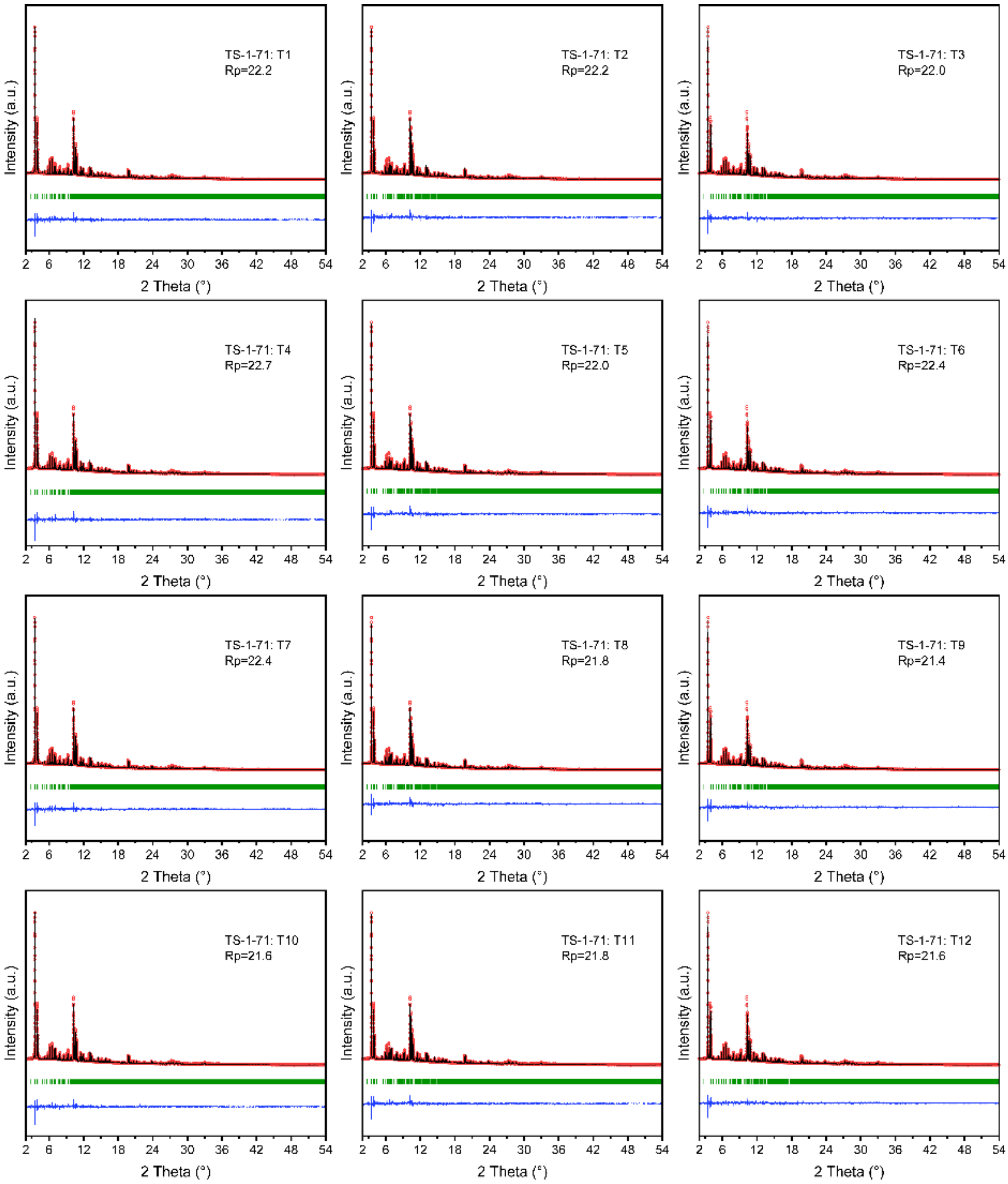
Intensity (a.u.)
2 Theta (°)
TS-1-71: T1
Rp=22.2
TS-1-71: T2
Rp=22.2
TS-1-71: T3
Rp=22.0
TS-1-71: T4
Rp=22.7
TS-1-71: T5
Rp=22.0
TS-1-71: T6
Rp=22.4
TS-1-71: T7
Rp=22.4
TS-1-71: T8
Rp=21.8
TS-1-71: T9
Rp=21.4
TS-1-71: T10
Rp=21.6
TS-1-71: T11
Rp=21.8
TS-1-71: T12
Rp=21.6

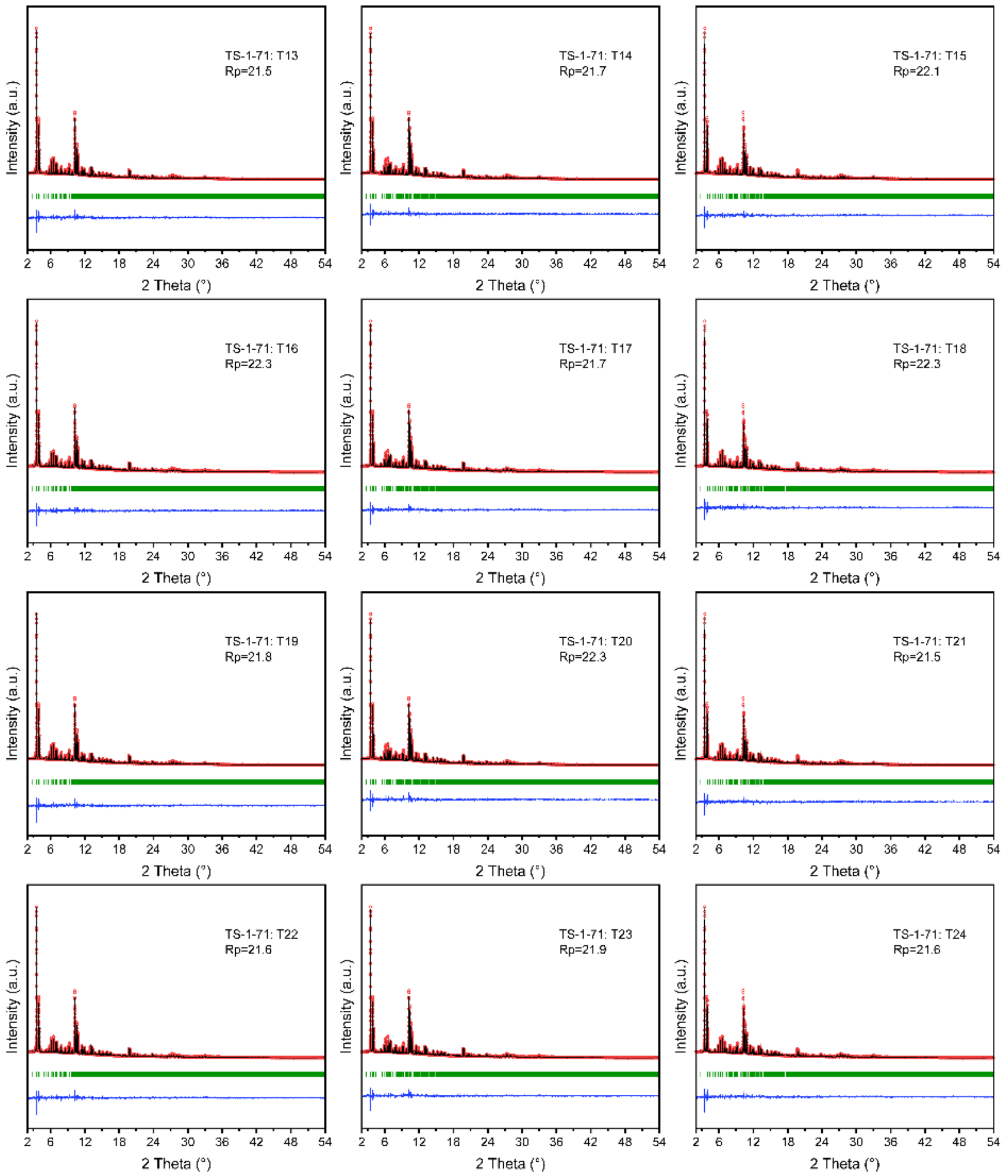

Figure S3. HAXRD profiles of TS-1-71 with Ti located at different T sites. The solid line and circles are the Rietveld refinements for the lattice and experimental data, respectively. The vertical bars represent the peak positions, and the solid lines at the bottom indicate the residual.

Refinements about Ti location demonstrate a random Ti location in TS-1-71.

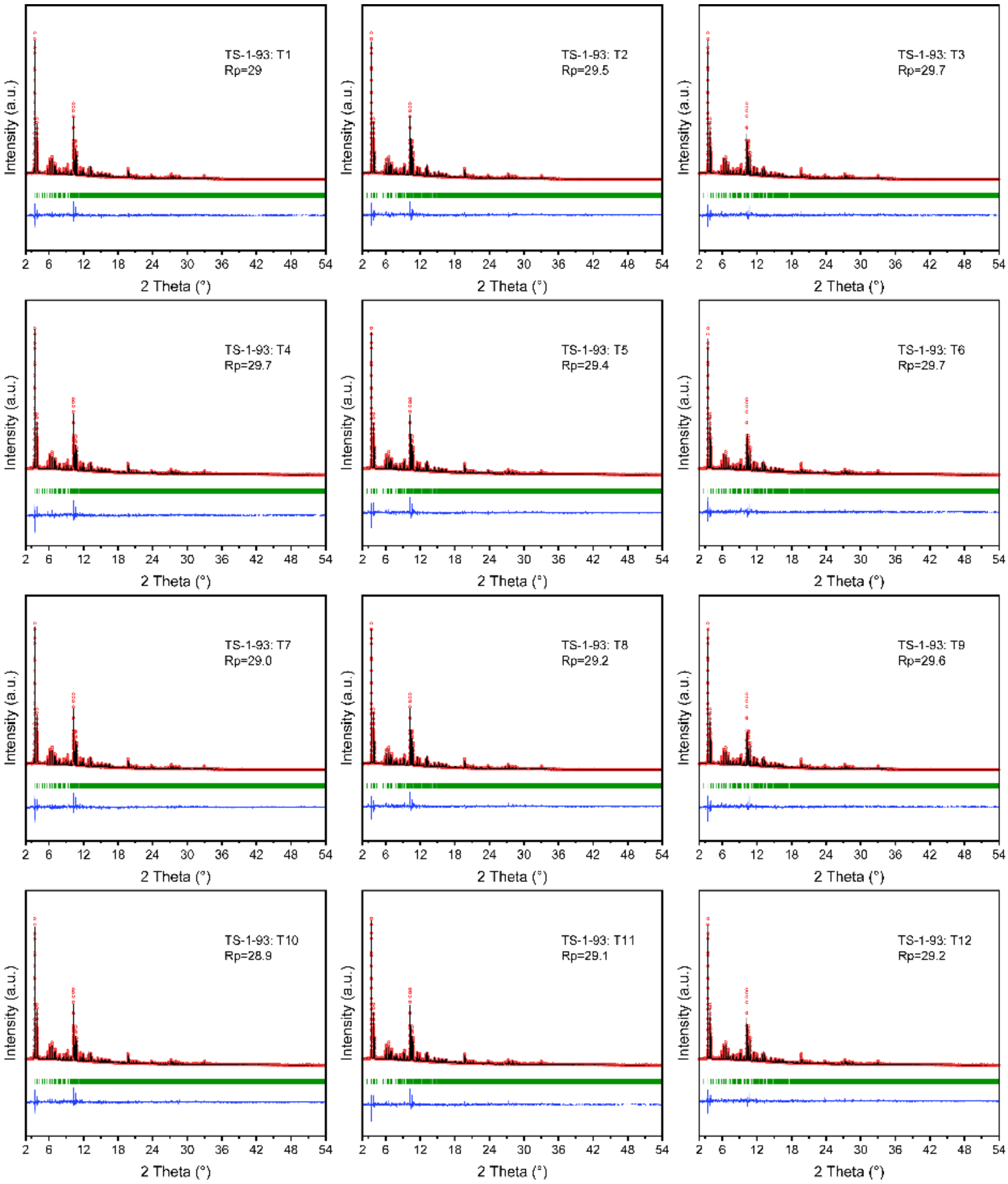
TS-1-93: T1
Rp=29
TS-1-93: T2
Rp=29.5
TS-1-93: T3
Rp=29.7
TS-1-93: T4
Rp=29.7
TS-1-93: T5
Rp=29.4
TS-1-93: T6
Rp=29.7
TS-1-93: T7
Rp=29.0
TS-1-93: T8
Rp=29.2
TS-1-93: T9
Rp=29.6
TS-1-93: T10
Rp=28.9
TS-1-93: T11
Rp=29.1
TS-1-93: T12
Rp=29.2
Intensity (a.u.)
2 Theta (°)
2 6 12 18 24 30 36 42 48 54

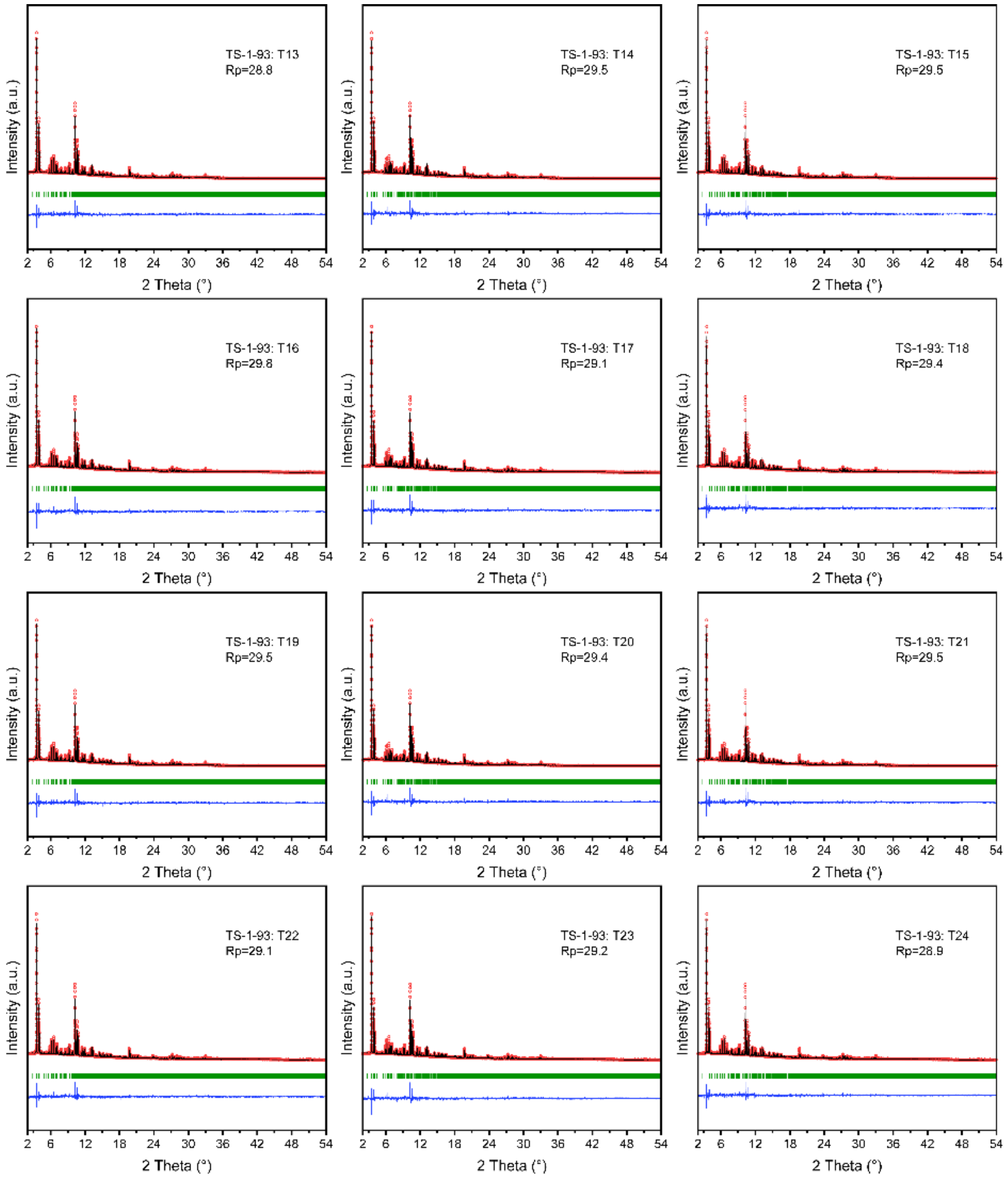

Figure S4. HAXRD profiles of TS-1-93 with Ti located at different T sites. The solid line and circles are the Rietveld refinements for the lattice and experimental data, respectively. The vertical bars represent the peak positions, and the solid lines at the bottom indicate the residual.

Refinements about Ti location demonstrate a random Ti location in TS-1-93.

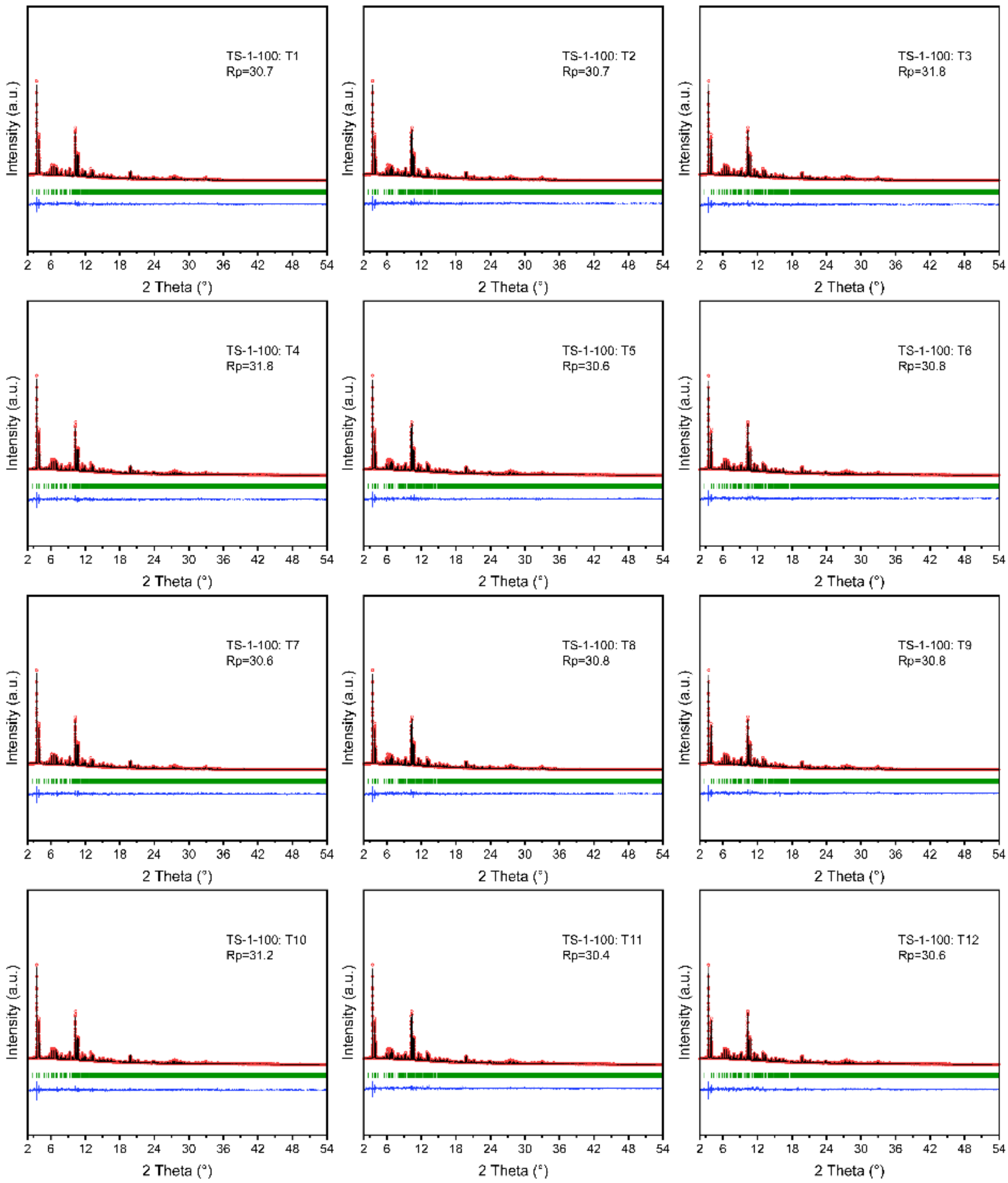

TS-1-100: T1
Rp=30.7
TS-1-100: T2
Rp=30.7
TS-1-100: T3
Rp=31.8
TS-1-100: T4
Rp=31.8
TS-1-100: T5
Rp=30.6
TS-1-100: T6
Rp=30.8
TS-1-100: T7
Rp=30.6
TS-1-100: T8
Rp=30.8
TS-1-100: T9
Rp=30.8
TS-1-100: T10
Rp=31.2
TS-1-100: T11
Rp=30.4
TS-1-100: T12
Rp=30.6
Intensity (a.u.)
2 Theta (°)
2
6
12
18
24
30
36
42
48
54

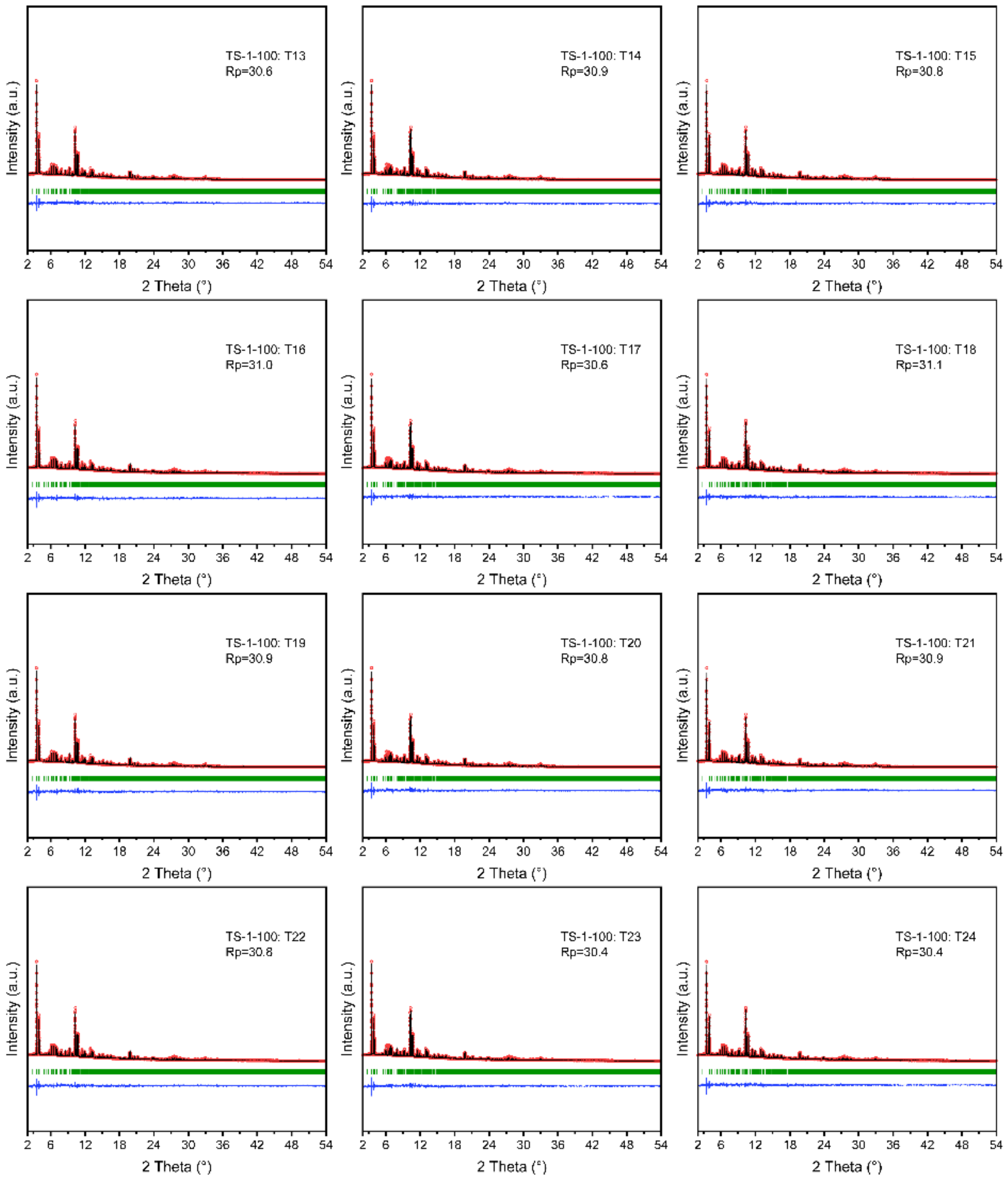

Figure S5. HAXRD profiles of TS-1-100 with Ti located at different T sites. The solid line and circles are the Rietveld refinements for the lattice and experimental data, respectively. The vertical bars represent the peak positions, and the solid lines at the bottom indicate the residual.

Refinements about Ti location demonstrate a random Ti location in TS-1-100.

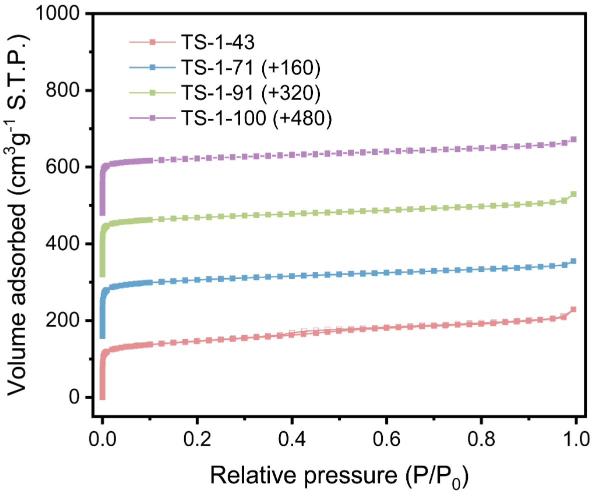

Figure S6. Ar adsorption-desorption isotherms of TS-1 samples.

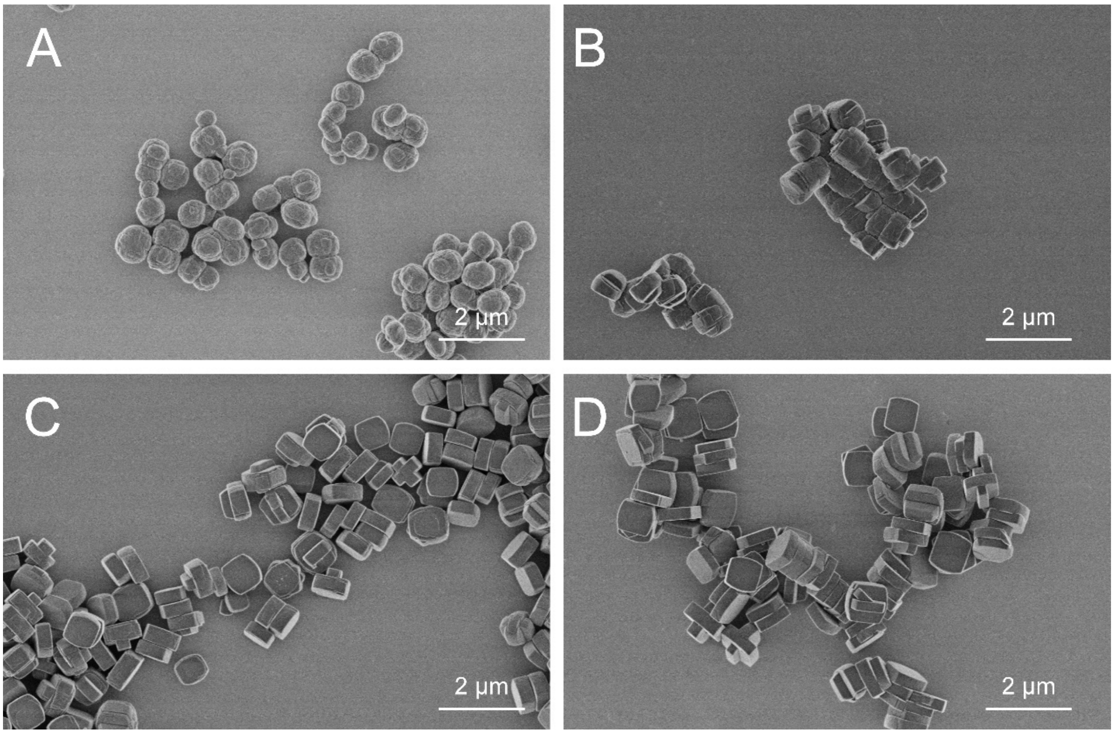

Figure S7. SEM images of (A) TS-1-43, (B) TS-1-71, (C) TS-1-93 and (D) TS-1-100.

Scanning electron microscope (SEM) images show that all the samples are micron-sized particles. TS-1-43 is spherical-like particles with tough surface, while TS-1-93 and TS-1-100 are well-defined twin-shaped and coffin crystals. TS-1-71 also exhibits twin-shaped and coffin morphology, but large aggregations composed by crystals are observed instead of dispersed crystals.

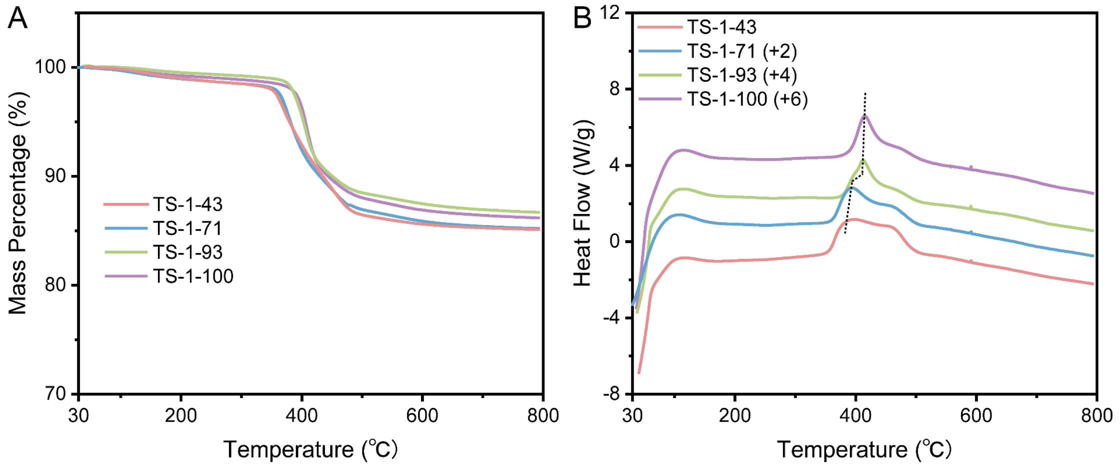

Figure S8. TG plots (A) and DSC plots (B) of TS-1 samples.

Two pyrolysis temperature peaks are observed in DSC plots. The peak at lower temperatures moves to higher temperature with more contribution from non-classical contribution, indicating the harder template pyrolysis which is attributed to the contraction of crossed orifices from 0.87 to 0.72 nm. The peak at higher temperatures locates at the same place, resulting from the enhanced template pyrolysis difficulty at the narrow orifices with the width of 0.64 nm existing in all the samples.

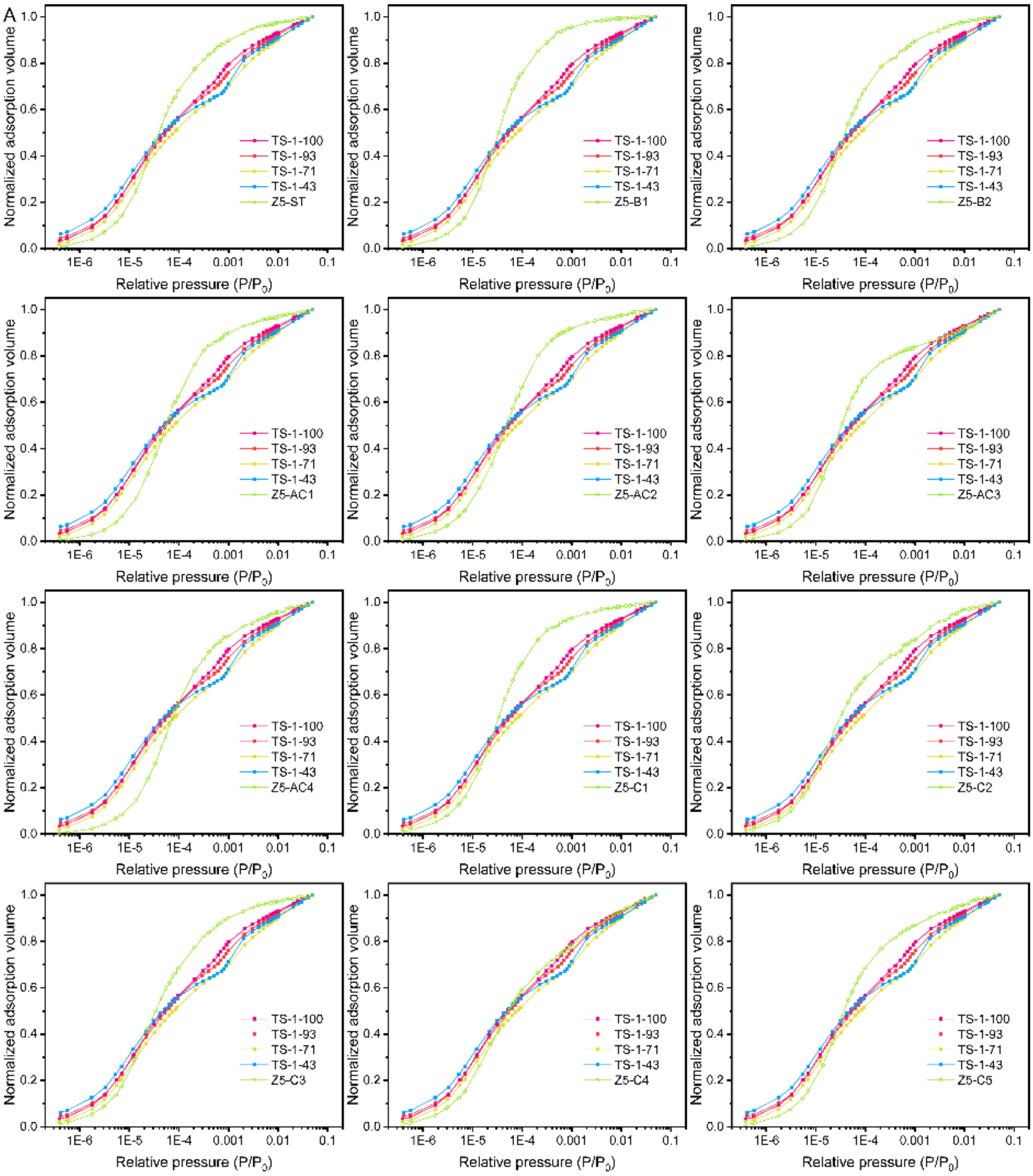
A
Normalized adsorption volume
Relative pressure (P/P0)
0.0
0.2
0.4
0.6
0.8
1.0
1E-6
1E-5
1E-4
0.001
0.01
0.1
TS-1-100
TS-1-93
TS-1-71
TS-1-43
Z5-ST
Z5-B1
Z5-B2
Z5-AC1
Z5-AC2
Z5-AC3
Z5-AC4
Z5-C1
Z5-C2
Z5-C3
Z5-C4
Z5-C5

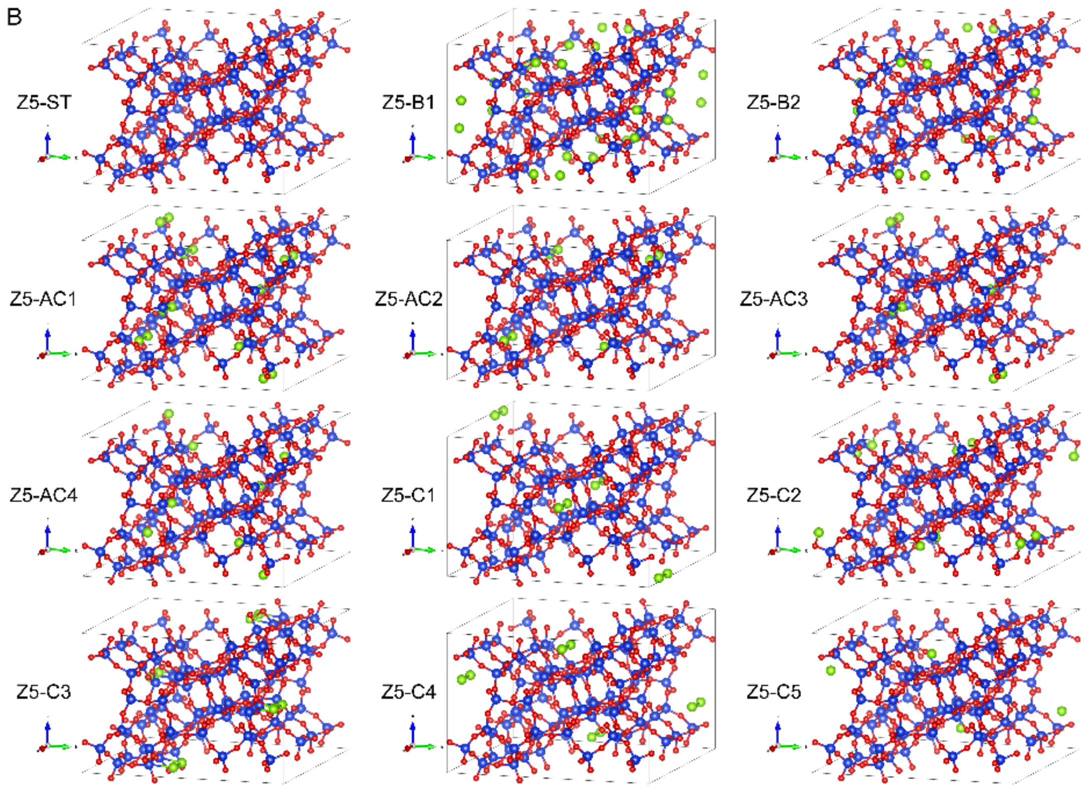

Figure S9. Ar adsorption isotherms of TS-1 samples and simulated Ar adsorption isotherms (A) and assumed strictures (B). The red spheres, blue spheres and green spheres refer to O atoms, Si atoms and barriers with the size of O atoms, respectively.

To certify the specific forms of the contraction of space-confined structures, argon adsorption isotherm simulations of microstructures with various space clogs are carried out. Although the accuracy of simulated absolute adsorption volume is limited owing to the ignorance of mesopore, external surface and defects, information about pore structures is still revealed by the comparation between normalized adsorption isotherms. The simulations indicate that the experimental isotherms could be matched only when the outer side of the sinusoidal tunnel of the cross orifice is partly blocked.

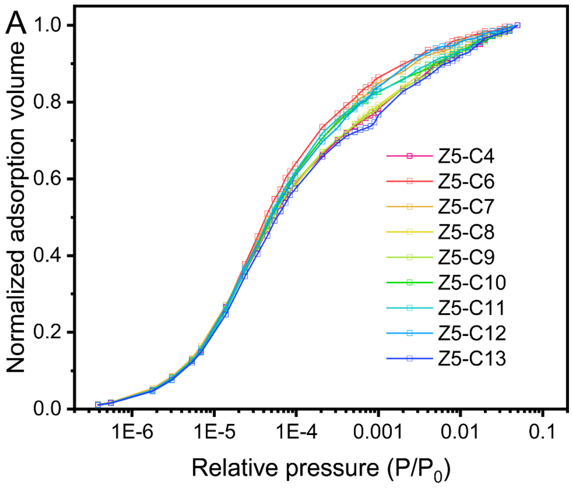

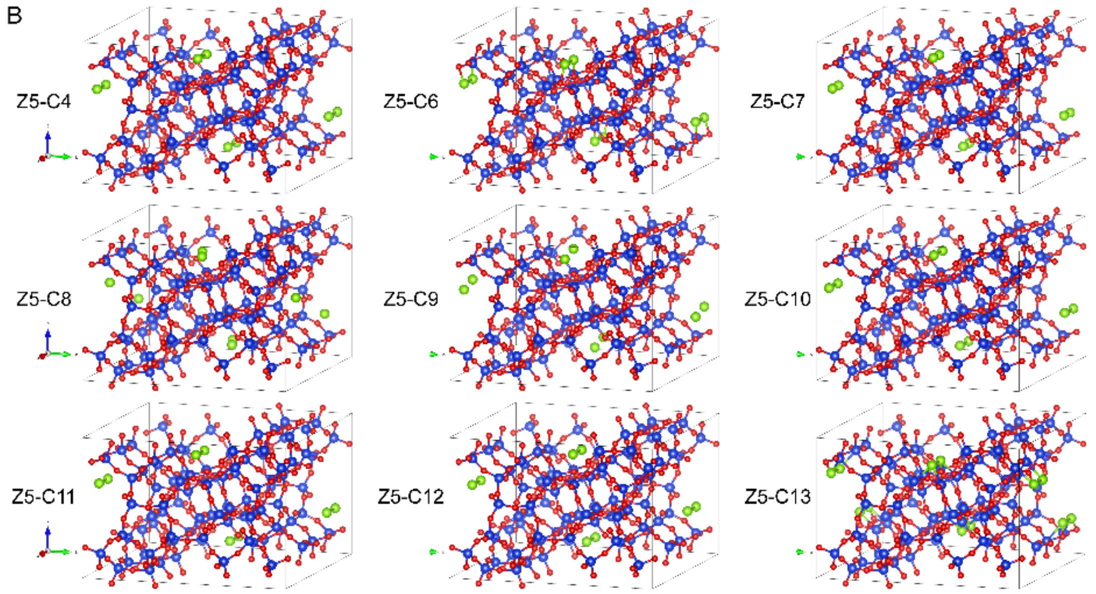

Figure S10. Detailed Ar adsorption isotherms simulation (A) and assumed strictures (B). The red spheres, blue spheres and green spheres refer to O atoms, Si atoms and barriers with the size of O atoms, respectively.

The barriers are moved in different dimensions. Originating from Z5-C4, barriers in Z5-C6 and Z5-C13 move reversely along c axis, in Z5-C7 move for shorter distance along a axis., in Z5-C8 and Z5-C9 rotate around c and b axis respectively, in Z5-C10 and Z5-C11 move reversely and forward a axis respectively, in Z5-C-12 move forward b axis.

Simulations about detailed positions of barriers figure out that tendency of changes in isotherms from T-S-1-100 to TS-1-43 is consist with simulated isotherms with

barriers moving closer the pore walls at the structure mentioned above, agreeing with the contraction of microstructure found from pore size distributions.

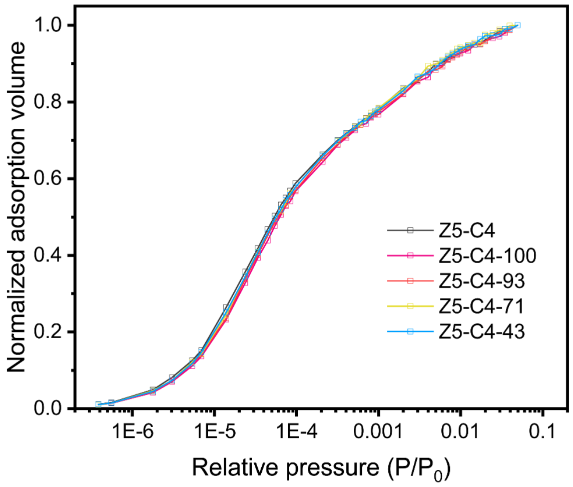

Figure S11. Simulated Ar adsorption isotherms of Z5-C4 structure with the cell parameters of traditional ZSM-5, TS-1-100, TS-1-93, TS-1-71 and TS-1-43, named as Z5-C4, Z5-C4-100, Z5-C4-93, Z5-C4-71 and Z5-C4-43.

After simulations with Z5-C4 structure with different cell parameters, the cell distortions are proofed independent from changes in argon adsorption isotherms.

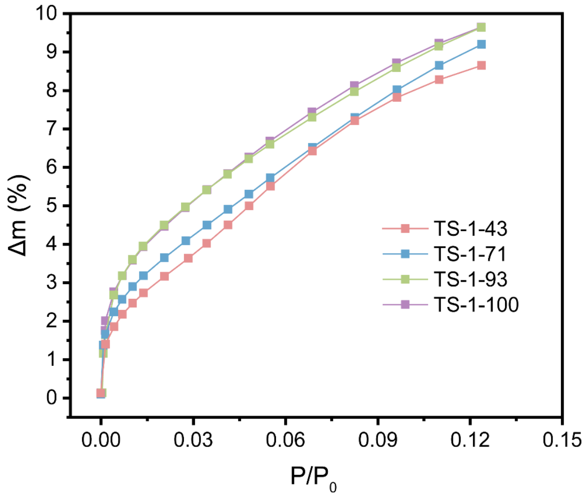

Figure S12. Adsorption isotherms of methanol of TS-1 samples.

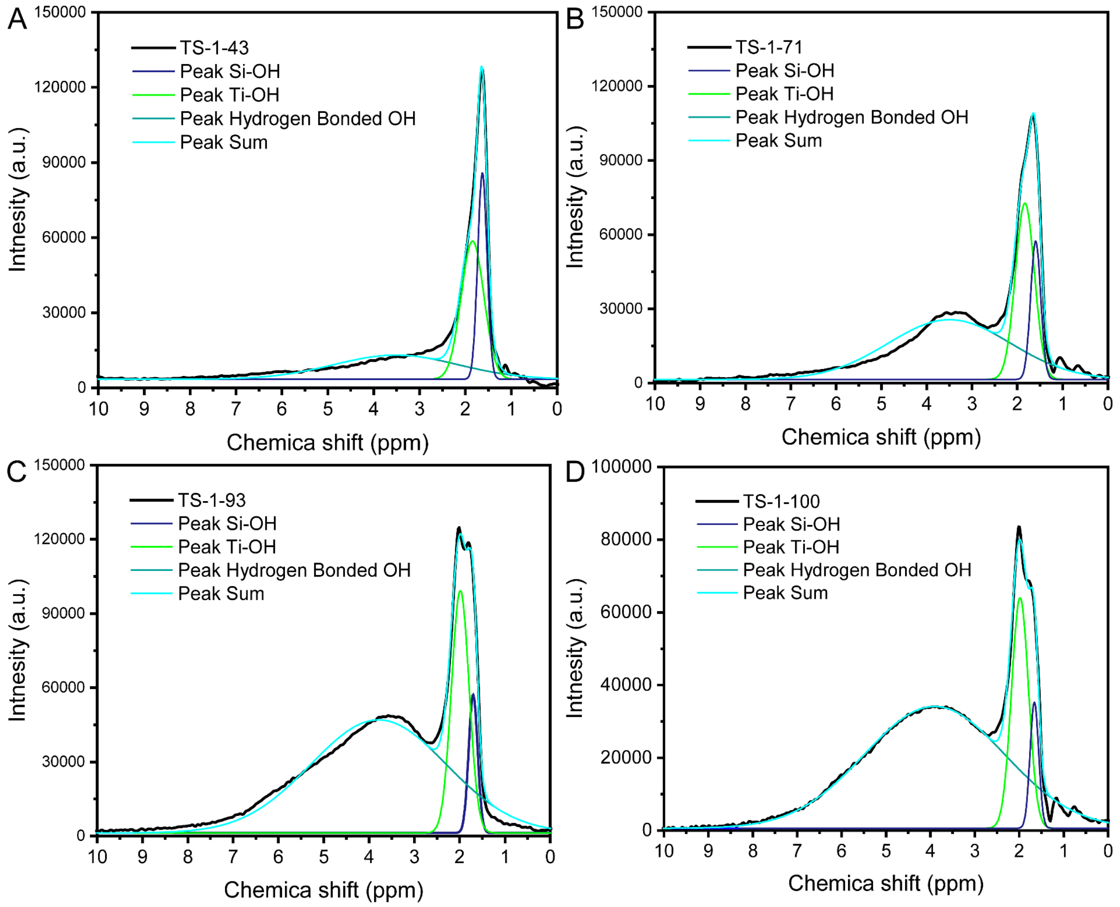

Figure S13. Peak fittings of ^1H SS MAS NMR spectra of (A) TS-1-43, (B) TS-1-71, (C) TS-1-93 and (D) TS-1-100. The calculation is based on Gaussian fitting method.

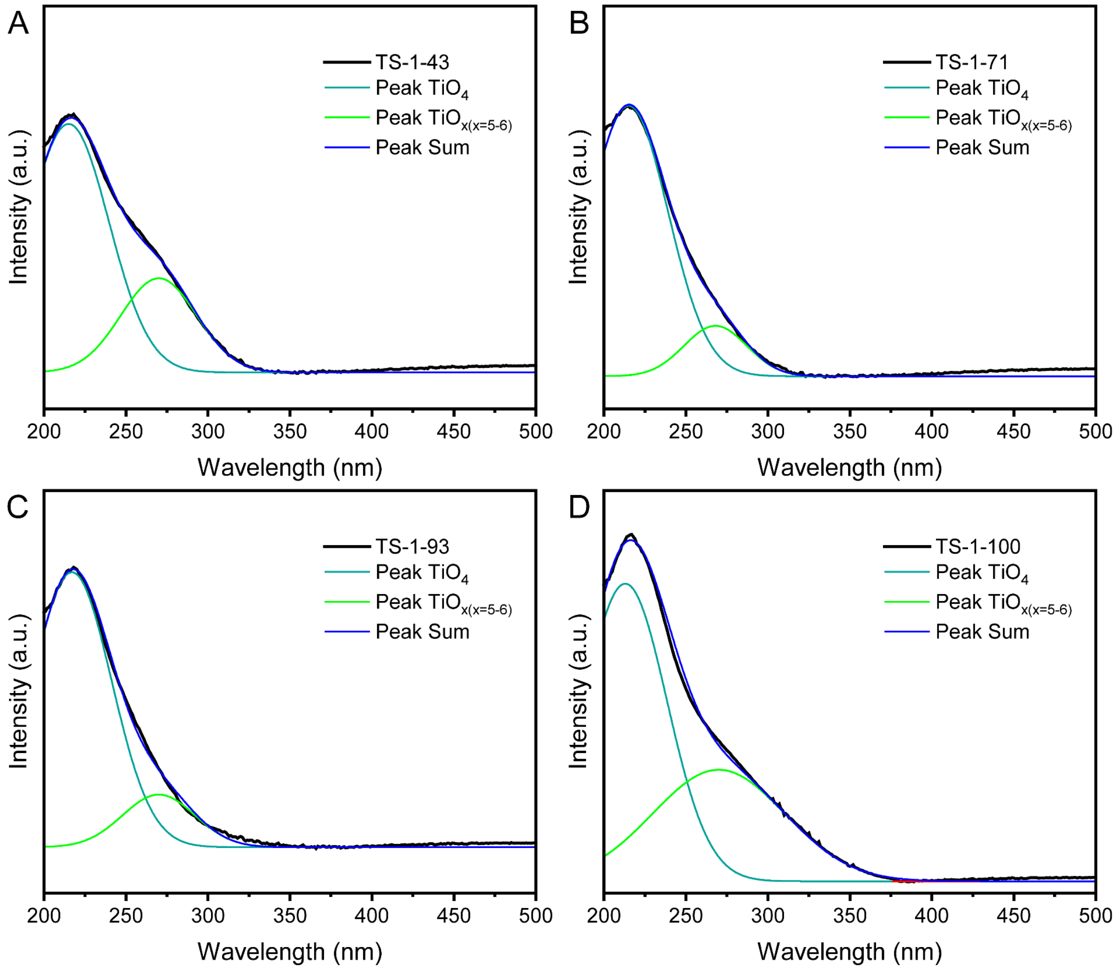

Figure S14. Peak fittings of UV-vis spectra of (A) TS-1-43, (B) TS-1-71, (C) TS-1-93 and (D) TS-1-100. The calculation is based on Gaussian fitting method.

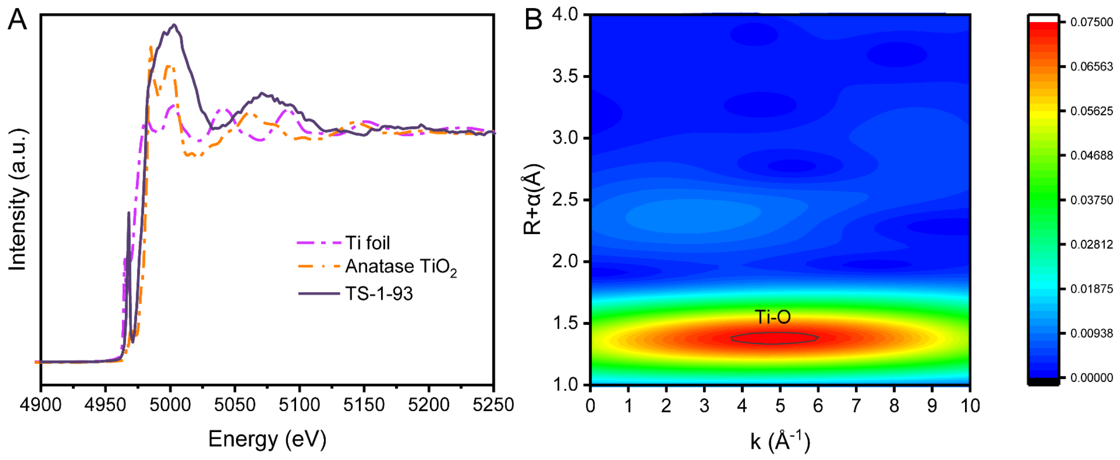

Figure S15. XAFS plots (A) and WT-EXAFS plots (B) of TS-1-93.

X-ray adsorption fine structure (XAFS) tests are performed on TS-1-93 to further reveal the local structure around Ti sites. The emergence of [TiO_5] is proved to be concomitant with local Ti-Ti structure in previous works [1]. Wavelet-transform extended x-ray absorption fine structure (WT-EXAFS) spectrum reveals the absence of local Ti-Ti structure with short distance between Ti atoms, which confirms [TiO_5] could hardly be constructed unless non-classical crystallization fully dominates crystallization.

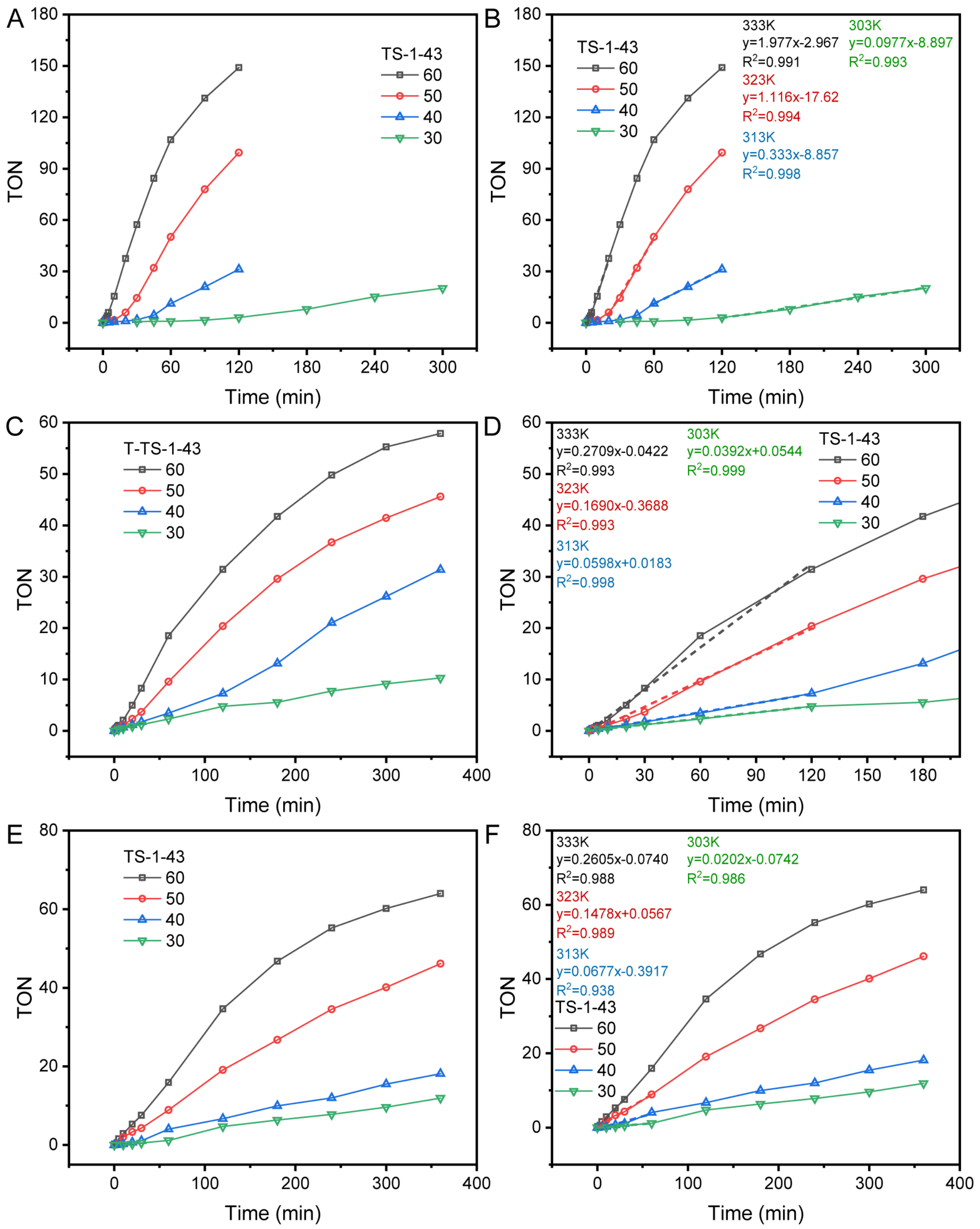

Figure S16. Reaction process (A) and pseudo-first order reaction kinetics fittings (B) of allyl chloride epoxidation, reaction process (C) and pseudo-first order reaction kinetics fittings (D) of 1-hexene epoxidation, reaction process (E) and pseudo-first order reaction kinetics fittings (F) of 1-octene epoxidation in TS-1-43. Reaction conditions: catalyst 50 mg, olefin 10 mmol, H_2O_2 10 mmol, methanol 10 mL. The temperatures change from 303 to 333 K at 10 K interval.

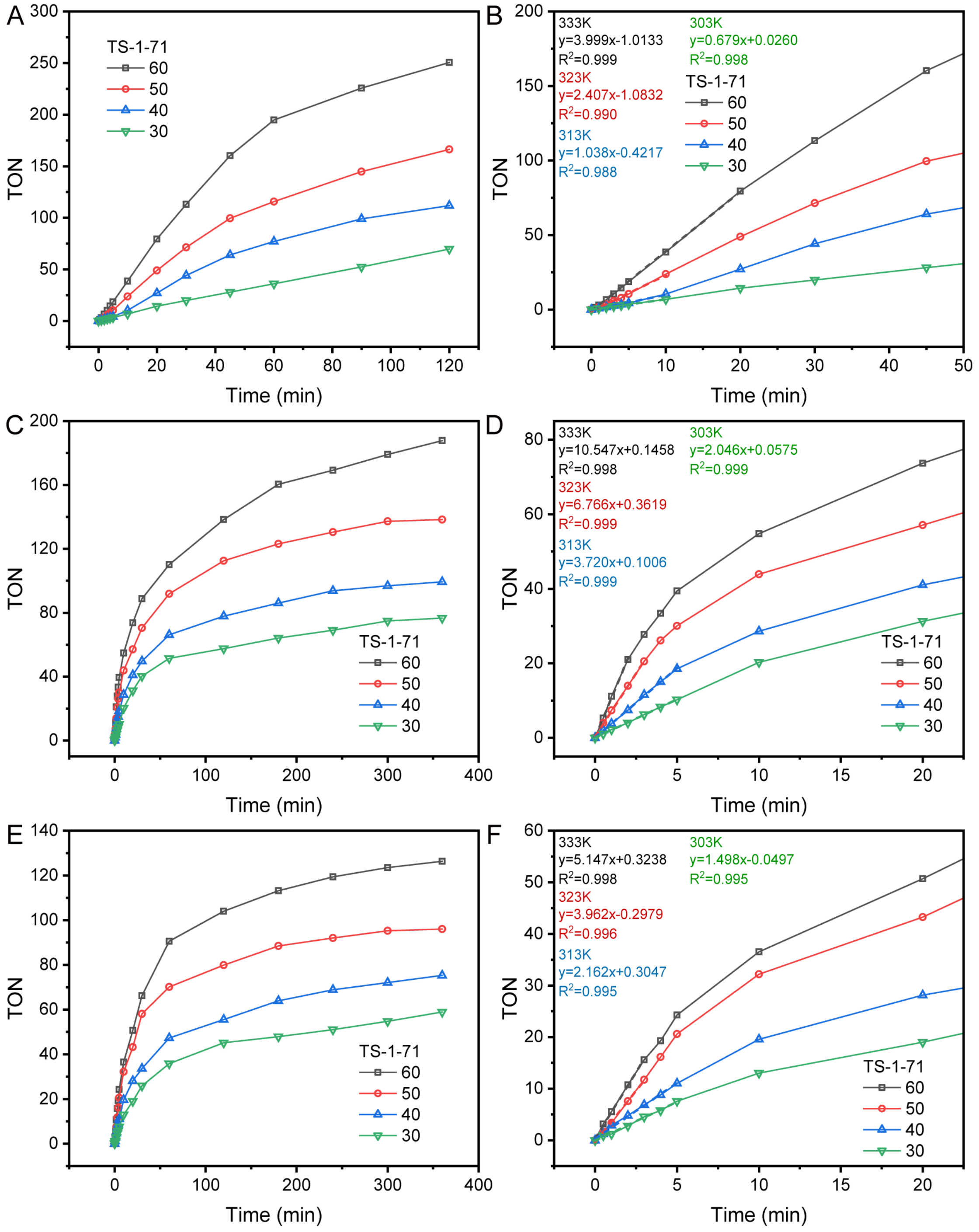

Figure S17. Reaction process (A) and pseudo-first order reaction kinetics fittings (B) of allyl chloride epoxidation, reaction process (C) and pseudo-first order reaction kinetics fittings (D) of 1-hexene epoxidation, reaction process (E) and pseudo-first order reaction kinetics fittings (F) of 1-octene epoxidation in TS-1-71. Reaction conditions: catalyst 50 mg, olefin 10 mmol, H_2O_2 10 mmol, methanol 10 mL. The temperatures change from 303 to 333 K at 10 K interval.

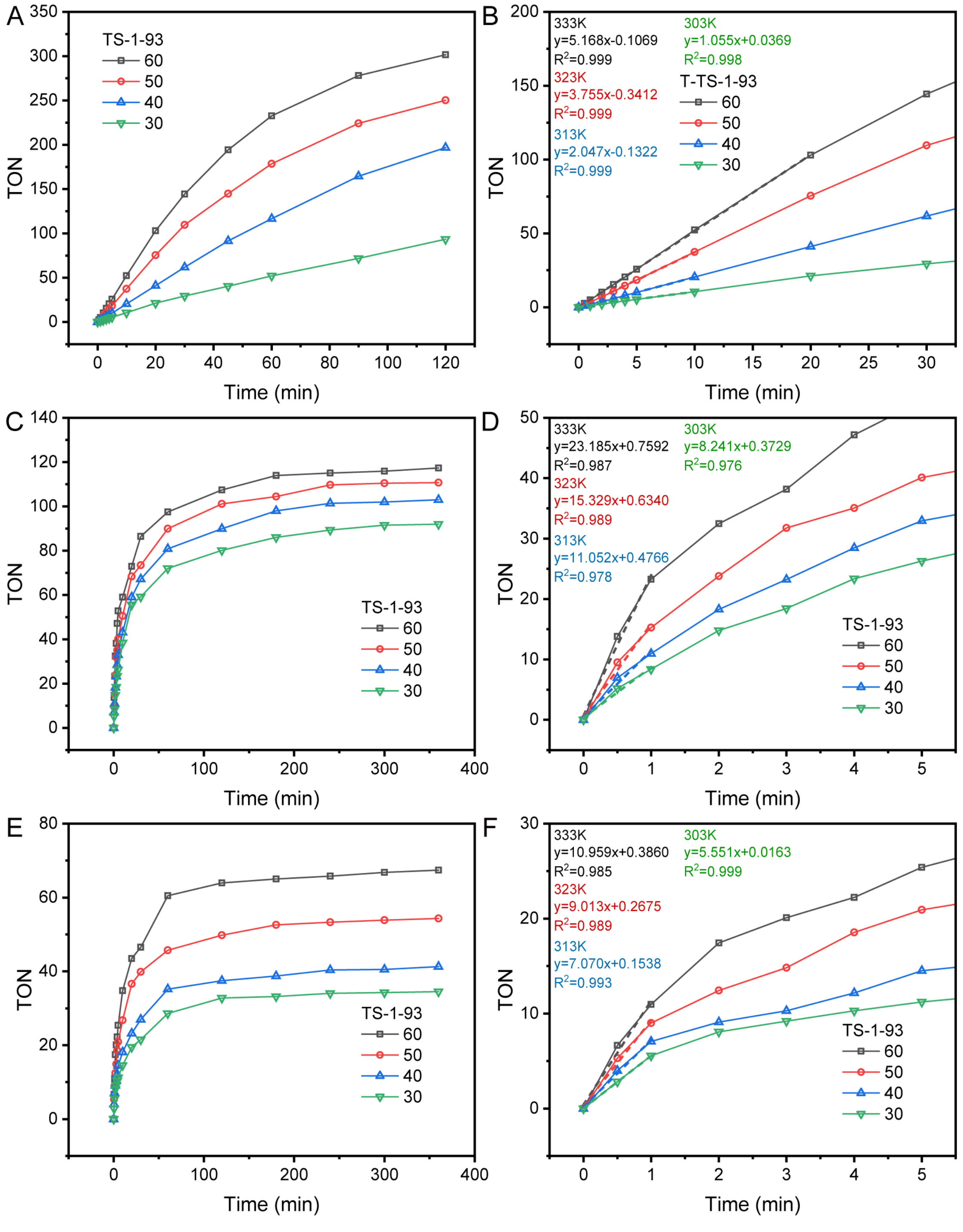

Figure S18. Reaction process (A) and pseudo-first order reaction kinetics fittings (B) of allyl chloride epoxidation, reaction process (C) and pseudo-first order reaction kinetics fittings (D) of 1-hexene epoxidation, reaction process (E) and pseudo-first order reaction kinetics fittings (F) of 1-octene epoxidation in TS-1-93. Reaction conditions: catalyst 50 mg, olefin 10 mmol, H_2O_2 10 mmol, methanol 10 mL. The temperatures change from 303 to 333 K at 10 K interval.

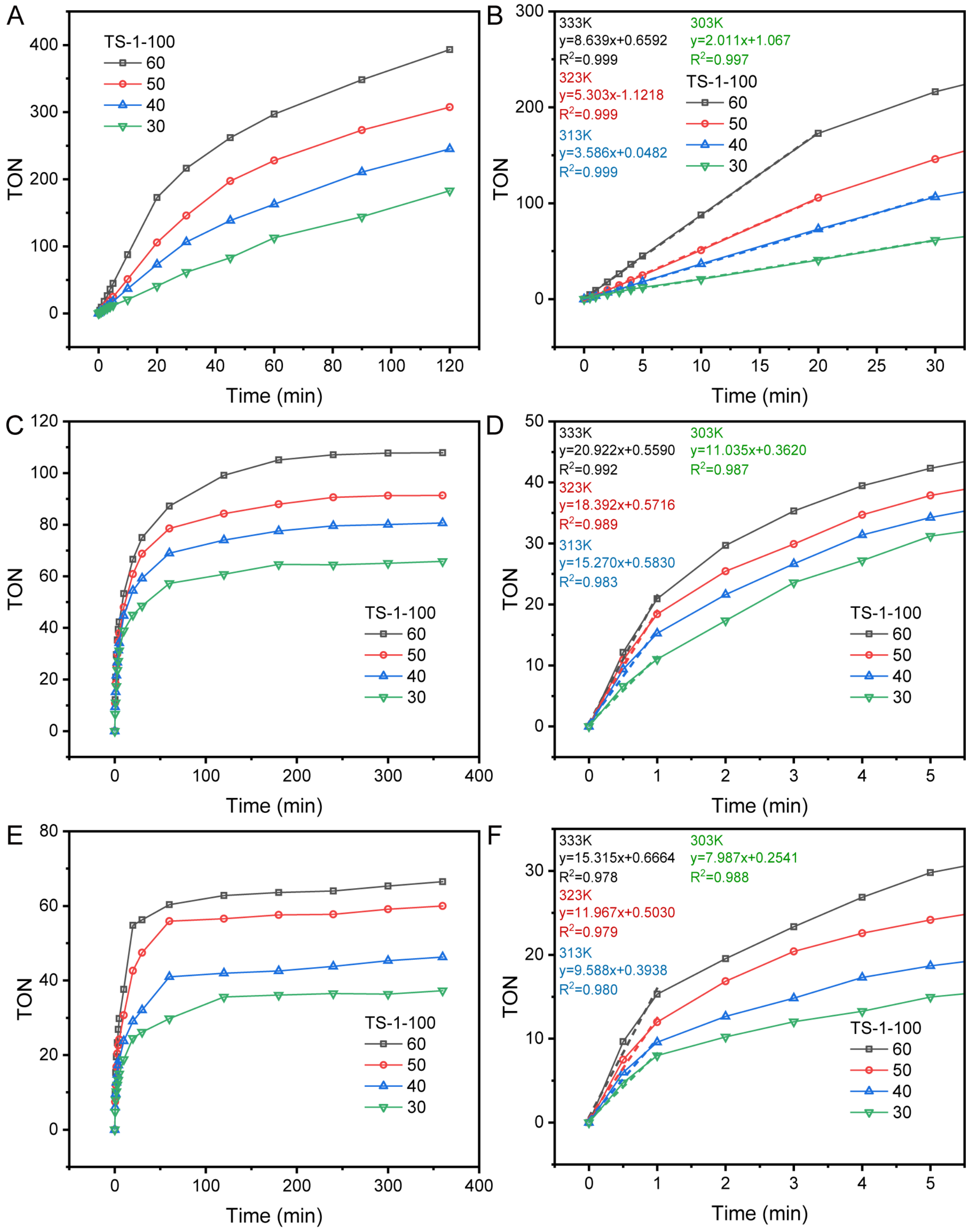

Figure S19. Reaction process (A) and pseudo-first order reaction kinetics fittings (B) of allyl chloride epoxidation, reaction process (C) and pseudo-first order reaction kinetics fittings (D) of 1-hexene epoxidation, reaction process (E) and pseudo-first order reaction kinetics fittings (F) of 1-octene epoxidation in TS-1-100. Reaction conditions: catalyst 50 mg, olefin 10 mmol, H_2O_2 10 mmol, methanol 10 mL. The temperatures change from 303 to 333 K at 10 K interval.

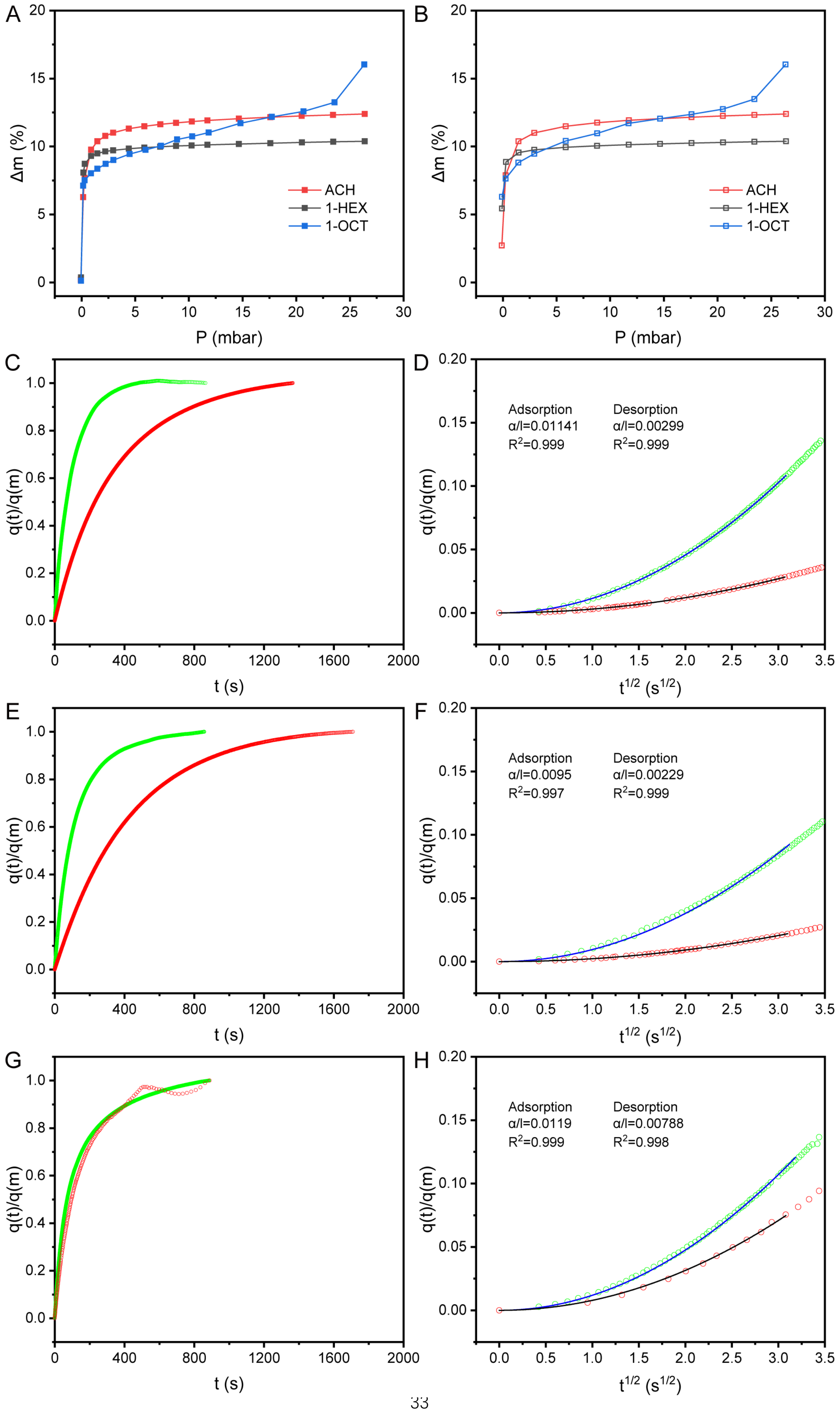
A
B
ACH
1-HEX
1-OCT
Δm (%)
P (mbar)
C
D
E
F
G
H
q(t)/q(m)
t (s)
t^1/2 (s^1/2)
Adsorption
α/l=0.01141
R²=0.999
Desorption
α/l=0.00299
R²=0.999
Adsorption
α/l=0.0095
R²=0.997
Desorption
α/l=0.00229
R²=0.999
Adsorption
α/l=0.0119
R²=0.999
Desorption
α/l=0.00788
R²=0.998

Figure S20. Adsorption (A) and desorption (B) isotherms of allyl chloride, 1-hexene and 1-octene in TS-1-43. Kinetic uptake curves of allyl chloride (C), 1-hexene (E) and 1-octene (G) and kinetic uptake curves and kinetic fitting profiles with SBM of allyl chloride (D), 1-hexene (F) and 1-octene (H) in a short time domain in TS-1-43. The pressure range of adsorption kinetic uptake curves is from 0 to 0.15 mbar. The pressure range of desorption kinetic uptake curves is from 0.26 mbar to 0. The green circles and red circles are the experimental data of adsorption and desorption, respectively. The solid lines refer to the fitting curves. The parameter $\frac{\alpha}{l}$ refers to that in SBM.

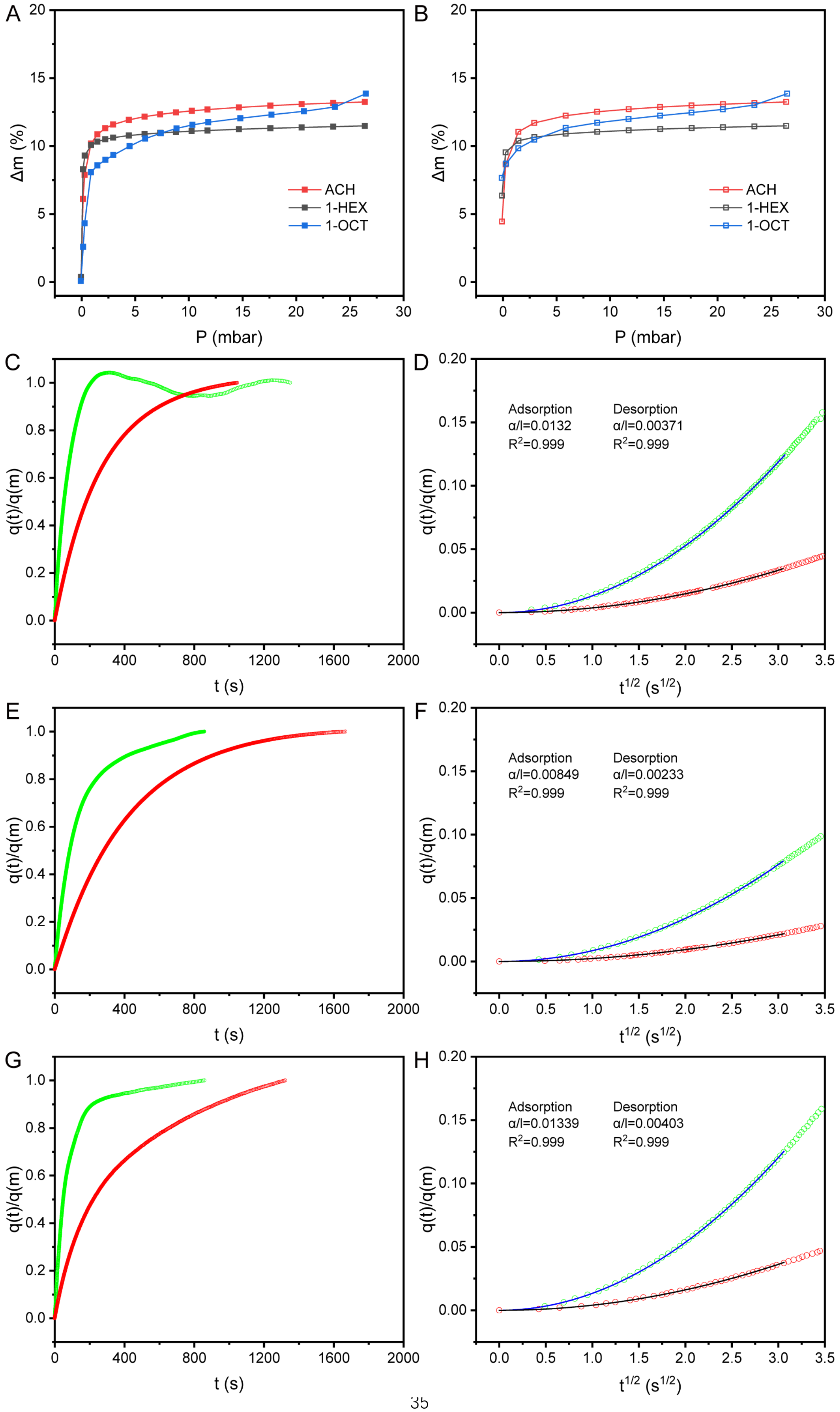
A
B
Δm (%)
P (mbar)
ACH
1-HEX
1-OCT
C
D
E
F
G
H
q(t)/q(m)
t (s)
t^1/2 (s^1/2)
Adsorption
α/l=0.0132
R2=0.999
Desorption
α/l=0.00371
R2=0.999
Adsorption
α/l=0.00849
R2=0.999
Desorption
α/l=0.00233
R2=0.999
Adsorption
α/l=0.01339
R2=0.999
Desorption
α/l=0.00403
R2=0.999

Figure S21. Adsorption (A) and desorption (B) isotherms of allyl chloride, 1-hexene and 1-octene in TS-1-71. Kinetic uptake curves of allyl chloride (C), 1-hexene (E) and 1-octene (G) and kinetic uptake curves and kinetic fitting profiles with SBM of allyl chloride (D), 1-hexene (F) and 1-octene (H) in a short time domain in TS-1-71. The pressure range of adsorption kinetic uptake curves is from 0 to 0.15 mbar. The pressure range of desorption kinetic uptake curves is from 0.26 mbar to 0. The green circles and red circles are the experimental data of adsorption and desorption, respectively. The solid lines refer to the fitting curves. The parameter $\frac{\alpha}{l}$ refers to that in SBM.

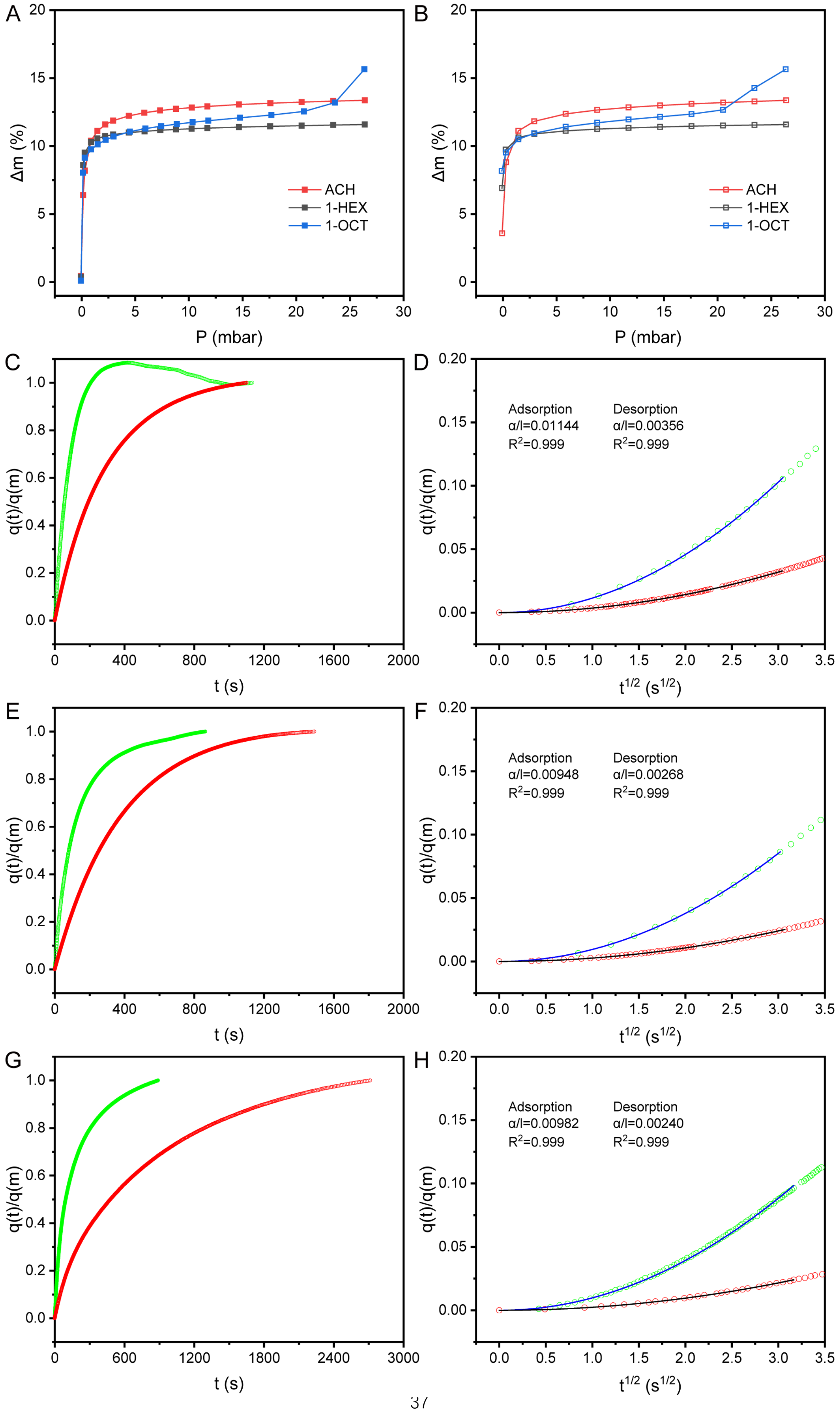
A
B
C
D
E
F
G
H
Δm (%)
P (mbar)
ACH
1-HEX
1-OCT
q(t)/q(m)
t (s)
$t^{1/2}$ ($s^{1/2}$)
Adsorption
α/l=0.01144
R^2=0.999
Desorption
α/l=0.00356
R^2=0.999
Adsorption
α/l=0.00948
R^2=0.999
Desorption
α/l=0.00268
R^2=0.999
Adsorption
α/l=0.00982
R^2=0.999
Desorption
α/l=0.00240
R^2=0.999

Figure S22. Adsorption (A) and desorption (B) isotherms of allyl chloride, 1-hexene and 1-octene in TS-1-93. Kinetic uptake curves of allyl chloride (C), 1-hexene (E) and 1-octene (G) and kinetic uptake curves and kinetic fitting profiles with SBM of allyl chloride (D), 1-hexene (F) and 1-octene (H) in a short time domain in TS-1-93. The pressure range of adsorption kinetic uptake curves is from 0 to 0.15 mbar. The pressure range of desorption kinetic uptake curves is from 0.26 mbar to 0. The green circles and red circles are the experimental data of adsorption and desorption, respectively. The solid lines refer to the fitting curves. The parameter $\frac{\alpha}{l}$ refers to that in SBM.

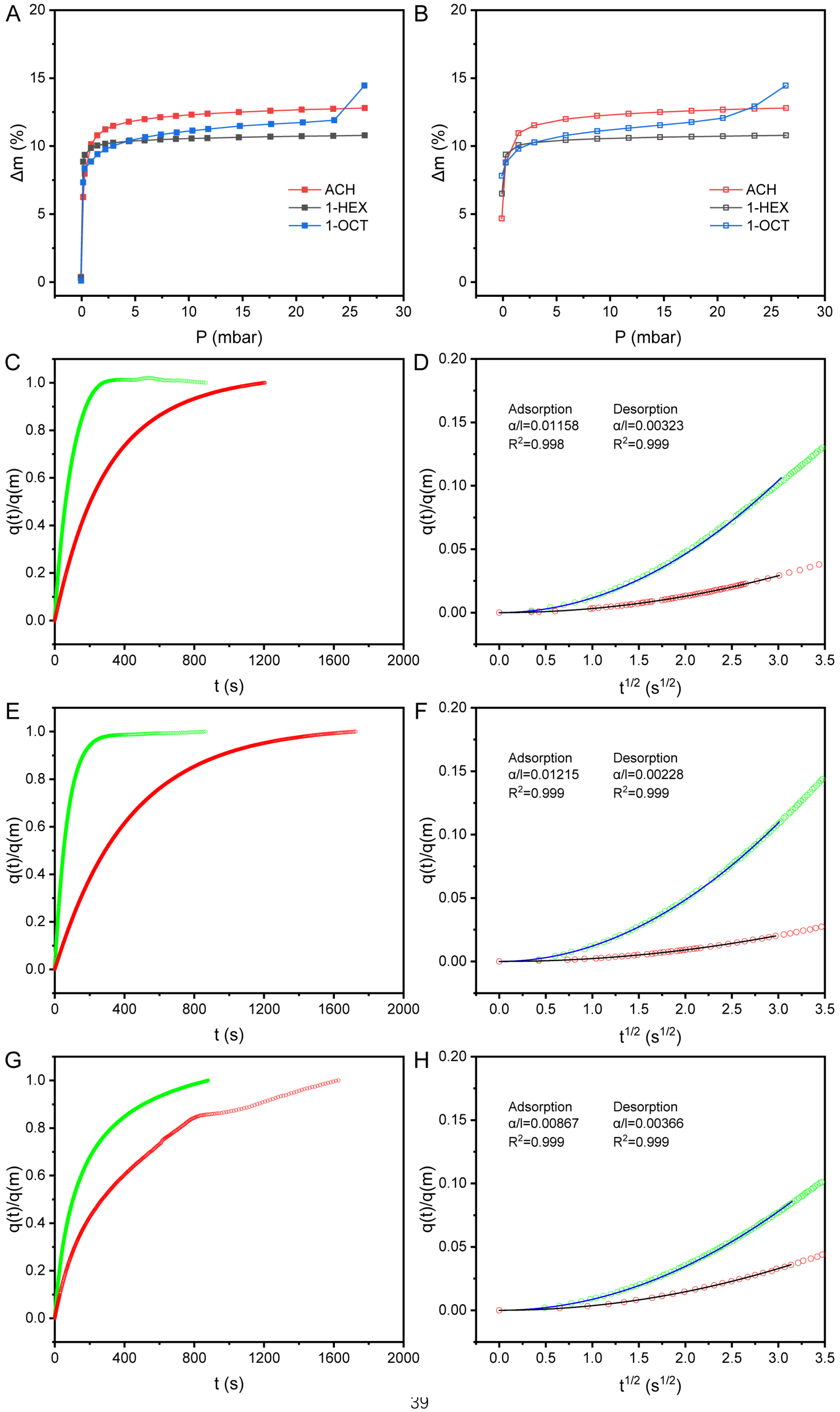

A
B
C
D
E
F
G
H
Δm (%)
P (mbar)
ACH
1-HEX
1-OCT
q(t)/q(m)
t (s)
t^1/2 (s^1/2)
Adsorption
α/l=0.01158
R²=0.998
Desorption
α/l=0.00323
R²=0.999
Adsorption
α/l=0.01215
R²=0.999
Desorption
α/l=0.00228
R²=0.999
Adsorption
α/l=0.00867
R²=0.999
Desorption
α/l=0.00366
R²=0.999

Figure S23. Adsorption (A) and desorption (B) isotherms of allyl chloride, 1-hexene and 1-octene in TS-1-100. Kinetic uptake curves of allyl chloride (C), 1-hexene (E) and 1-octene (G) and kinetic uptake curves and kinetic fitting profiles with SBM of allyl chloride (D), 1-hexene (F) and 1-octene (H) in a short time domain in TS-1-100. The pressure range of adsorption kinetic uptake curves is from 0 to 0.15 mbar. The pressure range of desorption kinetic uptake curves is from 0.26 mbar to 0. The green circles and red circles are the experimental data of adsorption and desorption, respectively. The solid lines refer to the fitting curves. The parameter $\frac{\alpha}{l}$ refers to that in SBM.

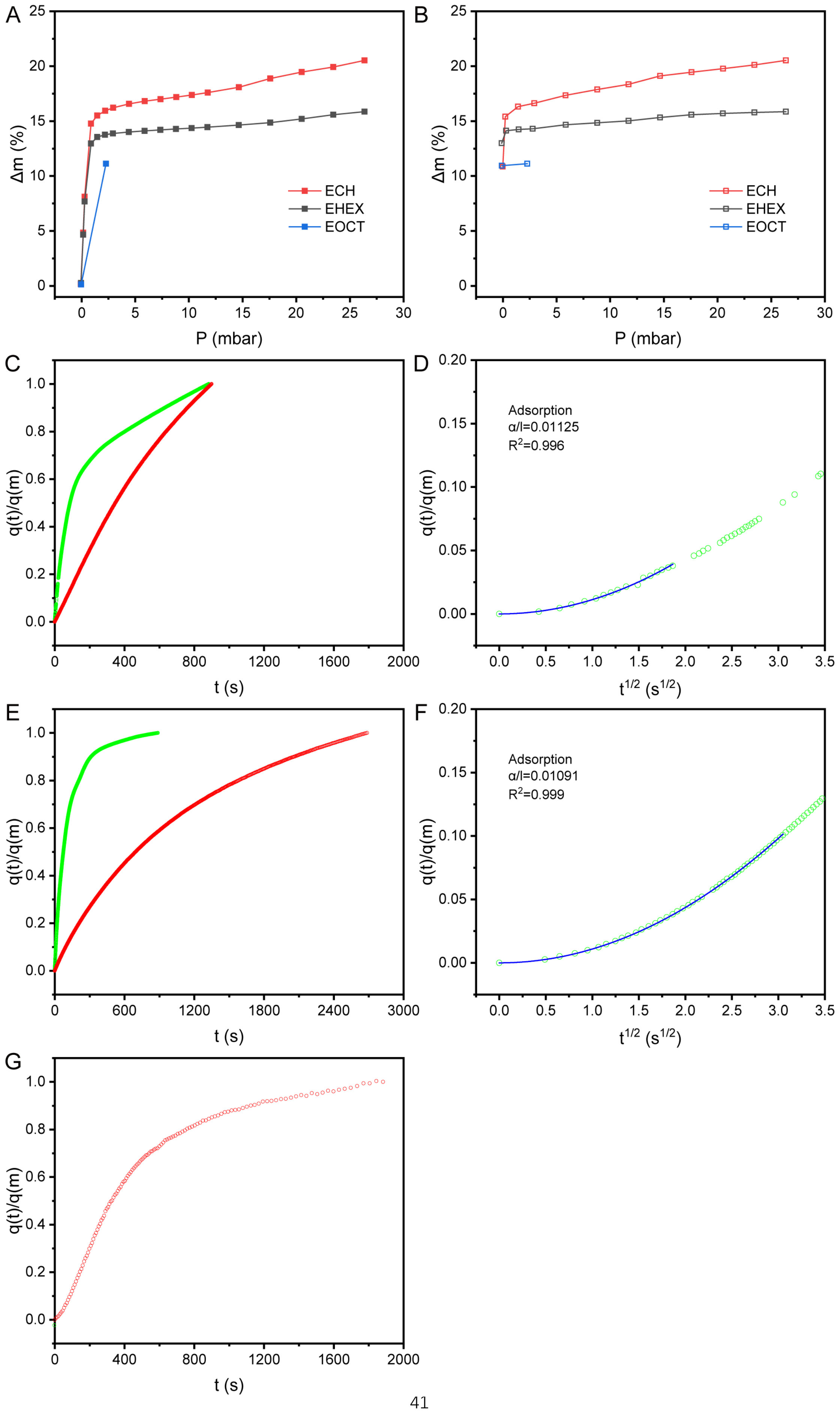
A
B
C
D
E
F
G
Δm (%)
P (mbar)
ECH
EHEX
EOCT
q(t)/q(m)
t (s)
t^1/2 (s^1/2)
Adsorption
α/l=0.01125
R2=0.996
Adsorption
α/l=0.01091
R2=0.999

Figure S24. Adsorption (A) and desorption (B) isotherms of epichlorohydrin, 1,2-epoxyhexane and 1,2-epoxyoctane in TS-1-43. Kinetic uptake curves of epichlorohydrin (C), 1,2-epoxyhexane (E) and 1,2-epoxyoctane (G) and kinetic uptake curves and kinetic fitting profiles with SBM of epichlorohydrin (D) and 1,2-epoxyhexane (F) in a short time domain in TS-1-43. The pressure range of epichlorohydrin and 1,2-epoxyhexane adsorption kinetic uptake curves is from 0 to 0.15 mbar. The pressure range of 1,2-epoxyoctane adsorption kinetic uptake curves is from 0 to 2.27 mbar. The green circles are the experimental data of adsorption. The solid lines refer to the fitting curves. The parameter $\frac{\alpha}{l}$ refers to that in SBM.

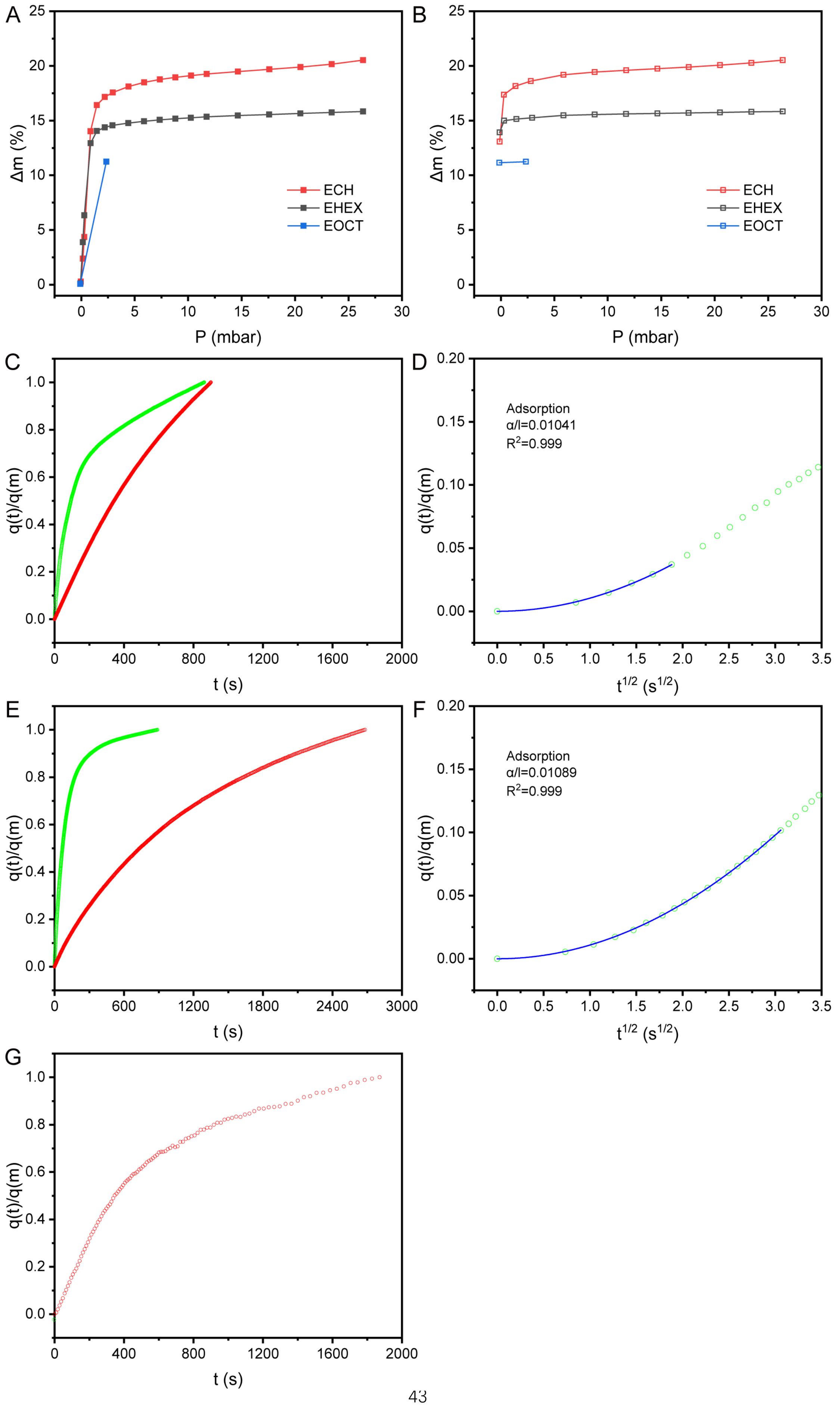
A
B
C
D
E
F
G
Δm (%)
P (mbar)
ECH
EHEX
EOCT
q(t)/q(m)
t (s)
t1/2 (s1/2)
Adsorption
α/l=0.01041
R2=0.999
Adsorption
α/l=0.01089
R2=0.999

Figure S25. Adsorption (A) and desorption (B) isotherms of epichlorohydrin, 1,2-epoxyhexane and 1,2-epoxyoctane in TS-1-71. Kinetic uptake curves of epichlorohydrin (C), 1,2-epoxyhexane (E) and 1,2-epoxyoctane (G) and kinetic uptake curves and kinetic fitting profiles with SBM of epichlorohydrin (D) and 1,2-epoxyhexane (F) in a short time domain in TS-1-71. The pressure range of epichlorohydrin and 1,2-epoxyhexane adsorption kinetic uptake curves is from 0 to 0.15 mbar. The pressure range of 1,2-epoxyoctane adsorption kinetic uptake curves is from 0 to 2.27 mbar. The green circles are the experimental data of adsorption. The solid lines refer to the fitting curves. The parameter $\frac{\alpha}{l}$ refers to that in SBM.

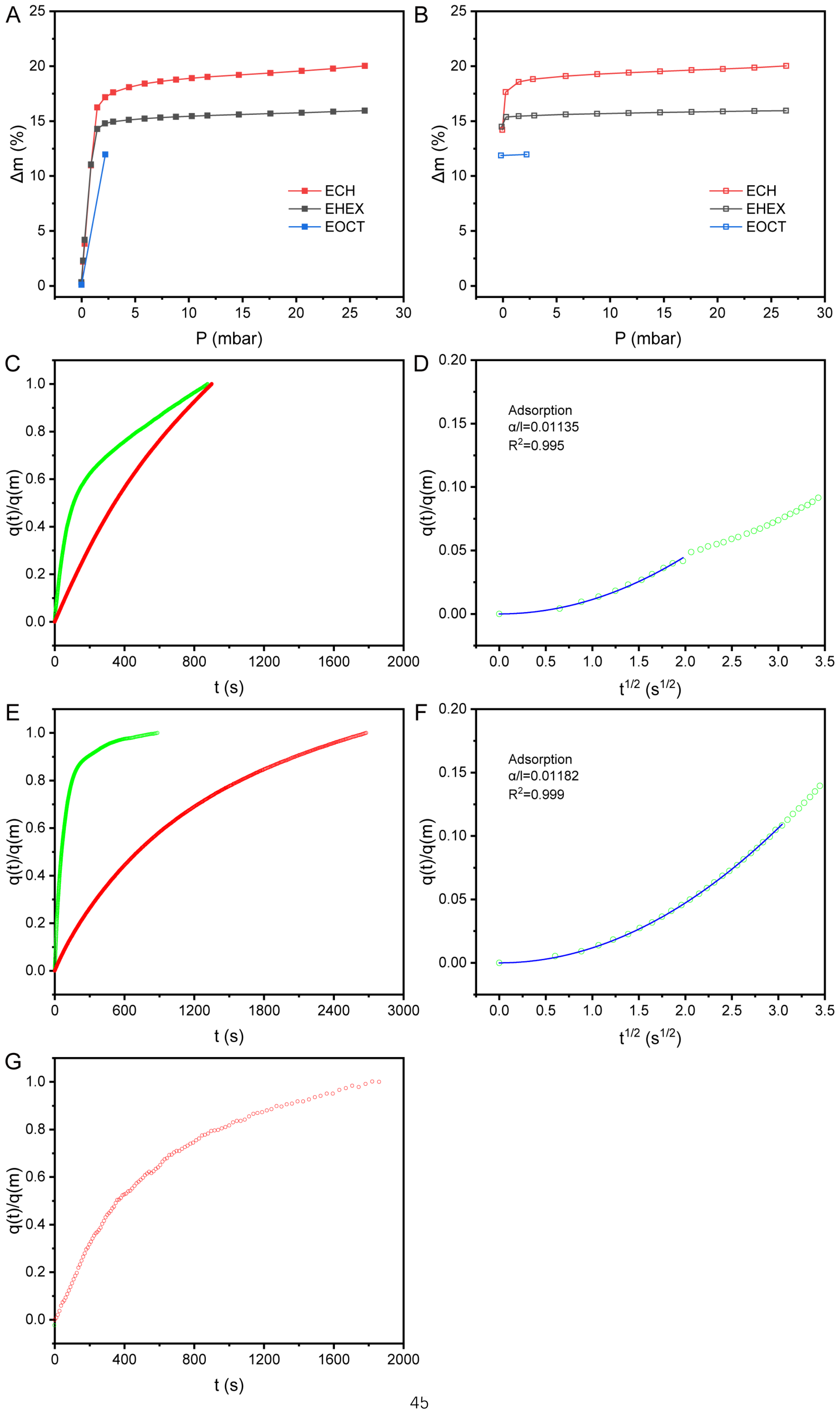
A
B
Δm (%)
P (mbar)
ECH
EHEX
EOCT
C
D
E
F
G
q(t)/q(m)
t (s)
$t^{1/2}$ ($s^{1/2}$)
Adsorption
α/l=0.01135
R^2=0.995
Adsorption
α/l=0.01182
R^2=0.999

Figure S26. Adsorption (A) and desorption (B) isotherms of epichlorohydrin, 1,2-epoxyhexane and 1,2-epoxyoctane in TS-1-93. Kinetic uptake curves of epichlorohydrin (C), 1,2-epoxyhexane (E) and 1,2-epoxyoctane (G) and kinetic uptake curves and kinetic fitting profiles with SBM of epichlorohydrin (D) and 1,2-epoxyhexane (F) in a short time domain in TS-1-93. The pressure range of epichlorohydrin and 1,2-epoxyhexane adsorption kinetic uptake curves is from 0 to 0.15 mbar. The pressure range of 1,2-epoxyoctane adsorption kinetic uptake curves is from 0 to 2.27 mbar. The green circles are the experimental data of adsorption. The solid lines refer to the fitting curves. The parameter $\frac{\alpha}{l}$ refers to that in SBM.

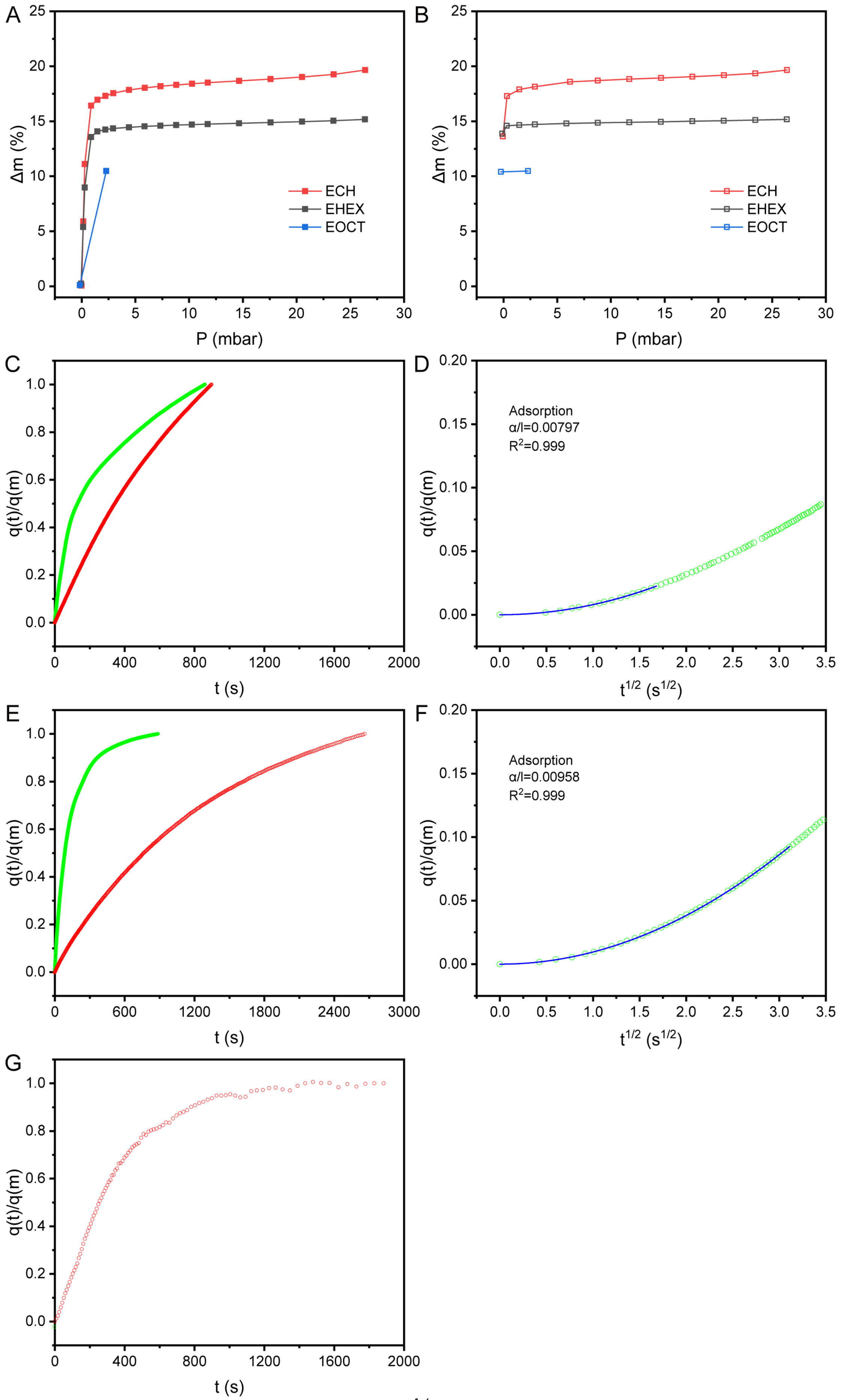
A
Δm (%)
P (mbar)
ECH
EHEX
EOCT
B
Δm (%)
P (mbar)
ECH
EHEX
EOCT
C
q(t)/q(m)
t (s)
D
Adsorption
α/l=0.00797
R2=0.999
q(t)/q(m)
t1/2 (s1/2)
E
q(t)/q(m)
t (s)
F
Adsorption
α/l=0.00958
R2=0.999
q(t)/q(m)
t1/2 (s1/2)
G
q(t)/q(m)
t (s)

Figure S27. Adsorption (A) and desorption (B) isotherms of epichlorohydrin, 1,2-epoxyhexane and 1,2-epoxyoctane in TS-1-100. Kinetic uptake curves of epichlorohydrin (C), 1,2-epoxyhexane (E) and 1,2-epoxyoctane (G) and kinetic uptake curves and kinetic fitting profiles with SBM of epichlorohydrin (D) and 1,2-epoxyhexane (F) in a short time domain in TS-1-100. The pressure range of epichlorohydrin and 1,2-epoxyhexane adsorption kinetic uptake curves is from 0 to 0.15 mbar. The pressure range of 1,2-epoxyoctane adsorption kinetic uptake curves is from 0 to 2.27 mbar. The green circles are the experimental data of adsorption. The solid lines refer to the fitting curves. The parameter $\frac{\alpha}{l}$ refers to that in SBM.

The epoxidation is impacted by the diffusion in catalysts, so adsorption-desorption isotherms and liquid phase breakthrough curves are measured to assess the diffusion in series of samples. The adsorption isotherms of 1-hexene (1-HEX) show the saturation at low relative pressure in all the samples (Figure S20-23), demonstrating the high tendency of 1-HEX going into the pore. The saturation adsorption masses of TS-1-71 and TS-1-93 are both the highest, and that of TS-1-100 and TS-1-43 are the second highest and lowest, respectively. Given the similar micropore volume, such disparity may originate from the diversity of space-confined structures. As shown in the adsorption isotherms of 1,2-epoxyhexane (EHEX) (Figure S24-27), the low saturation relative pressure is recorded, so it is also easy for EHEX to come into the pore as well just like 1-HEX. However, the adsorption mass of TS-1-43 overtakes that of TS-1-100 at high relative pressure, which is assigned to the condensability of high-boiling EHEX on TS-1-43 with large mesopore volume. The desorption isotherms of 1-HEX and EHEX almost match up the corresponding adsorption isotherms. Nevertheless, there are still about 5 % 1-HEX and 12 % EHEX remaining in the pores even after reaching the settled desorption point as a result of the slow desorption dynamics and finite desorption time. The desorption of EHEX is more difficult than that of 1-HEX considering more remnant after desorption, accounting for the tougher interaction between strong polar EHEX and the pore wall. Moreover, the amount of desorbed

EHEX exhibits negative correlation with the hydrophilicity of TS-1 absorbents by reason of the great force between EHEX and the hydrophilic pore wall.

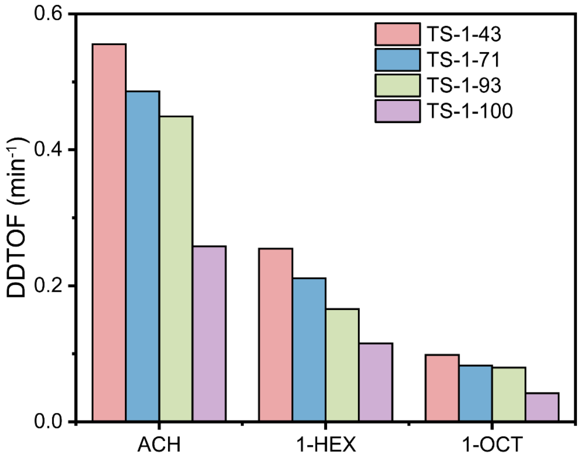

Figure S28. DDTOF of alkene measured in gas phase.

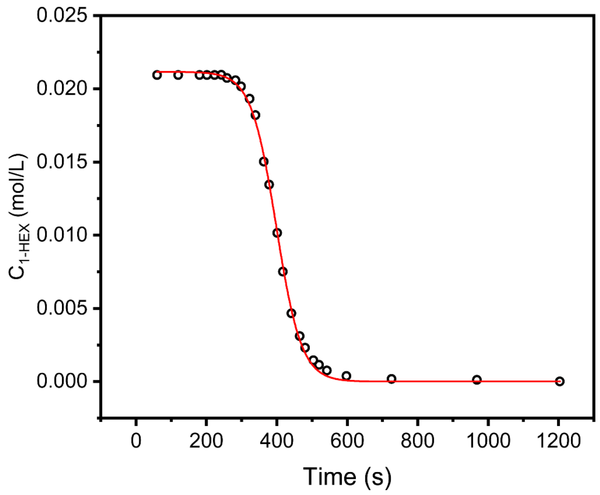

Figure S29. The breakthrough curve and fitting profile in empty tube. 1-hexene remained in the tube is excluded by trimethylbenzene. The black circles and red line are experimental data and fitting curve, respectively. The calculation is based on sigma fitting method.

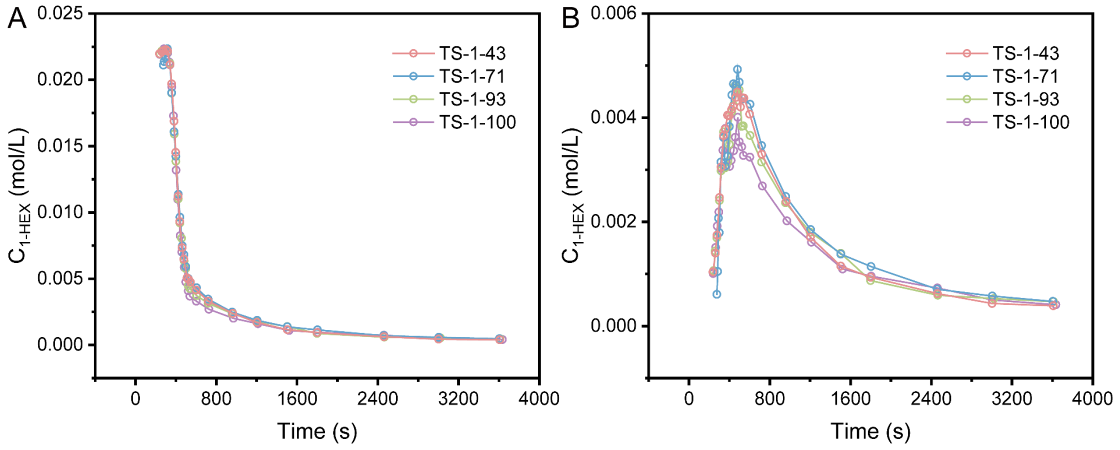

Figure S30. The breakthrough curves before (A) and after (B) empty tube background correction. 1-hexene adsorbed previously in samples is excluded by trimethylbenzene.

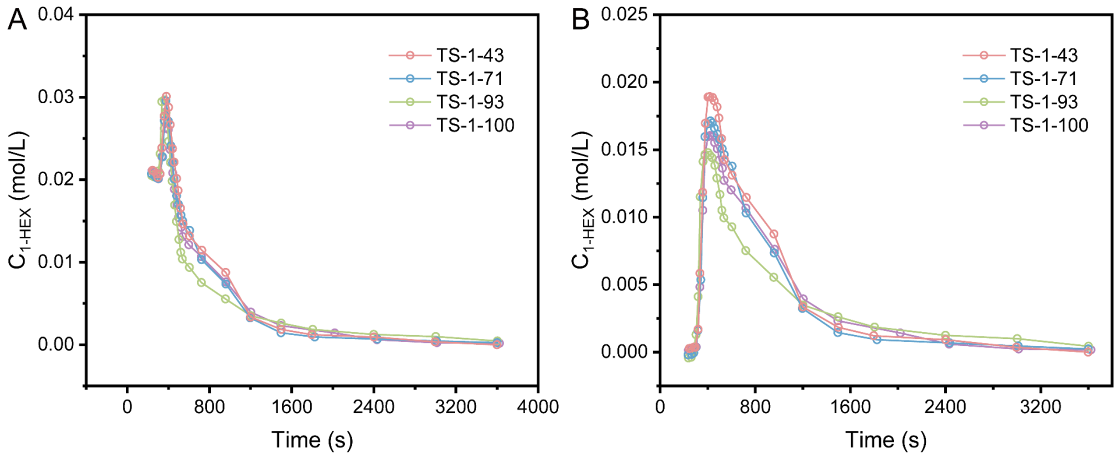

Figure S31. The breakthrough curves before (A) and after (B) empty tube background correction. 1-hexene adsorbed previously in samples is excluded by 0.2 wt.% methanol trimethylbenzene solution.

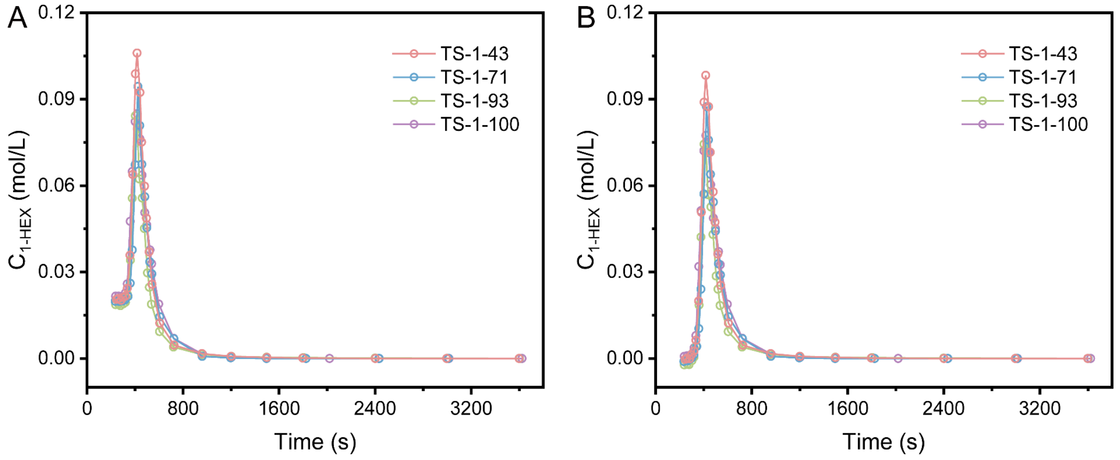

Figure S32. The breakthrough curves before (A) and after (B) empty tube background correction. 1-hexene adsorbed previously in samples is excluded by 2 wt.% methanol trimethylbenzene solution.

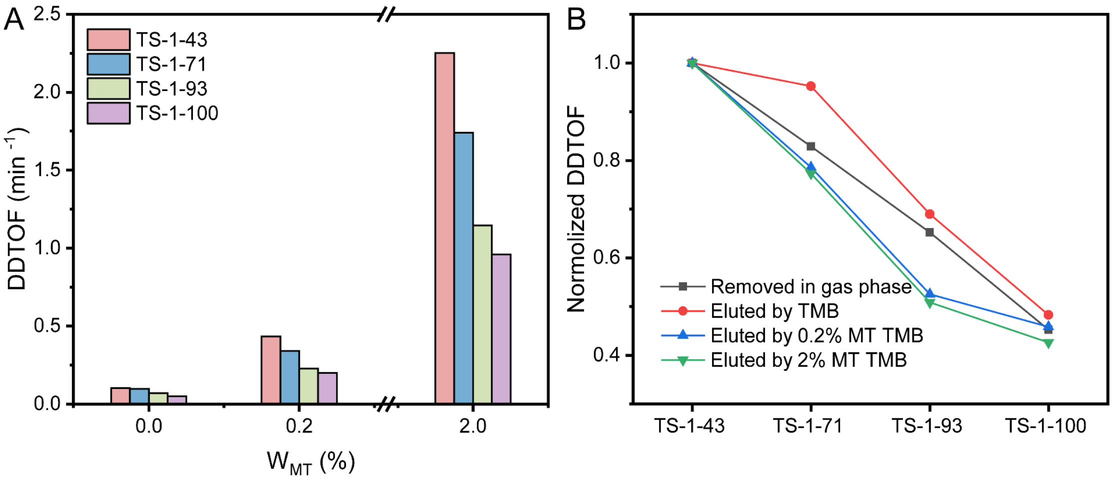

Figure S33. DDTOFs of 1-HEX measured in flowing methanol trimethylbenzene solution (A) and normalized DDTOFs of samples under different desorption conditions (B).

The liquid phase breakthrough curves of 1-HEX are collected to simulate the desorption in practical epoxidation. 1-HEX instead of EHEX is selected thanks to its stability against methanol with the existence of TS-1, and the DDTOFs are calculated when methanol 1,3,5-trimethylbenzene (TMB) solution with different concentrations are used as eluents. A lower DDTOF is found when eluted with pure TMB than that in gas phase. With the increasing methanol concentration in eluents, the DDTOFs increase, demonstrating the accelerated 1-HEX exclusion from the pores in TS-1 under the squeezing from methanol. Restricted by the structural stability of sample bed layer, methanol in eluents is far more diluted from actual epoxidation systems. Therefore, it is possible to reach a rapid diffusion during reaction which is faster than that observed in gas phase.

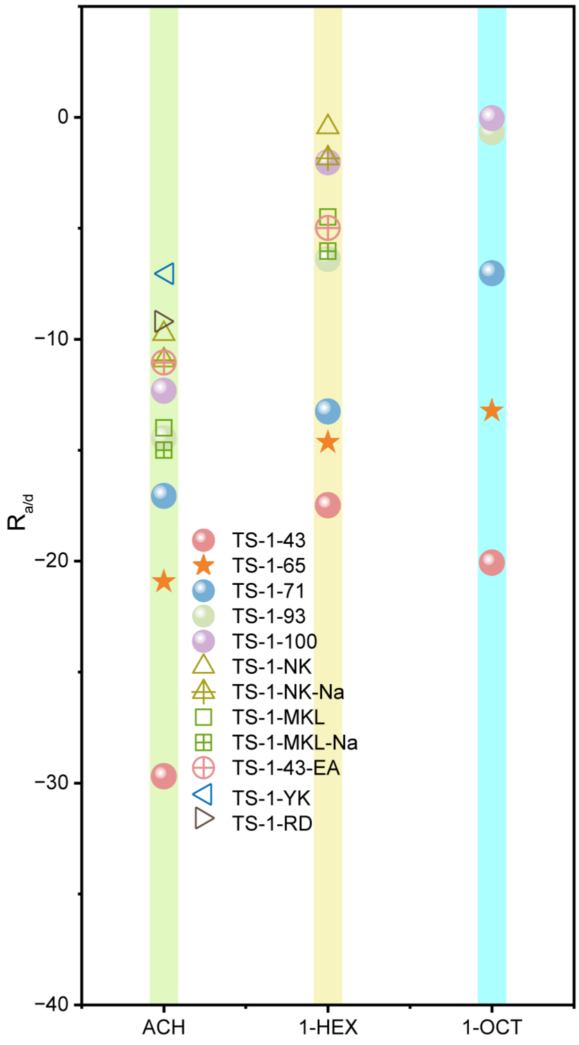

Figure S34. $R_{a/d}$ values of TS-1 samples.

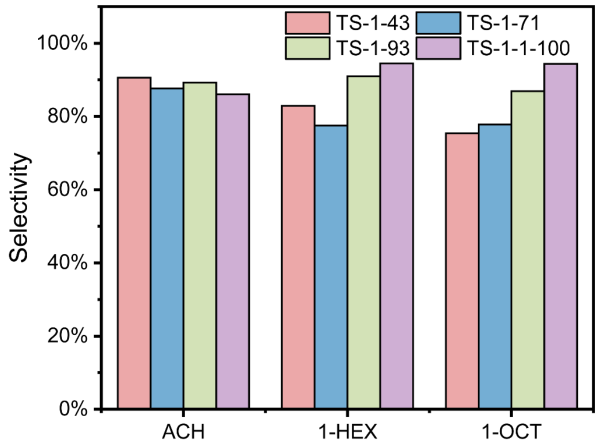

Figure S35. Epoxide selectivity in ACH, 1-HEX and 1-OCT epoxidation.

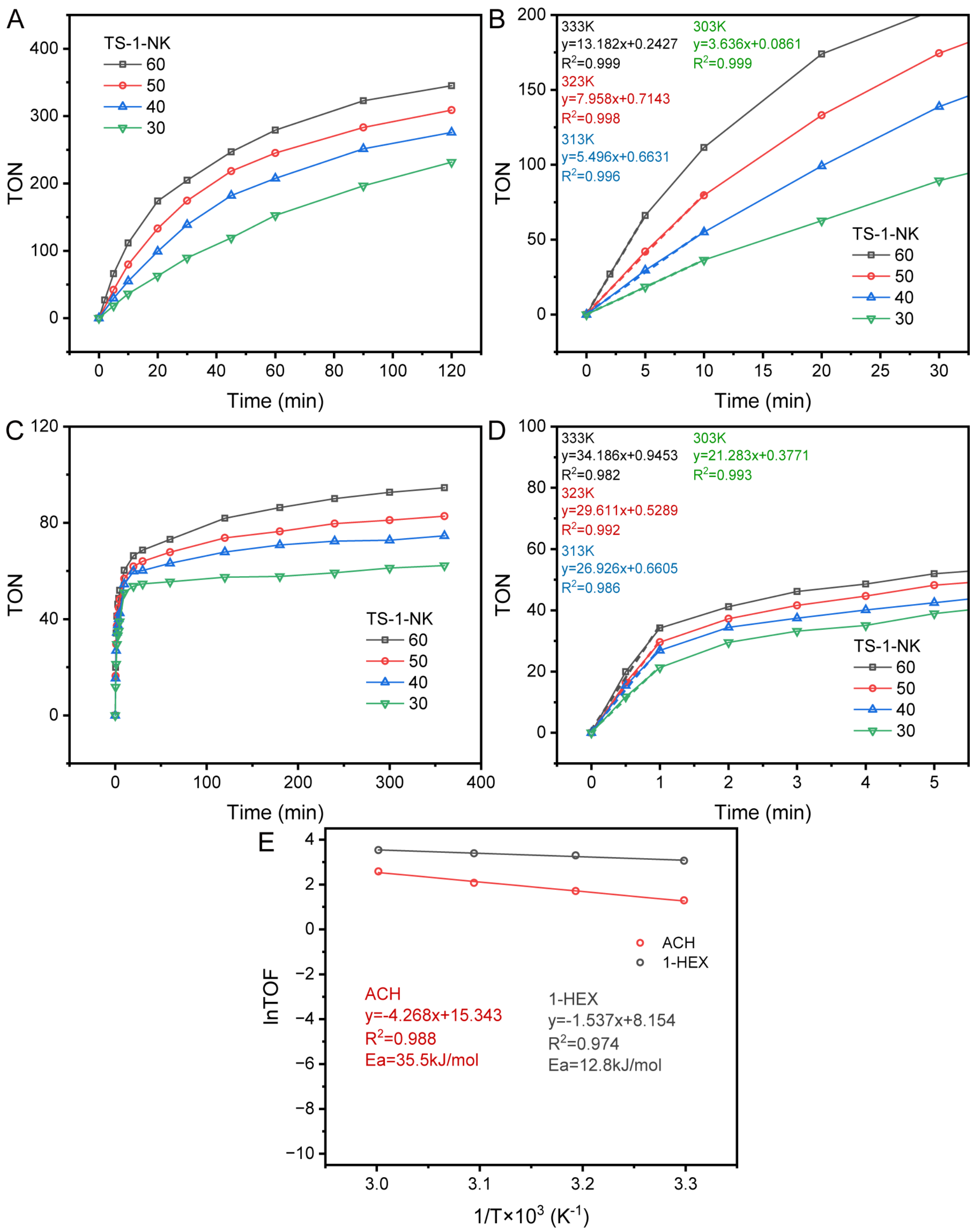

Figure S36. Reaction process (A) and pseudo-first order reaction kinetics fittings (B) of allyl chloride epoxidation and reaction process (C) and pseudo-first order reaction kinetics fittings (D) of 1-hexene epoxidation and Arrhenius plots of allyl chloride, 1-hexene epoxidation (E) in TS-1-NK. Reaction conditions: catalyst 50 mg, olefin 10 mmol, H_2O_2 10 mmol, methanol 10 mL. The temperatures change from 303 to 333 K at 10 K interval.

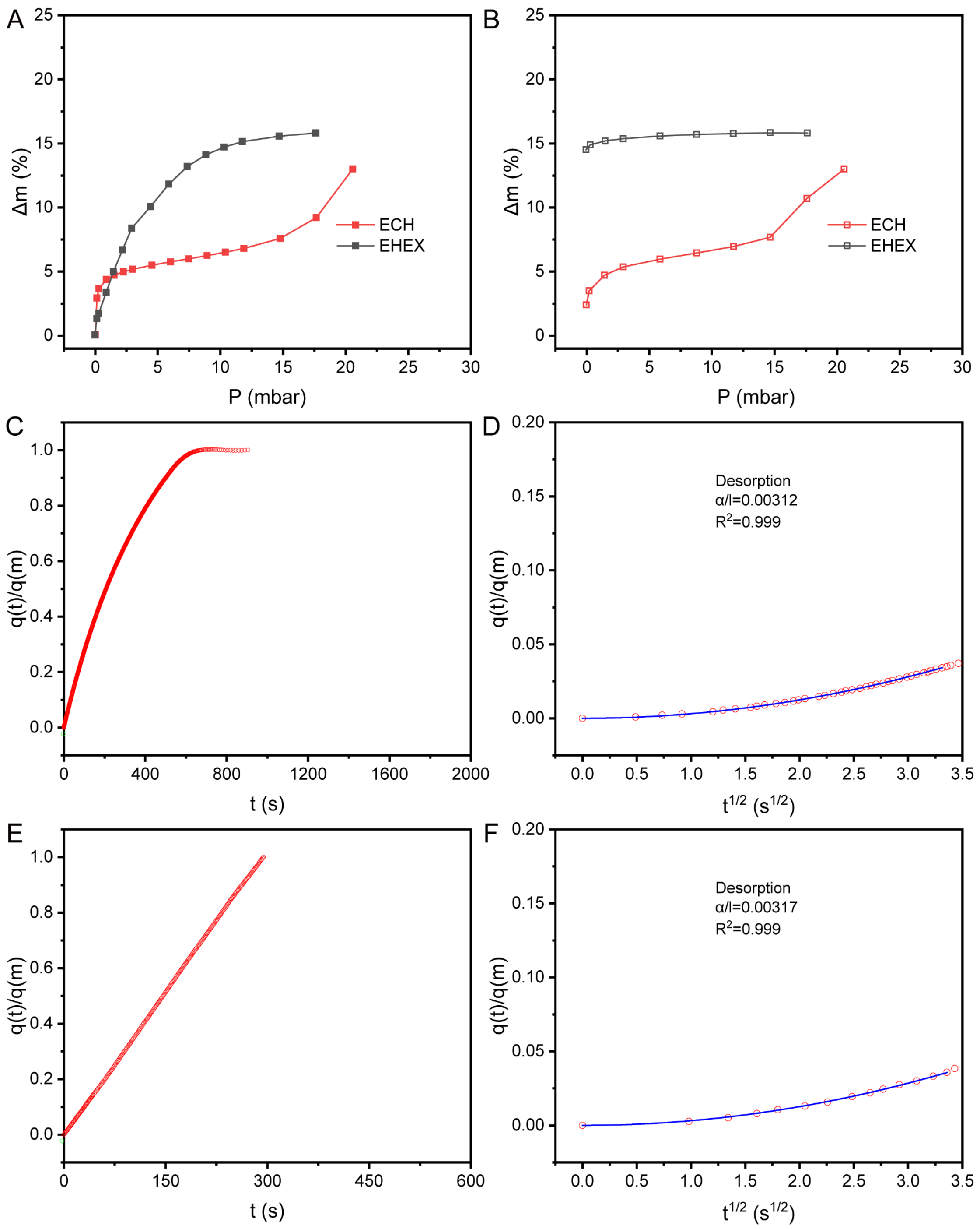

Figure S37. Adsorption (A) and desorption (B) isotherms of epichlorohydrin and 1,2-epoxyhexane in TS-1-NK. Kinetic uptake curves of epichlorohydrin (C) and 1,2-epoxyhexane (E) and kinetic uptake curves and kinetic fitting profiles with SBM of epichlorohydrin (D) and 1,2-epoxyhexane (F) in a short time domain in TS-1-NK. The pressure range of adsorption kinetic uptake curves is from 0 to 0.15 mbar. The pressure range of desorption kinetic uptake curves is from 0.26 mbar to 0. The red circles are the

experimental data of desorption. The solid lines refer to the fitting curves. The parameter $\frac{\alpha}{l}$ refers to that in SBM.

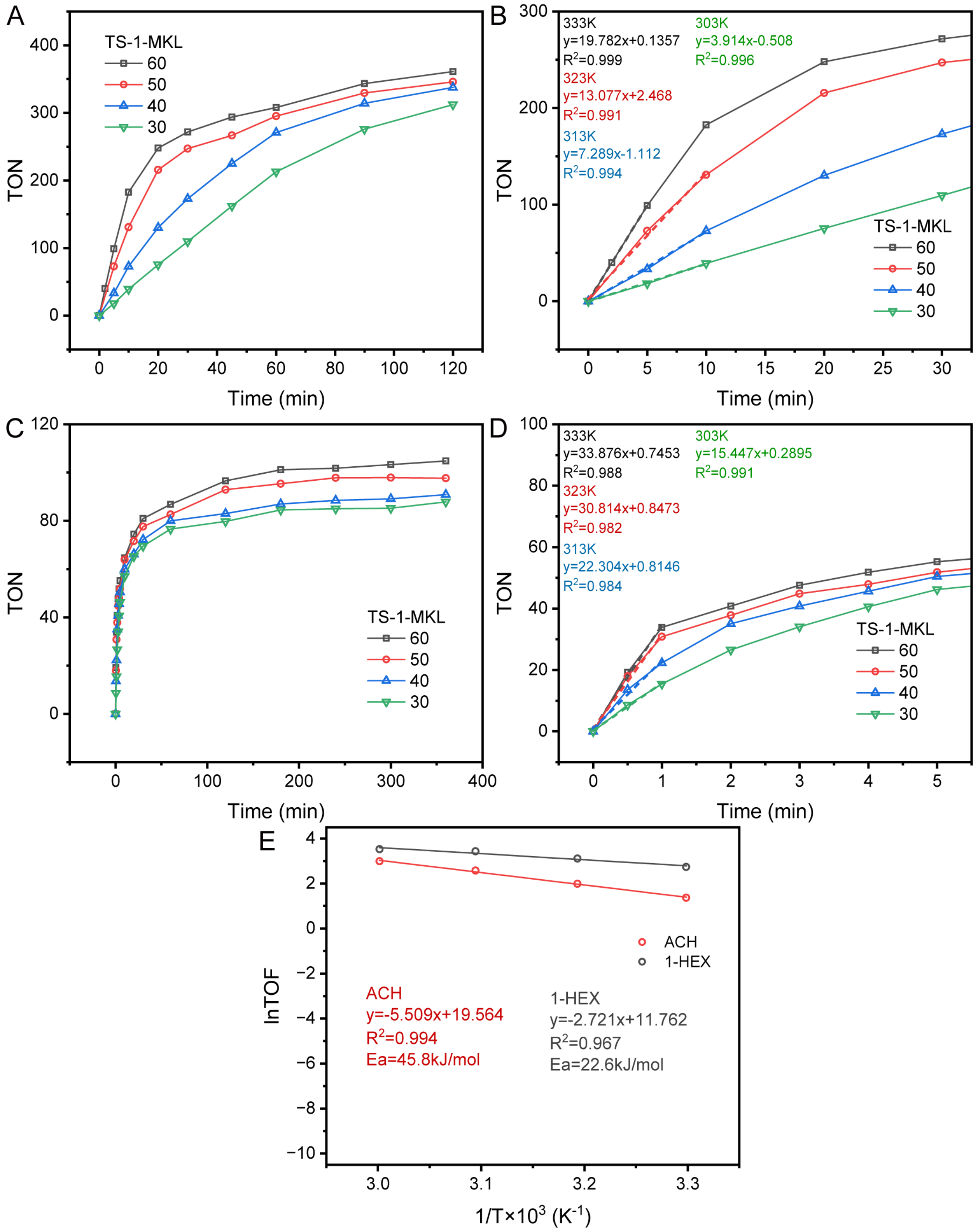

Figure S38. Reaction process (A) and pseudo-first order reaction kinetics fittings (B) of allyl chloride epoxidation and reaction process (C) and pseudo-first order reaction kinetics fittings (D) of 1-hexene epoxidation and Arrhenius plots of allyl chloride, 1-hexene epoxidation (E) in TS-1-MKL. Reaction conditions: catalyst 50 mg, olefin 10 mmol, H_2O_2 10 mmol, methanol 10 mL. The temperatures change from 303 to 333 K at 10 K interval.

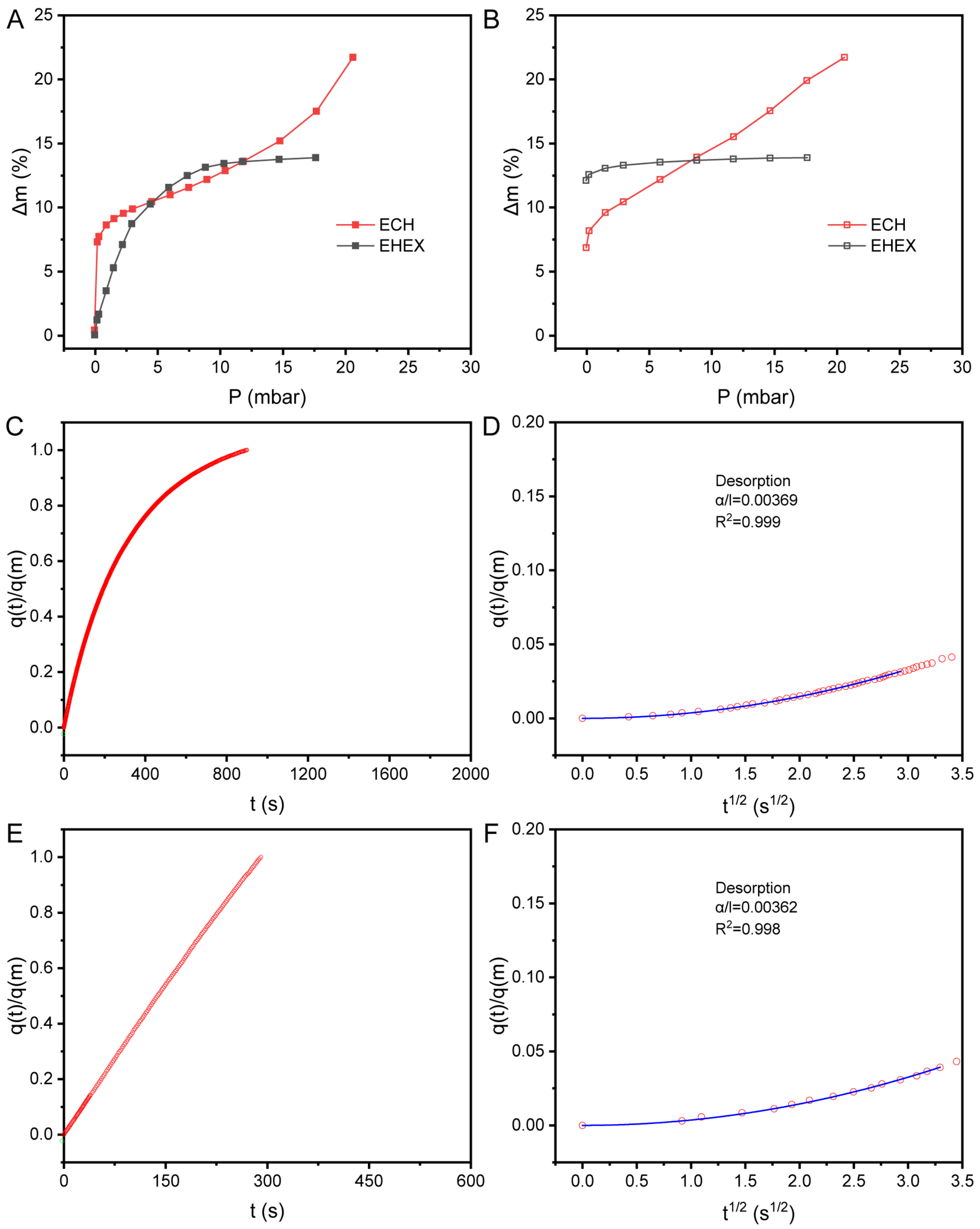

Figure S39. Adsorption (A) and desorption (B) isotherms of epichlorohydrin and 1,2-epoxyhexane in TS-1-MKL. Kinetic uptake curves of epichlorohydrin (C) and 1,2-epoxyhexane (E) and kinetic uptake curves and kinetic fitting profiles with SBM of epichlorohydrin (D) and 1,2-epoxyhexane (F) in a short time domain in TS-1-MKL. The pressure range of adsorption kinetic uptake curves is from 0 to 0.15 mbar. The pressure range of desorption kinetic uptake curves is from 0.26 mbar to 0. The red

circles are the experimental data of desorption. The solid lines refer to the fitting curves. The parameter $\frac{\alpha}{l}$ refers to that in SBM.

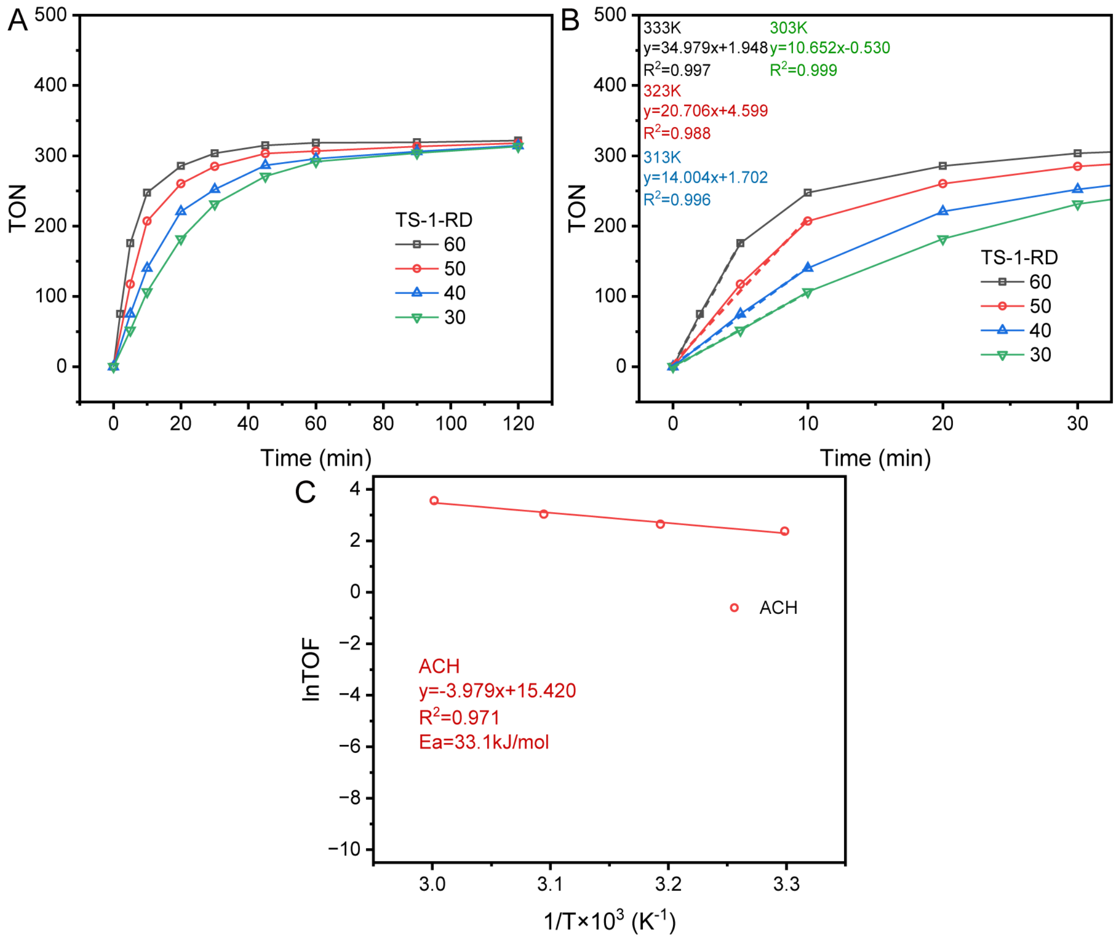

Figure S40. Reaction process (A) and pseudo-first order reaction kinetics fittings (B) of allyl chloride and Arrhenius plots of allyl chloride epoxidation (C) in TS-1-RD. Reaction conditions: catalyst 50 mg, olefin 10 mmol, H_2O_2 10 mmol, methanol 10 mL. The temperatures change from 303 to 333 K at 10 K interval.

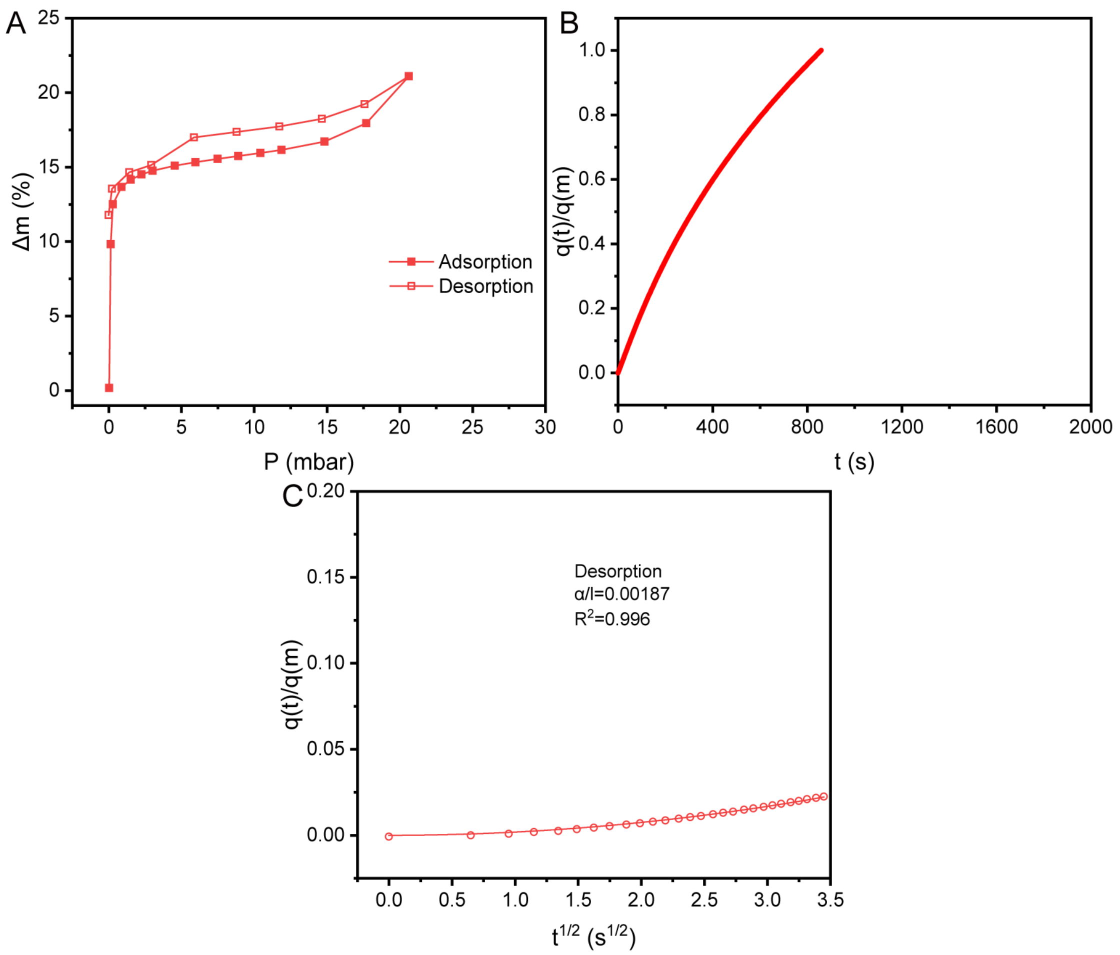

Figure S41. Adsorption and desorption (A) isotherms of epichlorohydrin in TS-1-RD. Kinetic uptake curves of epichlorohydrin (B) and kinetic uptake curves and kinetic fitting profiles with SBM of epichlorohydrin (C) in a short time domain in TS-1-RD. The pressure range of adsorption kinetic uptake curves is from 0 to 0.15 mbar. The pressure range of desorption kinetic uptake curves is from 0.26 mbar to 0. The red circles are the experimental data of desorption. The solid lines refer to the fitting curves. The parameter $\frac{\alpha}{l}$ refers to that in SBM.

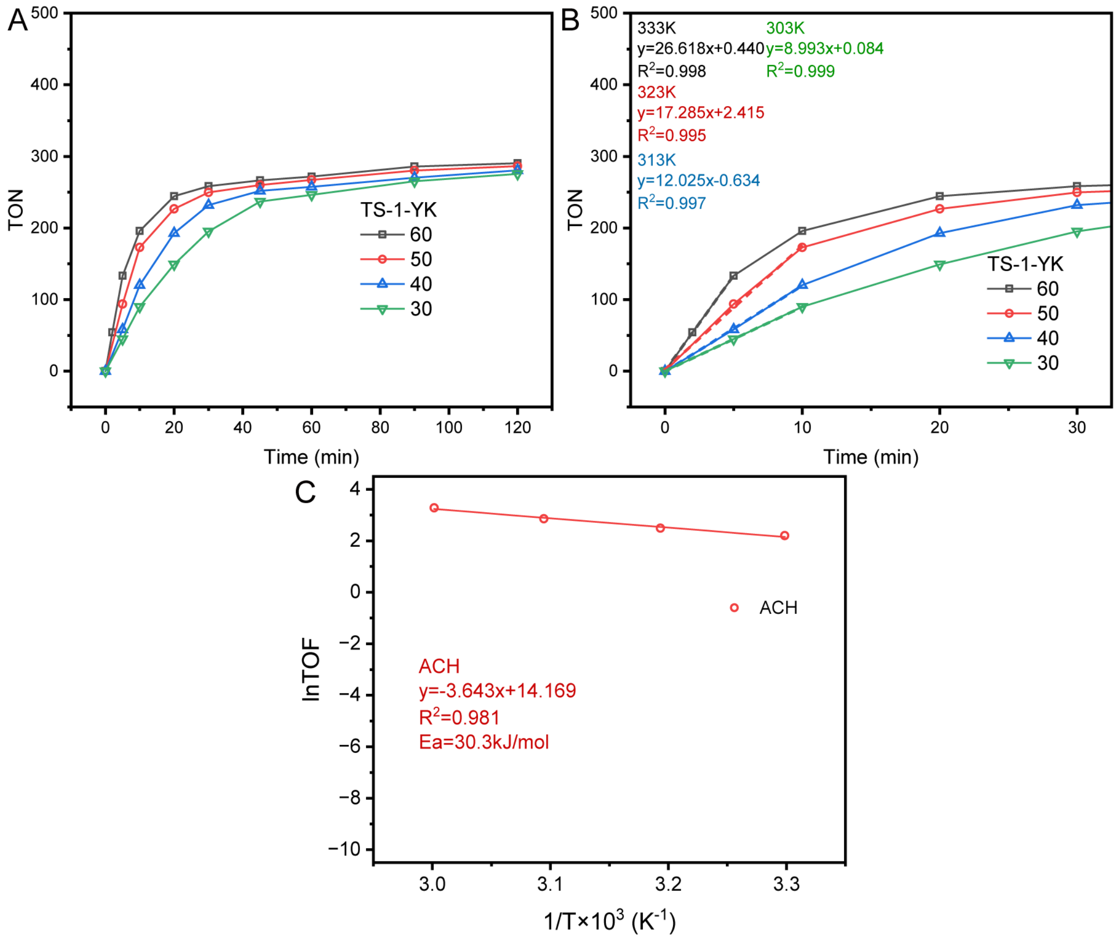

Figure S42. Reaction process (A) and pseudo-first order reaction kinetics fittings (B) of allyl chloride and Arrhenius plots of allyl chloride epoxidation (C) in TS-1-YK. Reaction conditions: catalyst 50 mg, olefin 10 mmol, H_2O_2 10 mmol, methanol 10 mL. The temperatures change from 303 to 333 K at 10 K interval.

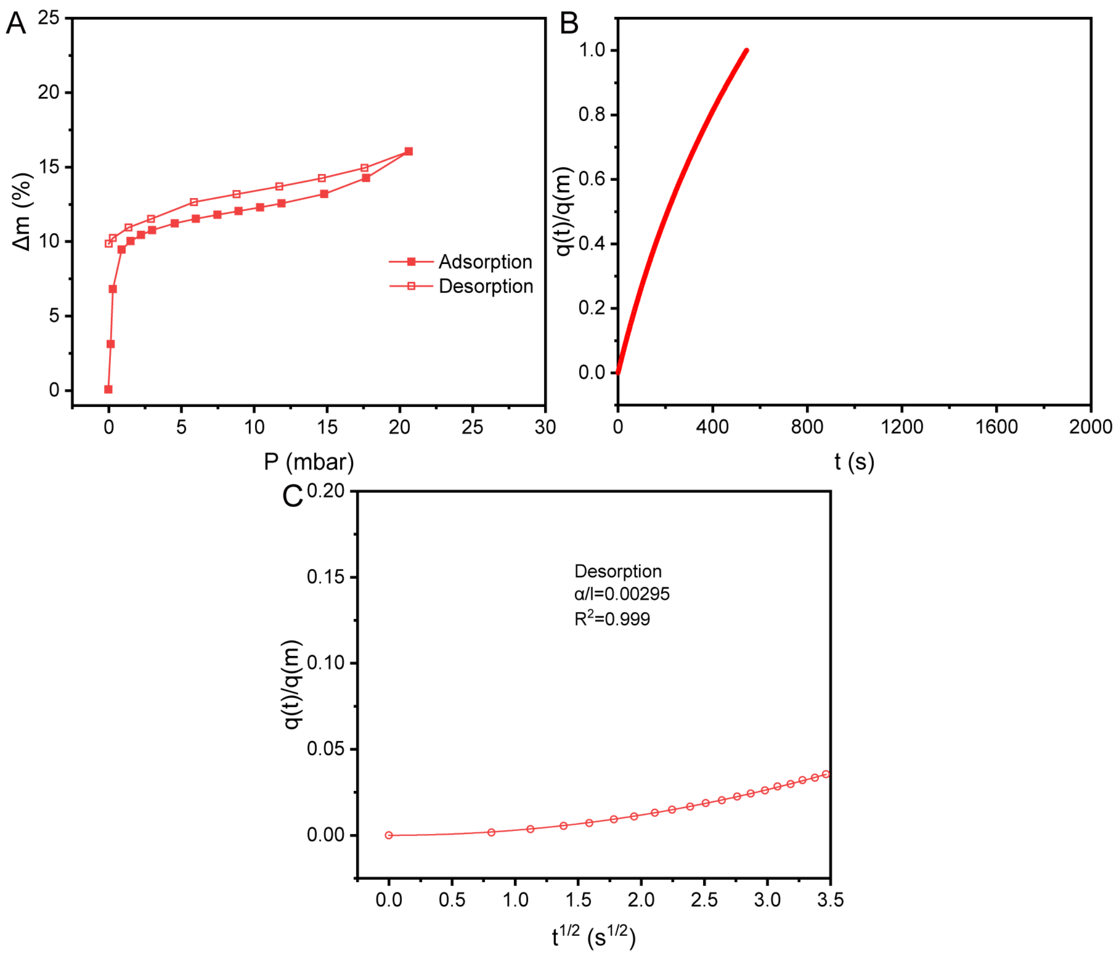

Figure S43. Adsorption and desorption (A) isotherms of epichlorohydrin in TS-1-YK. Kinetic uptake curves of epichlorohydrin (B) and kinetic uptake curves and kinetic fitting profiles with SBM of epichlorohydrin (C) in a short time domain in TS-1-YK. The pressure range of adsorption kinetic uptake curves is from 0 to 0.15 mbar. The pressure range of desorption kinetic uptake curves is from 0.26 mbar to 0. The red circles are the experimental data of desorption. The solid lines refer to the fitting curves. The parameter $\frac{\alpha}{l}$ refers to that in SBM.

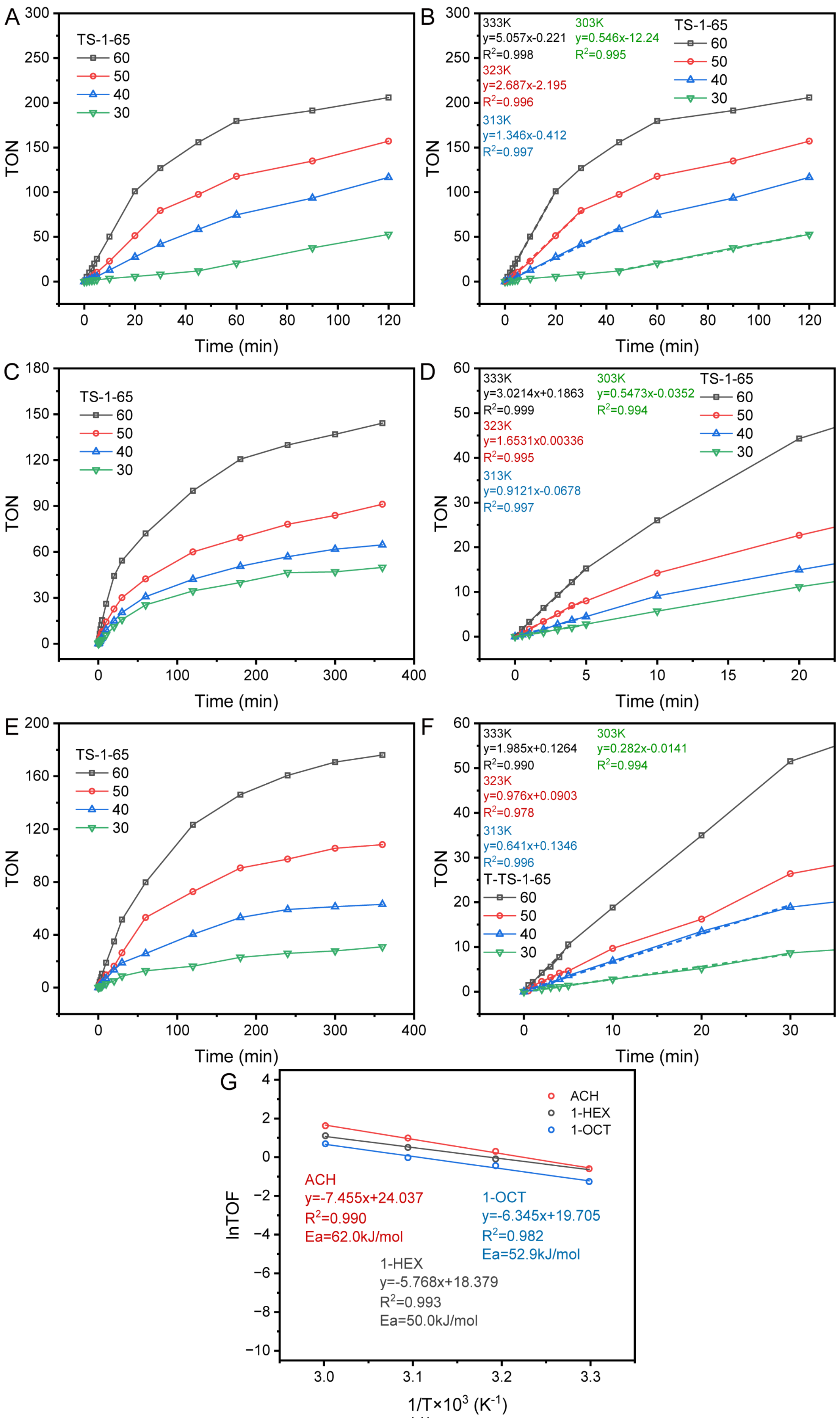

A
TS-1-65
60
50
40
30
TON
Time (min)
B
333K
y=5.057x-0.221
R2=0.998
323K
y=2.687x-2.195
R2=0.996
313K
y=1.346x-0.412
R2=0.997
303K
y=0.546x-12.24
R2=0.995
TS-1-65
60
50
40
30
TON
Time (min)
C
TS-1-65
60
50
40
30
TON
Time (min)
D
333K
y=3.0214x+0.1863
R2=0.999
323K
y=1.6531x0.00336
R2=0.995
313K
y=0.9121x-0.0678
R2=0.997
303K
y=0.5473x-0.0352
R2=0.994
TS-1-65
60
50
40
30
TON
Time (min)
E
TS-1-65
60
50
40
30
TON
Time (min)
F
333K
y=1.985x+0.1264
R2=0.990
323K
y=0.976x+0.0903
R2=0.978
313K
y=0.641x+0.1346
R2=0.996
303K
y=0.282x-0.0141
R2=0.994
T-TS-1-65
60
50
40
30
TON
Time (min)
G
ACH
1-HEX
1-OCT
ACH
y=-7.455x+24.037
R2=0.990
Ea=62.0kJ/mol
1-OCT
y=-6.345x+19.705
R2=0.982
Ea=52.9kJ/mol
1-HEX
y=-5.768x+18.379
R2=0.993
Ea=50.0kJ/mol
lnTOF
1/T×103 (K-1)

Figure S44. Reaction process (A) and pseudo-first order reaction kinetics fittings (B) of allyl chloride epoxidation, reaction process (C) and pseudo-first order reaction kinetics fittings (D) of 1-hexene epoxidation, reaction process (E) and pseudo-first order reaction kinetics fittings (F) of 1-octene epoxidation and Arrhenius plots of allyl chloride, 1-hexene and 1-octene epoxidation (G) in TS-1-65. Reaction conditions: catalyst 50 mg, olefin 10 mmol, H_2O_2 10 mmol, methanol 10 mL. The temperatures change from 303 to 333 K at 10 K interval.

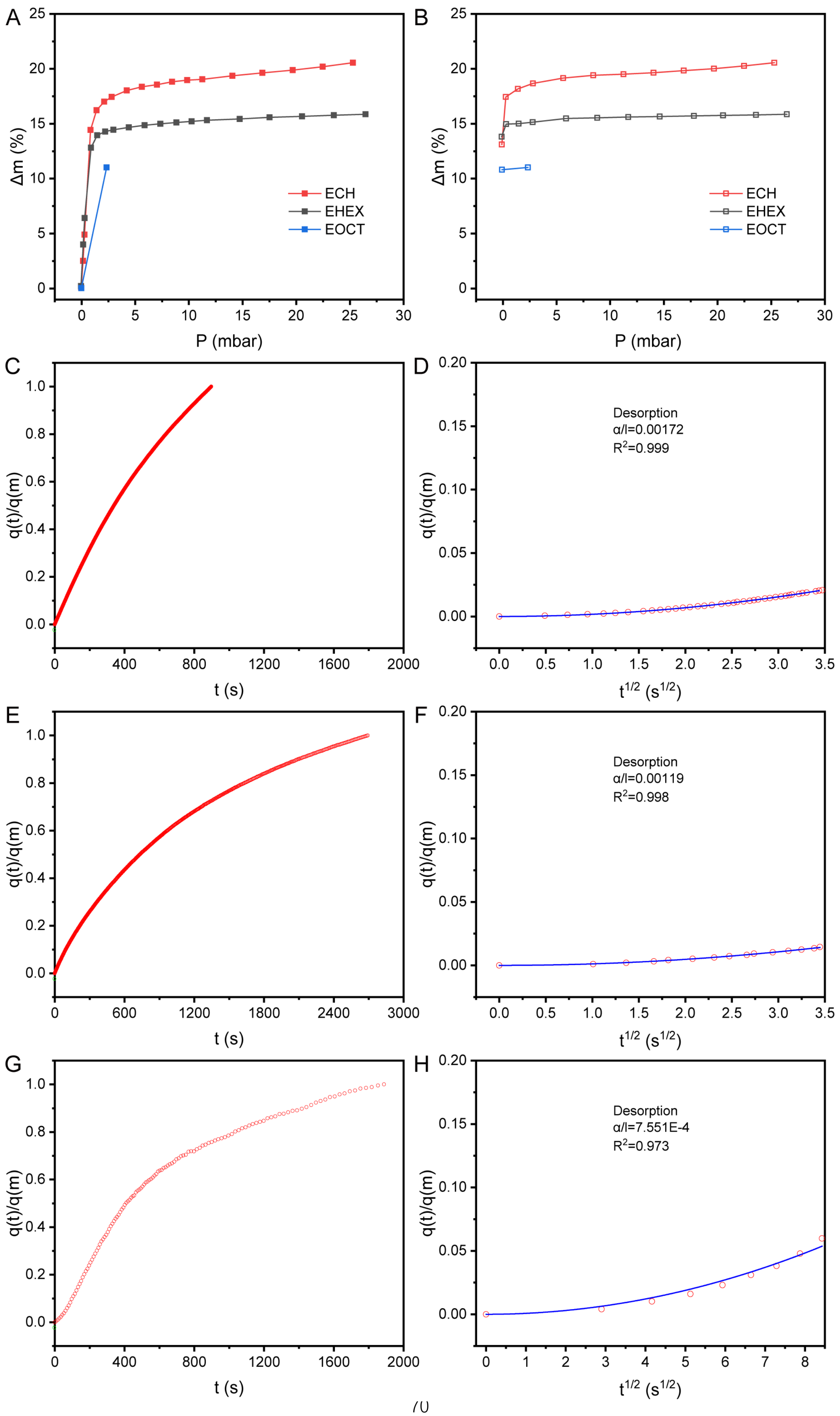
A
B
C
D
E
F
G
H
Δm (%)
P (mbar)
ECH
EHEX
EOCT
q(t)/q(m)
t (s)
t^1/2 (s^1/2)
Desorption
α/l=0.00172
R2=0.999
Desorption
α/l=0.00119
R2=0.998
Desorption
α/l=7.551E-4
R2=0.973

Figure S45. Adsorption (A) and desorption (B) isotherms of epichlorohydrin, 1,2-epoxyhexane and 1,2-epoxyoctane in TS-1-65. Kinetic uptake curves of epichlorohydrin (C), 1,2-epoxyhexane (E) and 1,2-epoxyoctane (G) and kinetic uptake curves and kinetic fitting profiles with SBM of epichlorohydrin (D), 1,2-epoxyhexane (F) and 1,2-epoxyoctane (H) in a short time domain in TS-1-65. The pressure range of epichlorohydrin and 1,2-epoxyhexane adsorption kinetic uptake curves is from 0 to 0.15 mbar. The pressure range of epichlorohydrin and 1,2-epoxyhexane desorption kinetic uptake curves is from 0.26 mbar to 0. The pressure range of 1,2-epoxyoctane adsorption kinetic uptake curves is from 0 to 2.27 mbar. The pressure range of 1,2-epoxyoctane desorption kinetic uptake curves is from 2.27 mbar to 0. The green circles and red circles are the experimental data of adsorption and desorption, respectively. The solid lines refer to the fitting curves. The parameter $\frac{\alpha}{l}$ refers to that in SBM.

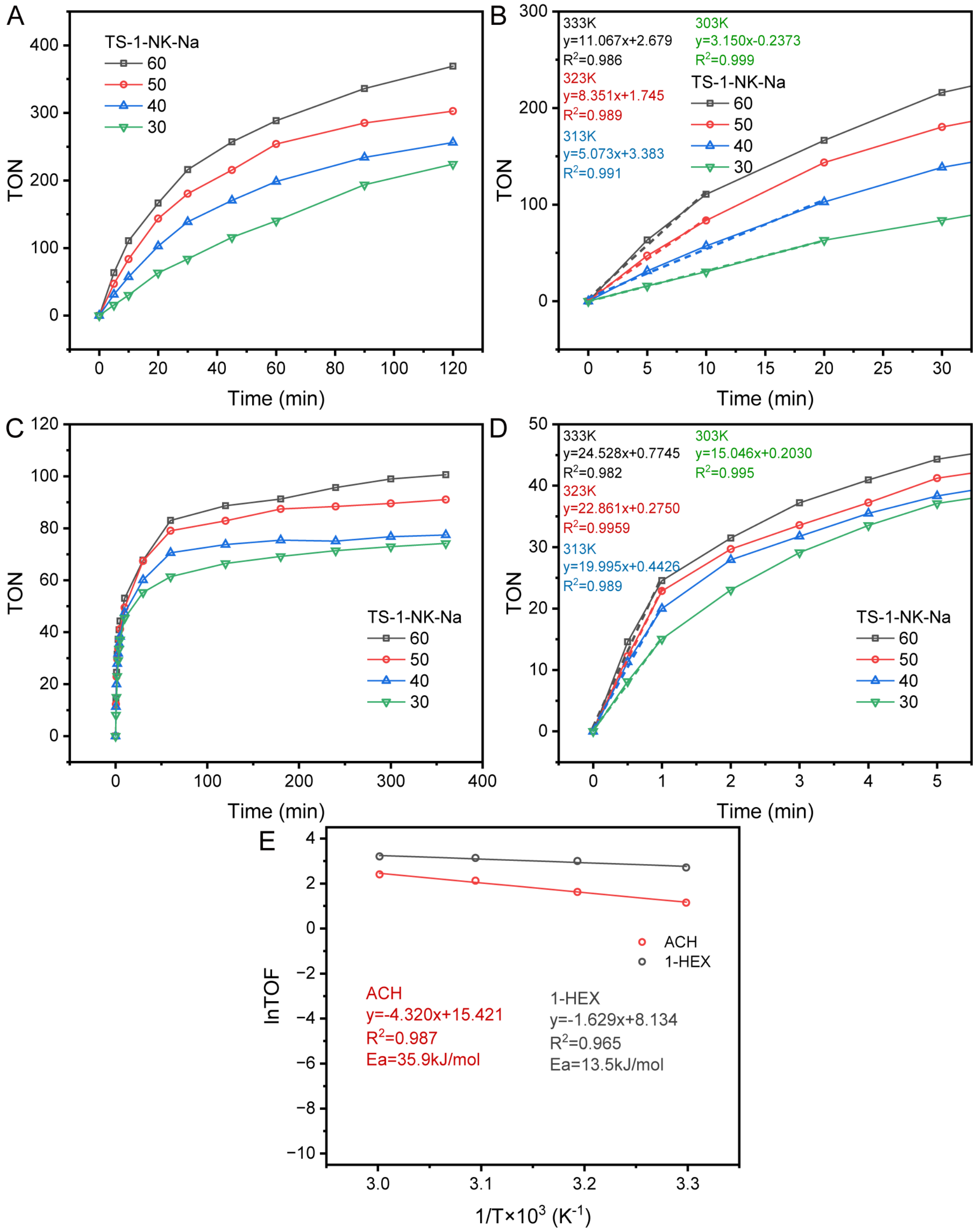

Figure S46. Reaction process (A) and pseudo-first order reaction kinetics fittings (B) of allyl chloride epoxidation and reaction process (C) and pseudo-first order reaction kinetics fittings (D) of 1-hexene epoxidation and Arrhenius plots of allyl chloride, 1-hexene epoxidation (E) in TS-1-NK-Na. Reaction conditions: catalyst 50 mg, olefin 10 mmol, H_2O_2 10 mmol, methanol 10 mL. The temperatures change from 303 to 333 K at 10 K interval.

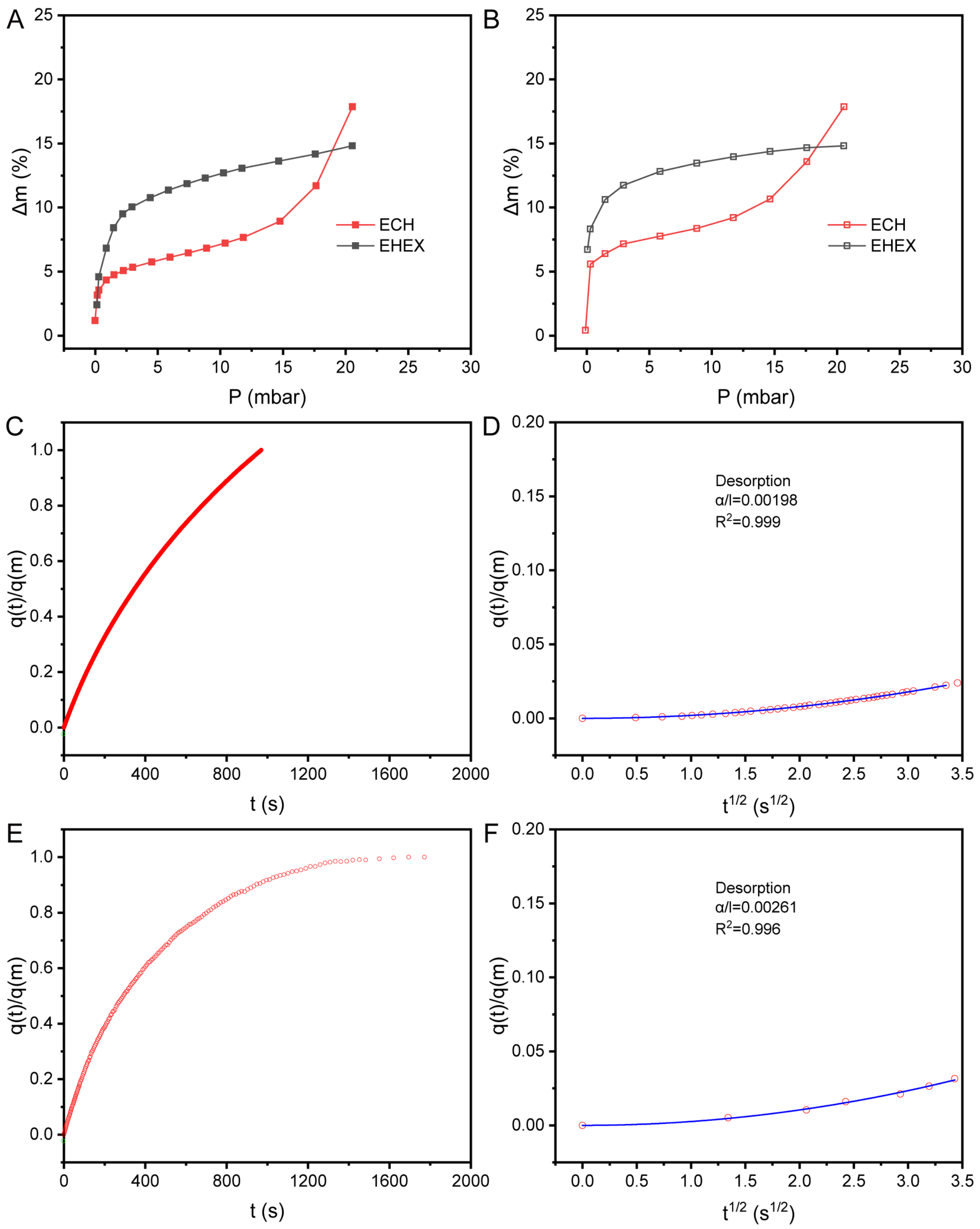

Figure S47. Adsorption (A) and desorption (B) isotherms of epichlorohydrin and 1,2-epoxyhexane in TS-1-NK-Na. Kinetic uptake curves of epichlorohydrin (C) and 1,2-epoxyhexane (E) and kinetic uptake curves and kinetic fitting profiles with SBM of epichlorohydrin (D) and 1,2-epoxyhexane (F) in a short time domain in TS-1-NK-Na. The pressure range of adsorption kinetic uptake curves is from 0 to 0.15 mbar. The pressure range of desorption kinetic uptake curves is from 0.26 mbar to 0. The red

circles are the experimental data of desorption. The solid lines refer to the fitting curves. The parameter $\frac{\alpha}{l}$ refers to that in SBM.

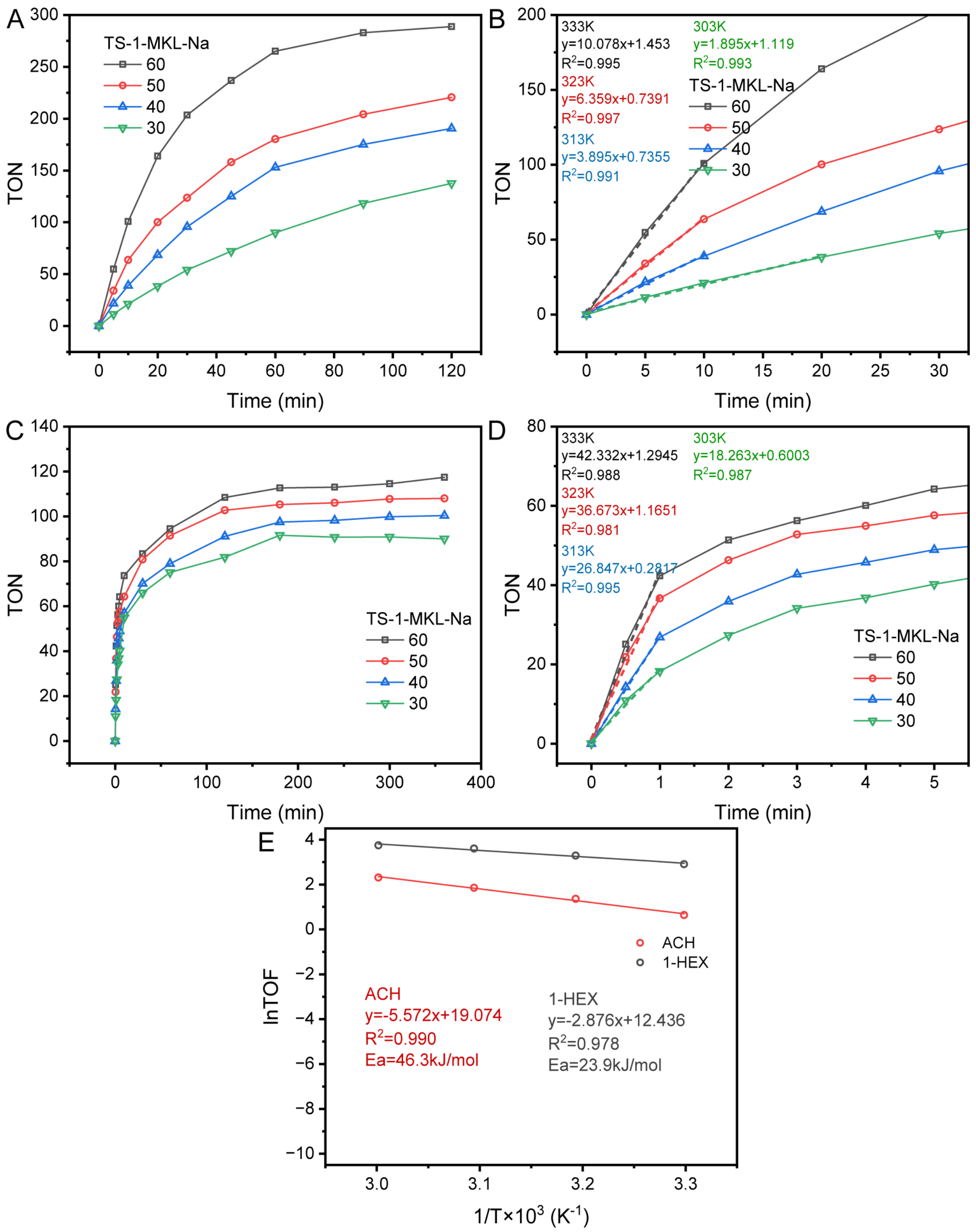

Figure S48. Reaction process (A) and pseudo-first order reaction kinetics fittings (B) of allyl chloride epoxidation and reaction process (C) and pseudo-first order reaction kinetics fittings (D) of 1-hexene epoxidation and Arrhenius plots of allyl chloride, 1-hexene epoxidation (E) in TS-1-MKL-Na. Reaction conditions: catalyst 50 mg, olefin 10 mmol, H_2O_2 10 mmol, methanol 10 mL. The temperatures change from 303 to 333 K at 10 K interval.

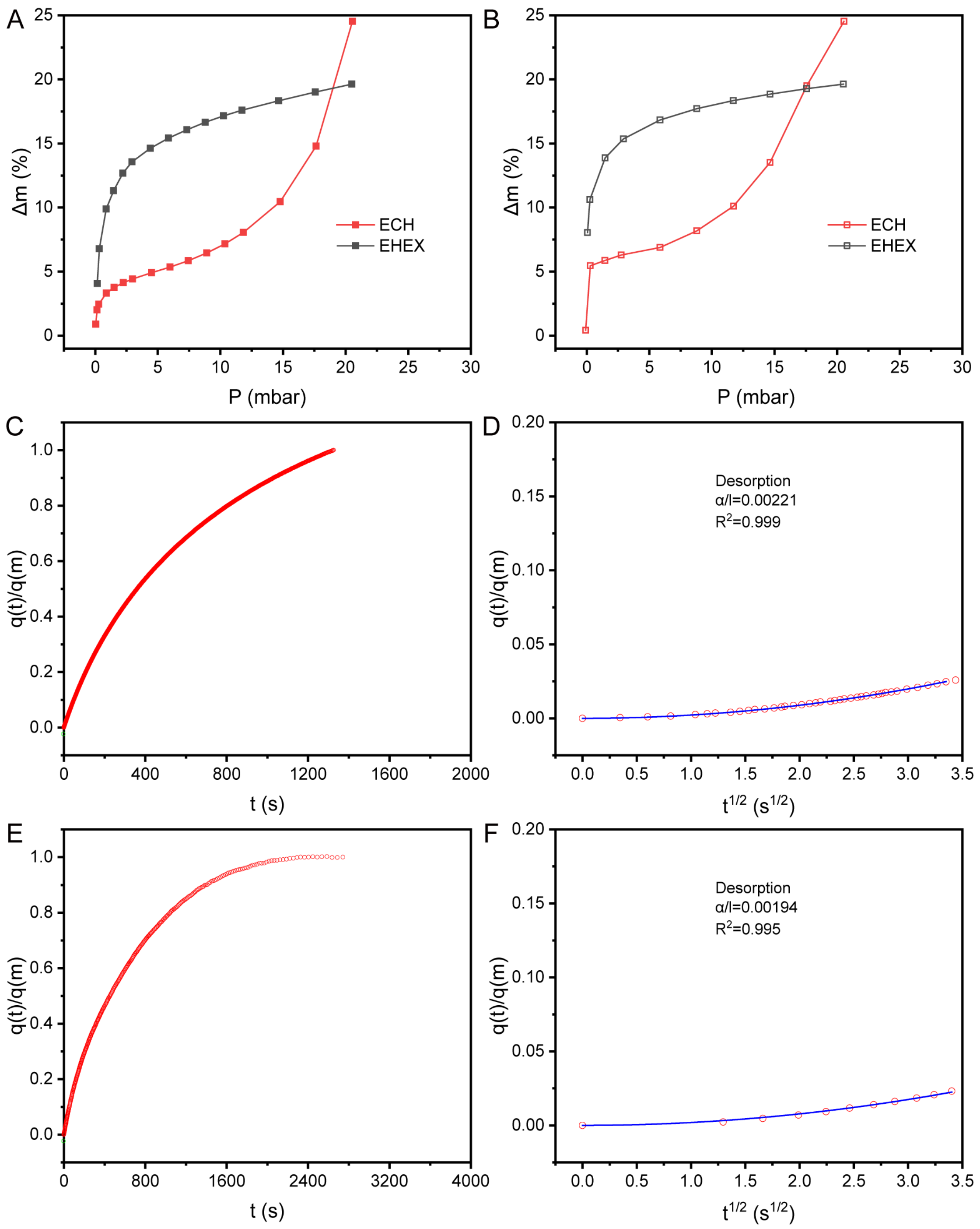

Figure S49. Adsorption (A) and desorption (B) isotherms of epichlorohydrin and 1,2-epoxyhexane in TS-1-MKL-Na. Kinetic uptake curves of epichlorohydrin (C) and 1,2-epoxyhexane (E) and kinetic uptake curves and kinetic fitting profiles with SBM of epichlorohydrin (D) and 1,2-epoxyhexane (F) in a short time domain in TS-1-MKL-Na. The pressure range of adsorption kinetic uptake curves is from 0 to 0.15 mbar. The pressure range of desorption kinetic uptake curves is from 0.26 mbar to 0. The red

circles are the experimental data of desorption. The solid lines refer to the fitting curves. The parameter $\frac{\alpha}{l}$ refers to that in SBM.

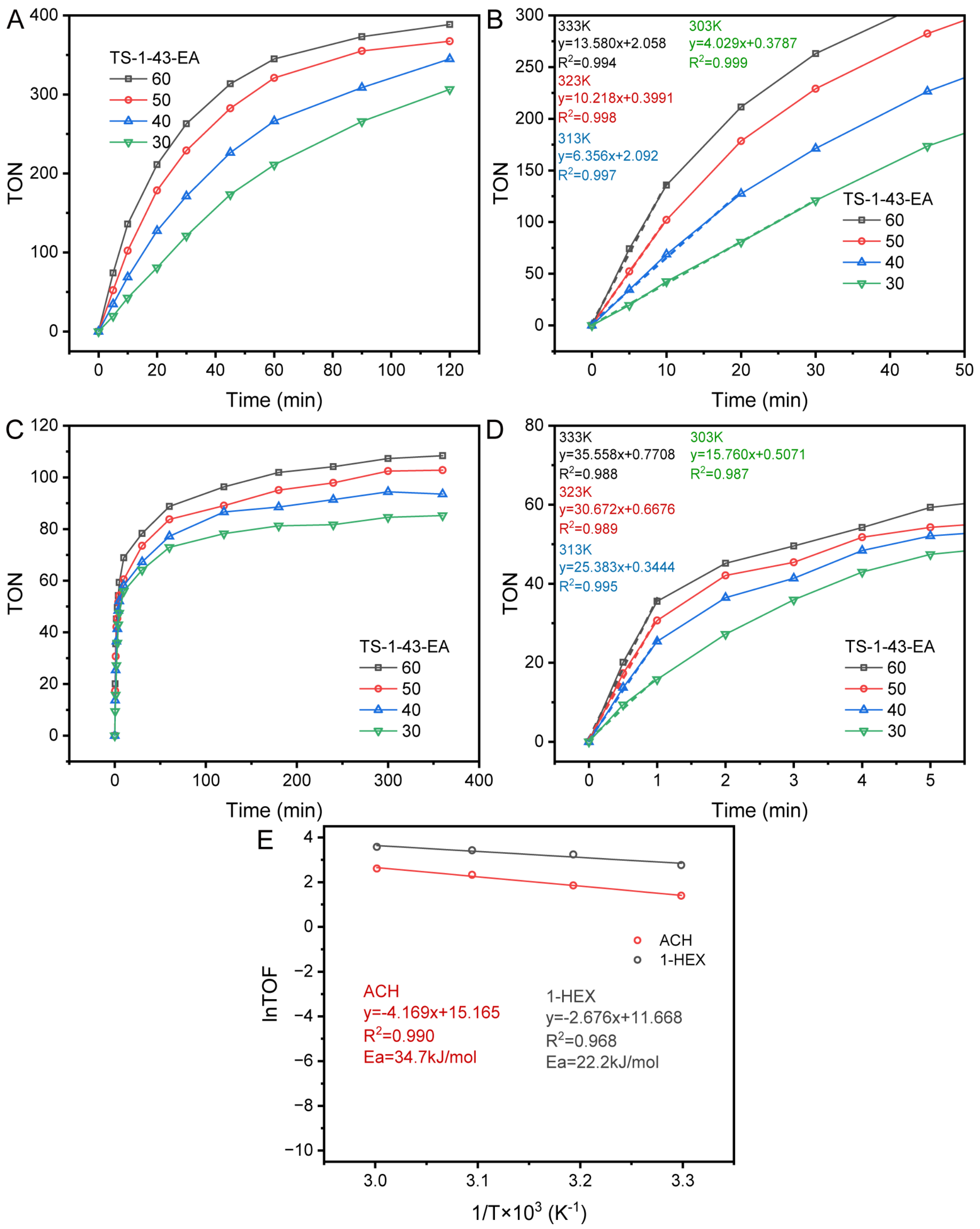

Figure S50. Reaction process (A) and pseudo-first order reaction kinetics fittings (B) of allyl chloride epoxidation and reaction process (C) and pseudo-first order reaction kinetics fittings (D) of 1-hexene epoxidation and Arrhenius plots of allyl chloride, 1-hexene epoxidation (E) in TS-1-43-EA. Reaction conditions: catalyst 50 mg, olefin 10 mmol, H_2O_2 10 mmol, methanol 10 mL. The temperatures change from 303 to 333 K at 10 K interval.

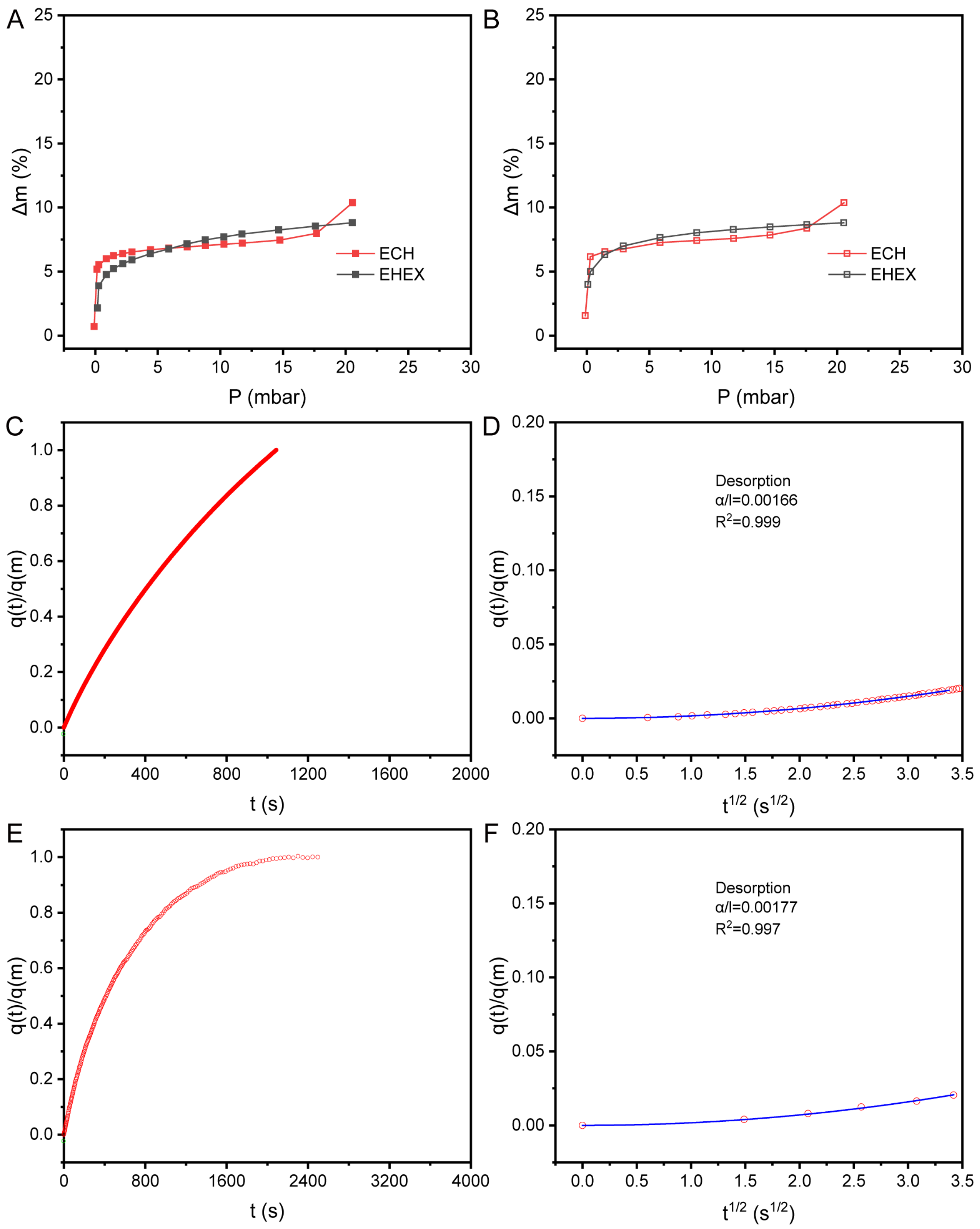

Figure S51. Adsorption (A) and desorption (B) isotherms of epichlorohydrin and 1,2-epoxyhexane in TS-1-43-EA. Kinetic uptake curves of epichlorohydrin (C) and 1,2-epoxyhexane (E) and kinetic uptake curves and kinetic fitting profiles with SBM of epichlorohydrin (D) and 1,2-epoxyhexane (F) in a short time domain in TS-1-43-EA. The pressure range of adsorption kinetic uptake curves is from 0 to 0.15 mbar. The pressure range of desorption kinetic uptake curves is from 0.26 mbar to 0. The red

circles are the experimental data of desorption. The solid lines refer to the fitting curves. The parameter $\frac{\alpha}{l}$ refers to that in SBM.

Table S1. Quantity of SC sol and initial synthetic sol added in synthesis system and the corresponding initial non-classical contribution

	TS-1-43	TS-1-71	TS-1-93	TS-1-100
SC sol (g) [a]	0	7.5	10	10
Initial synthetic sol (g) [a]	10	2.5	0.5	0
Initial non-classical contribution	43%	71%	93%	100%

[a] Added in the batch in one typical run of synthesis.

Table S2. Calculated cell parameters of TS-1 samples from HAXRD patterns

Cell parameter [a]	TS-1-43	TS-1-71	TS-1-91	TS-1-100
a (Å)	19.912	19.917	19.925	19.937
b (Å)	20.111	20.109	20.115	20.124
c (Å)	13.395	13.398	13.405	13.413
β (°)	90.25	90.07	90.01	90.00

[a] Calculated based on the cif file of H-ZSM-5_monoclinic from the IZA database with the P 1 21/n 1 space group [2].

Together with the increasing non-classical crystallization contribution, the β angle of P21 monoclinic crystal symmetry system decreases rapidly from 90.25° to 90° while the length of a, b and c expand for about 1‰ from TS-1-43 to TS-1-100.

Table S3. The estimated Ti site proportion in TS-1 samples

Sample	Titanium sites distribution				
	UV-vis results		XPS results		
	$[TiO_4]$ [a]	$[TiO_{5-6}]$ [b]	$[TiO_4]$ [c]	$[TiO_5]$ [d]	$[TiO_6]$ [e]
TS-1-43	72%	28%	56%	0%	44%
TS-1-71	87%	13%	63%	0%	37%
TS-1-93	85%	15%	75%	0%	25%
TS-1-100	62%	38%	56%	21%	23%

[a] Measured from the intensity of splited peaks around 205 nm in UV-vis spectra.

[b] Measured from the intensity of splited peaks around 265 nm in UV-vis spectra.

[c] Measured from the intensity of splited peaks at 460.2 eV in XPS spectra.

[d] Measured from the intensity of splited peaks at 459.0 eV in XPS spectra.

[e] Measured from the intensity of splited peaks at 458.5 eV in XPS spectra.

Table S4. $\frac{\alpha}{l}$ values for different adsorbates in TS-1 samples

$\frac{\alpha}{l}$ (10^{-3} s^{-1}) [a]	TS-1-43	TS-1-71	TS-1-93	TS-1-100
ACH-adsorption	11.41	13.20	11.44	11.58
ACH-desorption	2.99	3.71	3.56	3.23
ECH-adsorption	11.25	10.41	11.35	7.97
ECH-desorption	1.46	1.55	1.69	1.68
1-HEX-adsorption	9.50	8.49	9.48	12.15
1-HEX-desorption	2.29	2.33	2.68	2.28
EHEX-adsorption	10.91	10.89	11.82	9.58
EHEX-desorption	1.23	1.13	1.14	1.02
1-OCT-adsorption	11.90	13.39	9.82	8.67
1-OCT-desorption	7.88	4.03	2.40	3.66

[a] Calculated from kinetic uptake curves and kinetic fitting profiles with SBM in a short time domain. A higher $\frac{\alpha}{l}$ value indicates a faster adsorption or desorption diffusion rate.

References

[1] D. Pan, L. Kong, H. Zhang, Y. Zhang, Y. Tang, *ACS Applied Materials & Interfaces* **2023**, *15*, 28125-28134.

[2] H. van Koningsveld, J. C. Jansen, H. van Bekkum, *Zeolites* **1990**, *10*, 235-242.
